# Unveiling Novel Viral Diversity, Biogeography, and Host Networks in Wildlife Through High‐Throughput Sequencing Data Mining

**DOI:** 10.1002/advs.202511920

**Published:** 2025-09-23

**Authors:** Hai Wang, Yafei Meng, Xiaoyuan Chen, Xinyuan Cui, Qian Zuo, Na Han, Xianghui Liang, Xuejuan Shen, Caiwu Li, Desheng Li, Fumin Wang, Liangping He, Rujian Chen, Xingbang Lu, Wenjie You, Aiping Wu, Rui‐Ai Chen, Wu Chen, Chan Ding, Yongyi Shen

**Affiliations:** ^1^ School of Agriculture and Biology Shanghai Jiao Tong University Shanghai 200240 China; ^2^ State Key Laboratory for Animal Disease Control and Prevention Center for Emerging and Zoonotic Diseases College of Veterinary Medicine South China Agricultural University Guangzhou 510642 China; ^3^ State Key Laboratory of Common Mechanism Research for Major Diseases Suzhou Institute of Systems Medicine Chinese Academy of Medical Sciences & Peking Union Medical College Suzhou Jiangsu 215123 China; ^4^ Guangzhou Zoo & Guangzhou Wildlife Research Center Guangzhou 510070 China; ^5^ Zhaoqing Branch Center of Guangdong Laboratory for Lingnan Modern Agricultural Science and Technology Zhaoqing 526238 China; ^6^ China Conservation and Research Center for the Giant Panda Key Laboratory of State Forestry and Grassland Administration on the Giant Panda Chengdu 610066 China; ^7^ Guangdong Provincial Wildlife Monitoring and Rescue Center Guangzhou 510520 China

**Keywords:** novel virus, virome, wildlife, zoonotic

## Abstract

≈75% of emerging pathogens originating from wildlife. However, viral diversity within wildlife remains insufficiently explored. This work performs an extensive analysis of 57 536 publicly high‐throughput sequencing datasets from wild mammals and birds, resulting in the generation of ≈613.45 million assembled contigs, including 131 509 potential viral contigs identified through BLASTn and BLASTx searches. Following the exclusion of index hopping contamination, 9788 are categorized into 25 viral families with known zoonotic potential. These results indicate significant spatial and host‐specific variability in viral distribution and reveal a positive correlation between viral diversity and host biodiversity. Rodents, bats, ungulates, and anseriformes exhibit the highest viral diversity. Notably, 50% of the viral sequences exhibit <90% amino acid identity to known viruses, indicating of potential novel viruses. Host‐virus network uncovers 458 associations, 67.9% are unreported. Further, sequences of avian influenza viruses are identified in goats, while SARS‐CoV‐2 are detected in goats, ferrets, porpoises, cactus mice, and house finches. These findings highlight the largely uncharacterized viral diversity in wildlife, underscore the urgent requirement for surveillance at the wildlife‐livestock interfaces. Additionally, this work develop the Animal Pathogen Decoding Platform, to facilitate the retrieval and analysis of viral contigs, thereby reducing computational redundancies in future research.

## Graphical abstract

Analysis of 57 536 high‐throughput sequencing datasets uncovers a vast, hidden world of viruses in wildlife. The researchers reveal significant geographic and host‐specific patterns of viruses, and their surprising cross‐species transmissions, such as avian flu viruses infecting goats. This study highlights an urgent need for surveillance at the wildlife‐livestock interface and provides the Animal Pathogen Decoding Platform for future viral discovery.

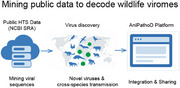

## 1 Introduction

Wildlife is considered to harbor a high diversity of unknown pathogens, with nearly three‐quarters of zoonotic diseases originating from wildlife.^[^ ^1^ ^]^ Over the past few decades, numerous outbreaks of novel zoonotic viruses, such as Severe acute respiratory syndrome coronavirus, Middle East respiratory syndrome coronavirus, Ebola virus, and most recentlySevere acute respiratory syndrome coronavirus 2 (SARS‐CoV‐2), have underscored the devastating impact of zoonotic diseases.^[^ ^1^, ^2^, ^3^ ^]^ World Health Organization emphasize that future viral outbreaks of “Disease X” are inevitable, emphasizing the need for preparedness against unknown pathogens.^[^ ^4^ ^]^ In this context, proactive surveillance of viral diversity in animal populations to identify potential high‐risk pathogens before they achieve efficient human transmission is crucial for early warning and pandemic preparedness.

Among wildlife, mammals and birds are particularly recognized as critical sources of novel zoonotic viruses, making them focal points for viral surveillance.^[^ ^5^ ^]^ Current estimates suggest that mammalian and avian hosts harbor up to 1.7 million undiscovered viruses, many of which may pose zoonotic risks.^[^ ^6^ ^]^ Advances in high‐throughput sequencing (HTS) technologies have revolutionized virus discovery, enabling large‐scale characterization of viral genomes from diverse animal hosts.^[^ ^7^ ^]^ More recent large‐scale virological sampling and sequencing of wildlife have led to the discovery of numerous novel viruses and potential cross‐species transmission events, particularly in bats, rodents, and birds.^[^ ^8^, ^9^, ^10^, ^11^ ^]^ However, our understanding of the virosphere remains nascent.^[^ ^12^ ^]^

Numerous HTS datasets, initially not intended for viral research. For instance, transcriptomic studies primarily investigate host gene expression, while metagenomic analyses often target antibiotic‐resistant bacteria. These repositories provide an invaluable resource for secondary data analysis, allowing researchers to mine existing datasets for novel or understudied viruses.^[^ ^13^, ^14^, ^15^, ^16^, ^17^ ^]^ Another critical limitation is that the public databases (e.g., National Center for Biotechnology Information, NCBI; DNA Data Bank of Japan; European Molecular Biology Laboratory) emphasis on raw data deposition. The assembled viral contigs from most HTS datasets are not available. Therefore, subsequent studies from different laboratories must independently perform de novo assembly of these huge Sequence Read Archive (SRA) data, leading to significant computational resource waste and severely hindering viral data integration as well as the efficient retrieval of emerging viruses. For example, although reads of SARS‐CoV‐2‐related coronaviruses were included in SRAs of pangolins prior to the coronavirus disease 2019 (COVID‐19) pandemic, only the raw HTS data rather than the assemble contigs of SARS‐CoV‐2‐related coronavirus was deposited to the NCBI.^[^ ^18^ ^]^ This greatly hindered the identification of the SARS‐CoV‐2‐related coronavirus after the emergence of COVID‐19.

The significance of decoding viromes in wildlife has garnered considerable attention; however, existing studies are constrained by the limited scope of sample collection, leading to a rudimentary understanding of the viruses present in these populations. The publicly available HTS data encompasses samples from various species and multiple temporal contexts globally, offering invaluable resources for advancing our comprehension of viral diversity in wildlife. Consequently, this study utilizes publicly accessible HTS data from wild mammals and birds to systematically identify and characterize the previously concealed diversity of viruses, as well as unrecognized cross‐species transmission events. Further, to facilitate future surveillance studies, we have also developed the Animal Pathogen Decoding Platform (AniPathoD), which is designed to provide assembled viral contigs from HTS data.

## 2 Result

### 2.1 Identification of Contamination in HTS Datasets

In order to identify significant viral taxa and develop a comprehensive global atlas of viral distribution, we meticulously curated and processed a total of 57 536 sequence read runs (SRRs) available in the NCBI database as of March 31, 2025 (Supporting Data 1). This effort resulted in the generation of ≈613.45 million assembled contigs, which were subsequently utilized for viral identification and classification.

**Figure 1:**
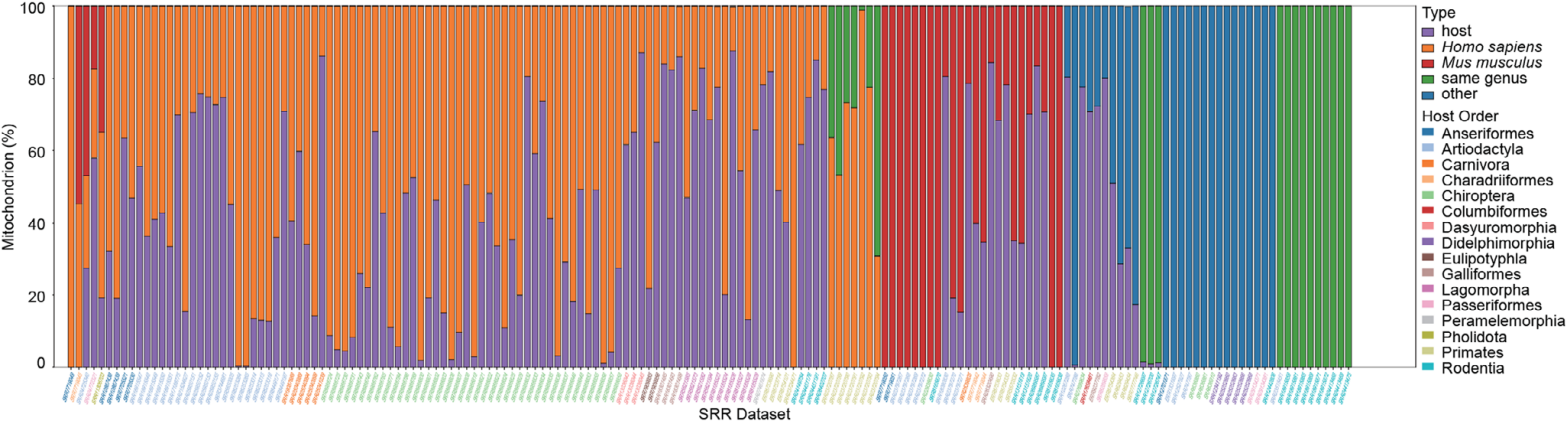
Distribution of different contamination types in SRR datasets. The stacked bar chart delineates the categorization and relative abundance of host reads identified in potentially contaminated SRR datasets.

We established a workflow, illustrated in Figure S1, Supporting Information, designed to identify any SRR datasets that may be contaminated. Clean reads of each SRR dataset underwent alignment against the vertebrate mitochondrial genome database. Initial screening revealed 887 datasets exhibiting a significant presence of mitochondrial sequences that were inconsistent with the documented species. Further validation using BLASTn analysis confirmed that 169 of these datasets were indeed contaminated (**Figure** **1** and Supporting Data 2). The discrepancies observed in relation to NCBI records were classified into four distinct categories: (1) contamination from human sources (107 cases, 63.3%); (2) contamination from murine sources (28 cases, 16.6%); (3) contamination from species within the same genus as the recorded species (20 cases, 11.8%); and (4) contamination from various other species (25 cases, 14.8%). While four cases have contamination from both human and murine sources, seven cases have contamination from both human and species within the same genus as the recorded species.

### 2.2 Identification and Characterization of Viral Contigs in Wild Mammals and Birds

**Figure 2:**
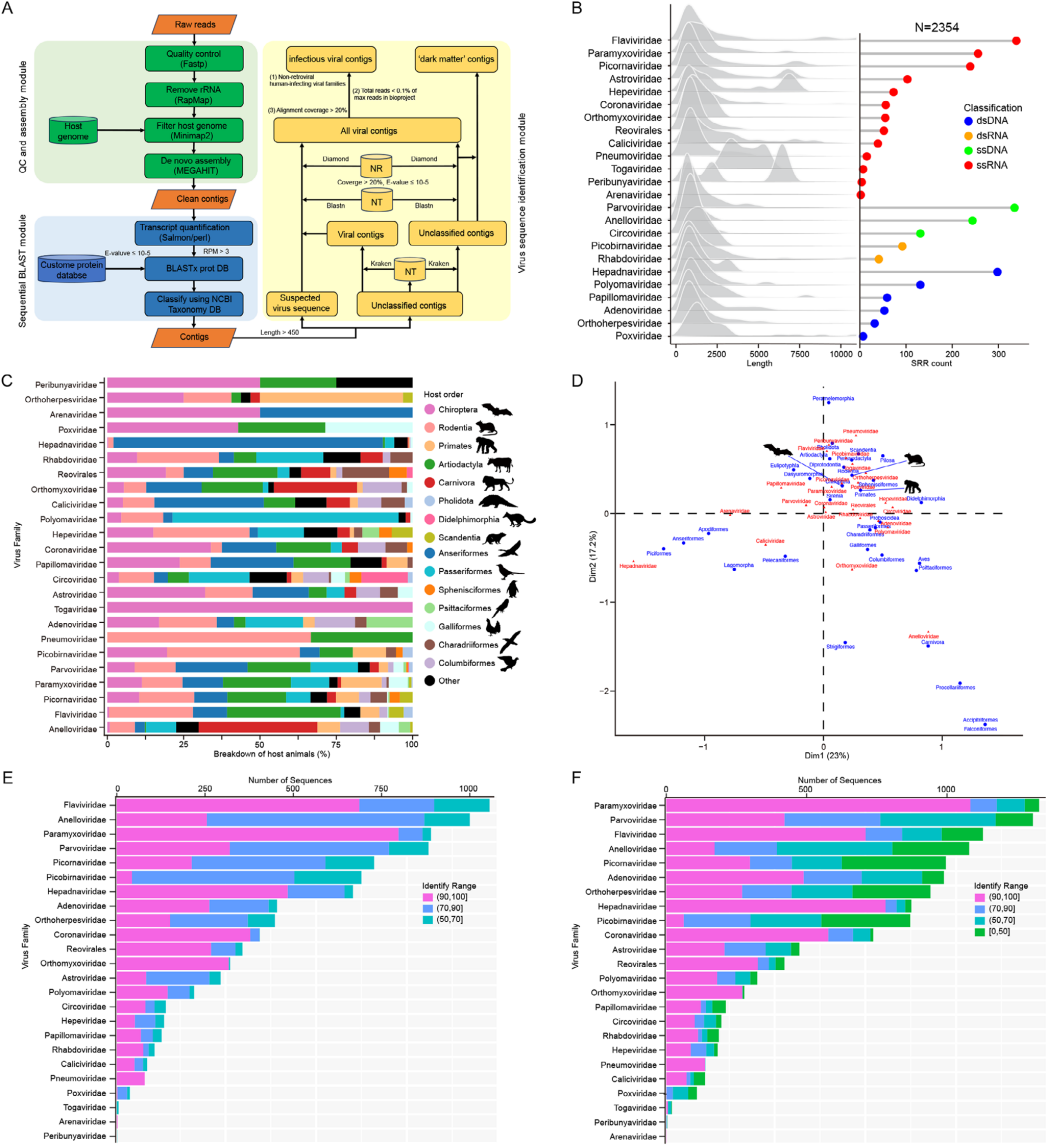
Identification and characterization of viral sequences from wildlife. A) The schematic diagram outlines the methodology employed for the detection of viral contigs, encompassing stages such as data acquisition, sequence assembly, virus annotation, contamination filtering, and classification. B) The left panel presents a density map depicting the distribution of viral sequence lengths, while the right panel indicates the number of SRR datasets in which viral families known to infect humans were identified. C) The taxonomic distribution of hosts associated with the detected viral families is displayed, with the x‐axis representing the proportion of host orders linked to each viral family and the y‐axis denoting the viral families themselves. Distinct colors are utilized to differentiate various host orders. D) A correspondence analysis between hosts and viruses is presented, plotting viral families and host orders according to their interrelations, with red labels denoting viral families and blue labels representing host orders. E,F) A comparative analysis of the similarity of identified viral sequences with the NCBI nt (E) and nr (F) databases. In these plots, the x‐axis indicates the number of viral sequences, while the y‐axis represents the viral families, with colors reflecting the ranges of percentage identity of the detected sequences. No sequences corresponding to the Filoviridae family were detected, therefore the data pertaining to the remaining 24 viral families have been presented.

Following the exclusion of potentially contaminated datasets, we established a methodology for the detection of viral contigs (**Figure** **2**A). The analysis focused on 25 viral families known to infect humans, which include Adenoviridae, Anelloviridae, Arenaviridae, Astroviridae, Peribunyaviridae, Caliciviridae, Circoviridae, Coronaviridae, Filoviridae, Flaviviridae, Hepadnaviridae, Hepeviridae, Orthoherpesviridae, Orthomyxoviridae, Papillomaviridae, Paramyxoviridae, Parvoviridae, Picobirnaviridae, Picornaviridae, Pneumoviridae, Polyomaviridae, Poxviridae, Reoviridae, Rhabdoviridae, and Togaviridae.

After filtering for index‐hopping contamination and establishing a threshold for viral infection, a total of 9788 potential infectious viral sequences were identified. These sequences varied in length from 450 to 83 144 bp, with an average length of 2027 bp, and were sourced from 2354 SRRs across 332 host species. The viral families that were most commonly identified comprised Flaviviridae (339 SRRs), Parvoviridae (335 SRRs), Hepadnaviridae (298 SRRs), and Paramyxoviridae (256 SRRs). While no sequences corresponding to the Filoviridae family were detected (Figure 2B). The highest proportions of the studied viral families were found in rodents, ungulates, bats, as well as in birds of the Anseriformes and Passeriformes (Figure 2C). Notably, correspondence analysis of viral families and host orders indicated a close clustering of rodents, bats, and primates, suggesting shared viral associations (Figure 2D).

When compared to viral sequences in the NCBI nucleotide (nt) and non‐redundant (nr) databases, 44% (4326/9788) of the viral sequences exhibited less than 90% nucleotide identity with known viruses, indicating the presence of novel viral coding sequences (Figure 2E,F). The high‐confidence genus‐level classification results obtained through Viral Taxonomic Assignment Pipeline^[^ ^19^ ^]^ revealed that 21% (2052/9788) of the viral sequences were classified as novel viruses (Supporting Data 3).

### 2.3 Global Distribution of Viral Diversity

**Figure 3:**
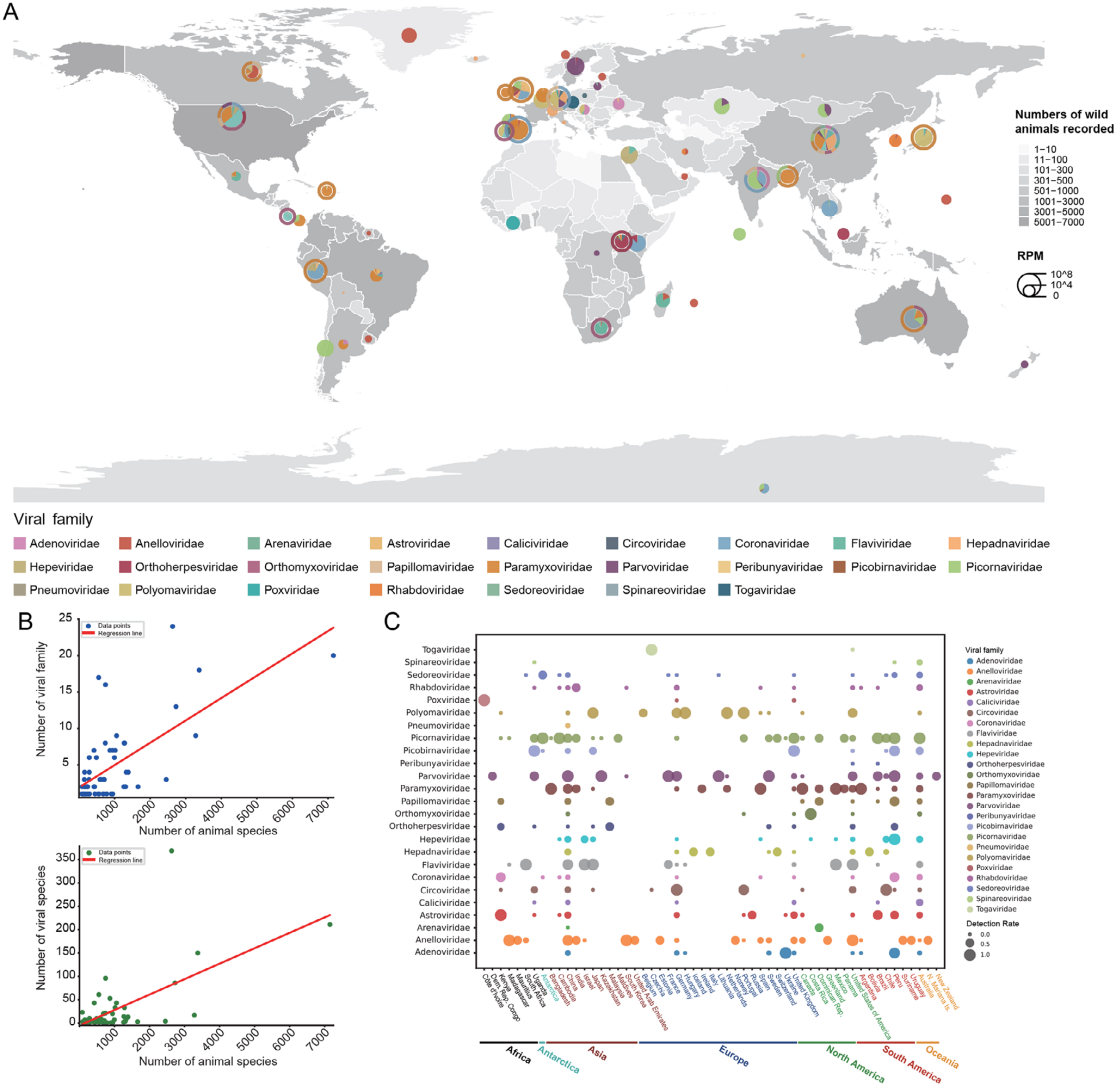
The global heterogeneity in the spatial distribution of viruses. A) The worldwide distribution of wild animals and associated viruses. The heatmap indicating the occurrence records of chordate species in various countries and regions; deeper shades of gray correspond to higher species counts. Additionally, the pie chart represents the distribution of viral families across different countries and regions, with the size of each pie chart reflecting the cumulative value of Reads per Million Mapped Reads (RPM). The outer ring of the pie chart depicts the relative proportions of viral families that are linked to potential zoonotic risks. B) Correlation analyses between the number of vertebrate species and the number of viral families or viral species. C) The detection rates of viral families across various countries.

Generally, countries characterized by high vertebrate species richness demonstrated elevated levels of viral diversity (**Figure** **3**A and Supporting Data 4). Quantitative analyses indicated a statistically significant positive correlation at both the national and regional levels between the number of vertebrate species and the number of viral families (*p* < 0.001, r^2^ = 0.42). A similar positive correlation was observed between the diversity of vertebrate species and the diversity of viral species (*p* < 0.001, r^2^ = 0.35, Figure 3B). To mitigate the potential effects of sampling bias, we conducted an additional subsampling validation. The positive correlation between vertebrate species richness and viral family richness persisted consistently, yielding a mean Spearman's correlation coefficient (ρ) of 0.465 (95% confidence interval: 0.269 to 0.638) (Figure S2A, Supporting Information). Conversely, at the viral species level, the mean Spearman's ρ was 0.063 (95% confidence interval: −0.135 to 0.251), suggesting a weak and statistically non‐significant relationship with host diversity (Figure S2B, Supporting Information). This discrepancy likely indicates that viral species richness is more susceptible to stochastic sampling effects compared to measures assessed at the family level. Spatial analyses of the 25 viral families identified substantial geographical variation in both viral diversity and distribution (Figure 3C). For example, the Paramyxoviridae family was found to be prevalent across various regions, including Asia (notably China, India, and Bangladesh), Europe (Sweden, the United Kingdom, and Germany), North America (the United States and Panama), South America (Brazil and Peru), and Oceania (Australia). Conversely, the Hepeviridae family was primarily observed in South America (Chile and Peru), Asia (China, Japan), Oceania (Australia), and Hepadnaviridae were mainly detected in certain areas of Europe (the United Kingdom and Sweden). However, the imbalances of data across different countries may bias this viral distribution pattern (Figure S3, Supporting Information).

### 2.4 Phylogenetic Analyses of Vertebrate‐Associated Viruses

**Figure 4:**
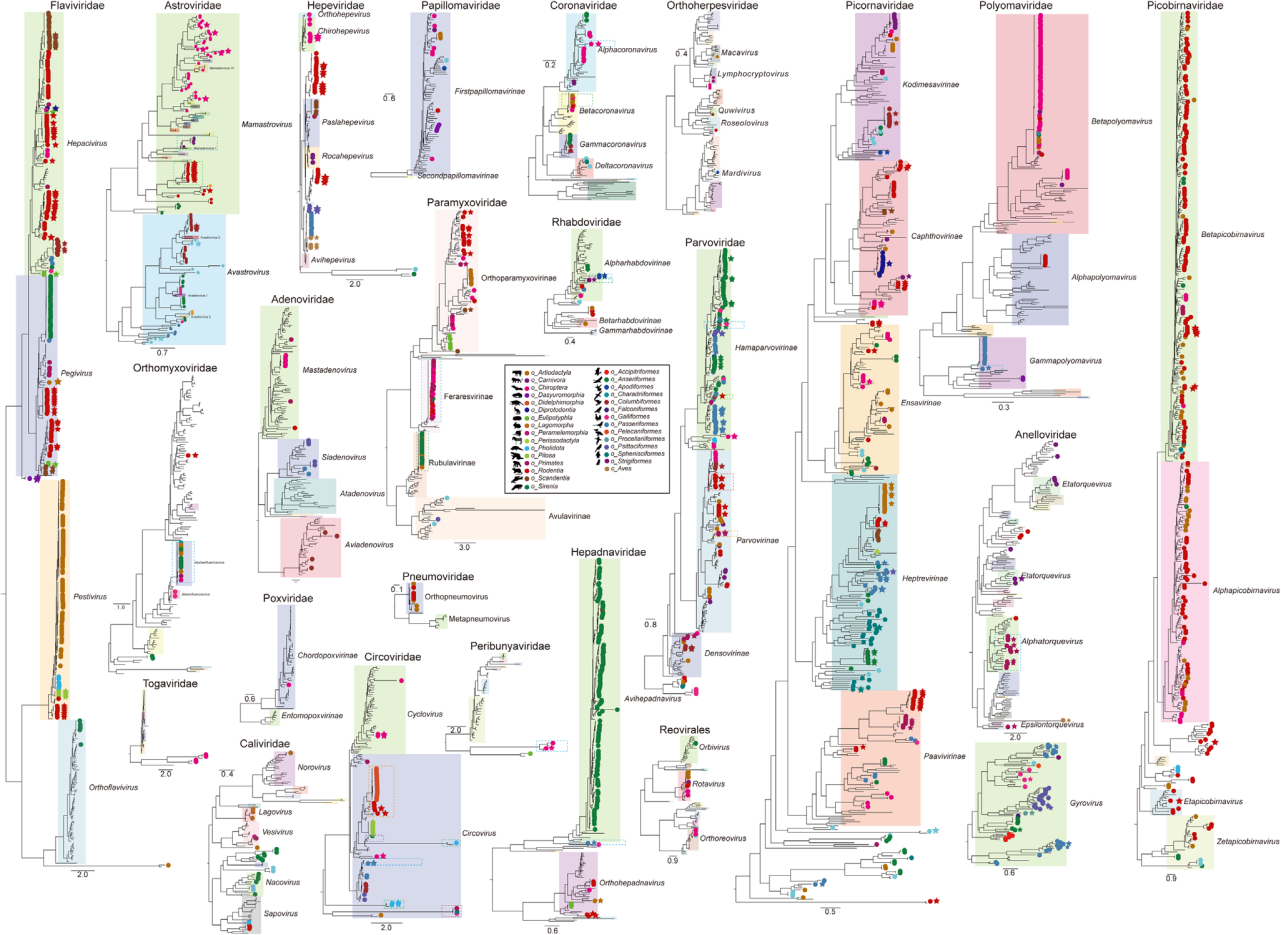
Phylogenetic trees of 23 viral families. The viruses identified in this research are indicated by solid circles, with their colors corresponding to the respective animal host orders. Additionally, 245 novel viruses with nearly complete genomes are denoted by five‐pointed stars. The scale bars represent the number of amino acid substitutions per site. Detailed trees are shown in Figures S4–S26, Supporting Information.

In accordance with International Committee on Taxonomy of Viruses (ICTV) guidelines, 1782 complete or partial gene sequences were extracted from viral contigs for phylogenetic analysis (**Figure** **4** and Supporting Data 5). The analysis revealed that the families Flaviviridae (283 sequences), Picobirnaviridae (237 sequences), and Picornaviridae (237 sequences) contained the highest number of sequences. This was followed by the families Hepadnaviridae (167 sequences), Parvoviridae (164 sequences), Astroviridae (112 sequences), Paramyxoviridae (107 sequences), Polyomaviridae (103 sequences), Anelloviridae (79 sequences), Hepeviridae (58 sequences), Circoviridae (55 sequences), Coronaviridae (35 sequences), Orthomyxoviridae (29 sequences), Caliciviridae (27 sequences), Adenoviridae (21 sequences), Papillomaviridae (16 sequences), Reovirales (13 sequences), Rhabdoviridae (13 sequences), Orthoherpesviridae (9 sequences), Pneumoviridae (9 sequences), Togaviridae (4 sequences), Peribunyaviridae (3 sequences), and Poxviridae (1 sequence). No sequences corresponding to the Filoviridae family were identified. While some sequences from the Arenaviridae family were detected (Figure 2B), these sequences did not encompass the *L* gene, which the ICTV has proposed as essential for constructing the phylogenetic tree of Arenaviridae. As a result, after excluding the Arenaviridae and Filoviridae families, only 23 phylogenetic trees were reconstructed (Figure 4).

Notably, 49% (867 out of 1782) of the viral sequences had less than 90% amino acid sequence identity to previously identified viruses. Among these, 245 novel viruses possessed nearly complete assembled genomes, which were subsequently categorized into 167 distinct viral species based on a 95% nucleotide identity threshold (denoted by five‐pointed stars in Figure 4, detail in Supporting Data 6).

Within the family Flaviviridae, a variety of viruses belonging to the genera *Pestivirus*, *Pegivirus*, and *Hepacivirus* were identified (Figure 4 and Figure S4, Supporting Information). To give a more detail topology, only those novel viruses with near‐complete genomes were used to reconstruct the phylogenetic tree based on the full‐length *RdRp* gene sequences (Figure S27, Supporting Information). Two novel pestivirus strains were discovered in two rodent species (*Sciurus carolinensis* and *Marmota monax*). Phylogenetic analysis indicated that these two rodent viruses did not align without any of the 19 currently recognized Pestivirus species (marked in red dashed box in Figure S27, Supporting Information). These viruses exhibited the closest phylogenetic relationship to atypical porcine pestivirus, sharing a maximum amino acid identity of 61% in the *RdRp* gene. Additionally, pegivirus strains were detected in three rodent species (*Ondatra zibethicus*, *Mesocricetus auratus*, and *Chinchilla lanigera*), one tree shrew (*Tupaia belangeri*), one even‐toed ungulate (*Antilocapra americana*) and one mole species (*Scalopus aquaticus*). Phylogenetic analysis revealed that the pegiviruses from long‐tailed chinchilla (*Chinchilla lanigera*) was the sister clader to bat‐associated pegivirus, while the pegivirus identified in *Antilocapra americana sonoriensis* was the sister clader to Equine pegivirus (marked in purple dashed box in Figure S27, Supporting Information). The pegiviruses identified from *Ondatra zibethicus*, *Mesocricetus auratus*, *Scalopus aquaticus*, and *Tupaia belangeri* grouped with rodent and bat pegiviruses, forming a sister clade to human hepegiviruses (marked in blue dashed box in Figure S27, Supporting Information). Notably, co‐infection with both Pegivirus and Hepacivirus was observed in a tree shrew individual (DRR155098, marked by black dotted arrow in Figure S27, Supporting Information).

Many novel hepaciviruses were detected in various rodents (Chinchilla lanigera, Tokudaia tokunoshimensis, Ondatra zibethicus, and Heteromys desmarestianus), moles (Nannospalax ehrenbergi and Scalopus aquaticus), a shrew (Blarina brevicauda), and a pigeon (Columba livia). Phylogenetic analysis revealed that hepaciviruses encompass a wide range of lineages, including those from mammals, birds, geckos, turtles and fish. Hepaciviruses identified from Scalopus aquaticus and Myrmecobius fasciatus were found to be distantly related to the mammalian clade, clustering with the turtle clade (marked in yellow dashed box in Figure S27, Supporting Information). Notably, co‐infections of two distinct clades (clade 1 and clade 2) of hepaciviruses were detected in four individuals of Nannospalax ehrenbergi (SRR15340415, SRR15340423, SRR15340431, and SRR1534049, marked in black dashed box in Figure S27, Supporting Information). These clades exhibited significant genetic divergence, with only 46% amino acid identity in RdRp gene. Furthermore, mixed infections of hepaciviruses of clade 1 and clade 2 were observed in four individuals (SRR15340415, SRR15340423, SRR15340431, and SRR15340439, marked in red dotted arrows in Figure S27, Supporting Information). The hepacivirus from Chinchilla lanigera was found to be cluster with GB virus‐B (marked in green dashed box in Figure S27, Supporting Information).

In the Astroviridae family, astroviruses were identified in various bat species, including *Miniopterus natalensis*, *Natalus macrourus*, *Rhinolophus eloquens*, *Mesophylla macconnelli* et al., as well as in bird species, such as *Columba livia*, *Arenaria interpres*, *Calidris ruficollis*, *Anser cygnoides* et al. Phylogenetic analysis revealed that these viruses clustered with known bat‐ and avian‐associated astroviruses, respectively (Figure 4 and Figure S5, Supporting Information). Five sequences from cats (*Felis silvestris*) clustered with the human astrovirus, exhibited 66%–85% amino acid identity (marked in blue dashed box in Figure S5, Supporting Information), while 11 sequences from wood mice (*Apodemus sylvaticus*) clustered with the porcine astrovirus, exhibited 63%–68% amino acid identity in the capsid protein (marked in green dashed box in Figure S5, Supporting Information).

Within the Parvoviridae family, a total of 34 near‐complete genomes of parvoviruses were identified in 7 mammalian and 10 avian hosts (Figure S6, Supporting Information). Remarkably, the Alectoris chukar parvovirus (SRR26796667) exhibited 72% amino acid identity with the Phasianus chaphama parvovirus in the NS1 protein (marked in blue dashed box in Figure S6, Supporting Information), which is the etiological agent of hepatitis outbreaks in pheasants (Phasianus colchicus) with high mortality rates.^[^ ^20^ ^]^ Sequences from a rodent (*Zapus princeps*) and a bear (*Ursus thibetanus*) had 85% amino acid identity in the NS1 protein (marked in green dashed box in Figure S6, Supporting Information). Five parvovirus sequences of voles (*Lasiopodomys mandarinus*) and two sequences of wood mice (*Apodemus sylvaticus*) clustered with canine parvoviruses with 87%–96% amino acid identity (marked in red dashed box in Figure S6, Supporting Information). Further, two genomes of parvoviruses detected from *Papio anubis* exhibited 89% amino acid identity with the human parvovirus (marked in yellow dashed box in Figure S6, Supporting Information).

In the Anelloviridae family, birds of Passeriformes, Anseriformes, Accipitriformes and Psittaciformes carried diverse Gyroviruses. 11 sequences of simian torque teno viruses were identified in Papio Anubis (marked in blue dashed box in Figure S7, Supporting Information). Their ORF1 proteins shared 40%–58% amino acid identity with known simian torque teno viruses.

In the Circoviridae family, the Lonchura striata circovirus (SRR9295882) shared up to 87% and 75% amino acid identity with the Raven circovirus (NC_0 08375) in the Rep and Cap proteins, respectively (marked in blue dashed box in Figure S8, Supporting Information). This circovirus has been associated with feather lesions that resemble those observed in psittacine beak and feather disease.^[^ ^21^ ^]^ Three circovirus sequences from Sunda pangolin (*Manis javanica*) were classified into two distinct clades, as indicated by the green dashed box in Figure S8, Supporting Information. Both clades exhibited a significant genetic divergence (0.68–0.91) from other circovirus sequences, clustering instead with environmental sequences (WAE42795 and QRI44249). Additionally, a sequence derived from *Lemur catta* clustered with those circoviruses derived from avian species, with 97%–100% amino acid identity (marked in red dashed box in Figure S8, Supporting Information). A sequence of circovirus from *Anser cygnoides* demonstrated a 90%–100% amino acid identity with the porcine circoviruses (marked in purple dashed box in Figure S8, Supporting Information). Circoviruses from opossum (*Monodelphis domestica*) and two rodents (*Oligoryzomys longicaudatus* and *Clethrionomys rutilus*) clustered with bat circovirus (ASU92187) and felid circovirus (QNS17424) (marked in yellow dashed box in Figure S8, Supporting Information).

Within the Hepadnaviridae family, avihepadnaviruses were detected in 144 SRR datasets derived from Anserformes birds. A near‐complete genome of hepadnavirus identified from Junco hyemalis showed a maximum of 47% amino acid identity with the Crane hepatitis B virus (CAD29590) in the polymerase gene, and formed a sister clade to the Avihepadnavirus group in the phylogenetic tree (marked in blue dashed box in Figure S9, Supporting Information). Pairwise p‐distance analysis revealed that the genetic distance between the Junco hyemalis hepadnavirus and other avihepadnaviruses (e.g., duck hepatitis B virus, heron hepatitis B virus, stork hepatitis B virus, crane hepatitis B virus, and parrot hepatitis B virus) ranged from 0.41 to 0.43, while intra‐group distances among avihepadnaviruses were less than 0.27. This suggests that the hepadnavirus identified in Junco hyemalis may represent a novel genus.

In the family Rhabdoviridae, the Otus sunia virus (SRR6650823), detected in the blood of Otus sunia, exhibited up to 93% amino acid identity in the L protein with Oak‐Vale virus (AEJ07657), an anopheles‐associated virus (marked in green dashed box in Figure S10, Supporting Information). This is the first identification of bird‐related sunrhaviruses in Asia. The Apus apus virus (SRR21777133) formed an independent clade (marked in blue dashed box in Figure S10, Supporting Information). To give a more detail topology, we reconstructed the topology of the family Rhabdoviridae with those viral species had complete genomes (Figure S28, Supporting Information). Pairwise p‐distance analysis revealed that the genetic distance between the Apus apus virus and other genera in this family ranged from 0.75 to 0.78, whereas intra‐genus distances among other genera consistently remained below 0.6. This finding suggests it may be a novel genus.

Within the family Coronaviridae, a nearly completed coronavirus was identified in a bat (Molossus molossus, marked in blue dashed box in Figure S11A, Supporting Information). It displayed 94% amino acid identity with Eptesicus bat coronavirus (UNE74440) in the RdRp protein. Phylogenetic analyses based on genome‐wide and spike protein sequences corroborated that this bat coronavirus clusters with other bat‐derived Alphacoronaviruses (e.g., Eptesicus serotinus, Tadarida brasiliensis, Eptesicus fuscus, Pipistrellus sp.), forming a monophyletic group distinct from the 15 currently recognized subgenera by the ICTV (Figure S29, Supporting Information). There are no significant recombination signals in this genome (Figure S30, Supporting Information). Additionally, the spike (S) protein sequences of the bat coronavirus lacked a furin cleavage site. Our analysis of all available coronavirus spike protein sequences identified furin cleavage sites in five avian coronavirus sequences and two feline coronavirus sequences (Figure S11B, Supporting Information). Striking, sequences of SARS‐CoV‐2 were detected in five goats and one cactus mouse (*Peromyscus eremicus*) (marked in green dashed box in Figure S11A, Supporting Information).

In the Picornaviridae family, we identified a highly diverse set of picornaviruses encompassing all currently recognized subfamilies, including 54 picornaviruses with near‐complete genomes (Figure 4 and Figure S12, Supporting Information). Phylogenetic analysis was further performed on the picornaviruses with 52 near‐complete genomes (two of the 54 near‐complete genomes had incomplete 3D polymerase protein, thus they were excluded) using the full‐length amino acid sequences of the 3D polymerase protein (Figure S31, Supporting Information). Phylogenetic analyses revealed that three of these viruses clustered with members of other families within the order Picornavirales, 36 clustered within established genera, while the remaining 13 formed seven distinct clades (marked in yellow dashed box in Figure S31, Supporting Information). In addition, a total of 14 sequences of picornaviruses, classified into four genera, including Cardiovirus, Hungarovirus, Parabovirus, and Kunsagivirus, were detected in wood mice (Apodemus sylvaticus, marked in green dashed box in Figure S31, Supporting Information). Notably, co‐infection involving both Cardiovirus and Kunsagivirus was observed in a single sample (marked in black dashed arrow in Figure S31, Supporting Information). This finding highlights the significant diversity of picornaviruses present in wood mice and suggests that this species may act as a crucial reservoir for the evolutionary diversification of picornaviruses.

In the family Polyomaviridae, sequences of betapolyomaviruses were identified in 55, 2, and 2 SRR datasets derived from three bats: *Rousettus aegyptiacus*, *Hypsignathus monstrosus*, and *Hipposideros armiger*, respectively. These sequences exhibited a close phylogenetic relationship with viruses found in pandas, goats, rodents, primates (*Chlorocebus aethiops*), and avian species (*Anser platyrhynchos* and *Taeniopygia guttata*), demonstrating an amino acid identity of 99%–100% in the large T antigen. The Junco hyemalis polyomavirus (ERR2586072, marked in blue dashed box in Figure S13, Supporting Information) exhibited 75% amino acid identity in the large T antigen with Canary polyomavirus (GU345044), which has been implicated in fatal disease outbreaks among canary birds.^[^ ^22^ ^]^

In the family Peribunyaviridae, a bunyavirus from Myotis riparius (SRR13631492) exhibited 89% amino acid identity with a tick‐derived Peribunyaviridae (WAK75613), while the other bunyavirus from Myotis lucifugus (SRR5812833) exhibited 70% amino acid identity with the other tick‐derived Peribunyaviridae (UYL95512) in the L protein. Phylogenetic analysis indicated that these bunyaviruses from Myotis bats and ticks formed a distinct clade, implying the possibility of cross‐species transmission (marked in blue dashed box in Figure S14, Supporting Information).

Within the Orthomyxoviridae family, *PB1* gene sequences identified from five goats, one bat, and one Sumatran orangutan clustered with avian influenza viruses, as indicated by the blue dashed box in Figure S18, Supporting Information. In the Paramyxoviridae family, 12, 10, 15, and 1 sequences were identified from primate species (*Pongo pygmaeus*, *Gorilla gorilla*, *Pan troglodytes*, and *Callithrix jacchus*), a rodent (*Ictidomys tridecemlineatus*), a bat (*Rhinolophus ferrumequinum*), and a duck (*Anas platyrhynchos*) respectively. These sequences exhibited a high degree of similarity, with an 85% amino acid identity to the human parainfluenza virus, as highlighted in the blue dashed box in Figure S20, Supporting Information. Additionally, sequences of parainfluenza virus 5 were detected in 19 SRR datasets from ducks and three SRR datasets from yaks (*Bos grunniens*), as denoted by the green dashed box in Figure S20A, Supporting Information. A total of seven sequences were identified as containing a putative Furin cleavage site. These included four Morbillivirus sequences detected in *Capra hircus*, one bat morbillivirus sequence derived from *Molossus molossus*, one avian paramyxovirus 1 sequence obtained from *Calidris ruficollis*, and one avian paramyxovirus 5 sequence from *Trichoglossus moluccanus* (Figure S20B, Supporting Information). In the Pneumoviridae family, sequences from three SRR datasets of yaks, one SRR dataset of goats, and five SRR datasets of cotton rats (*Sigmodon hispidus*) were found to cluster closely with the human respiratory syncytial virus, exhibiting an amino acid identity ranging from 96% to 100%, as illustrated in Figure S23, Supporting Information.

### 2.5 Novel and Expanded Virus–Host Networks

**Figure 5:**
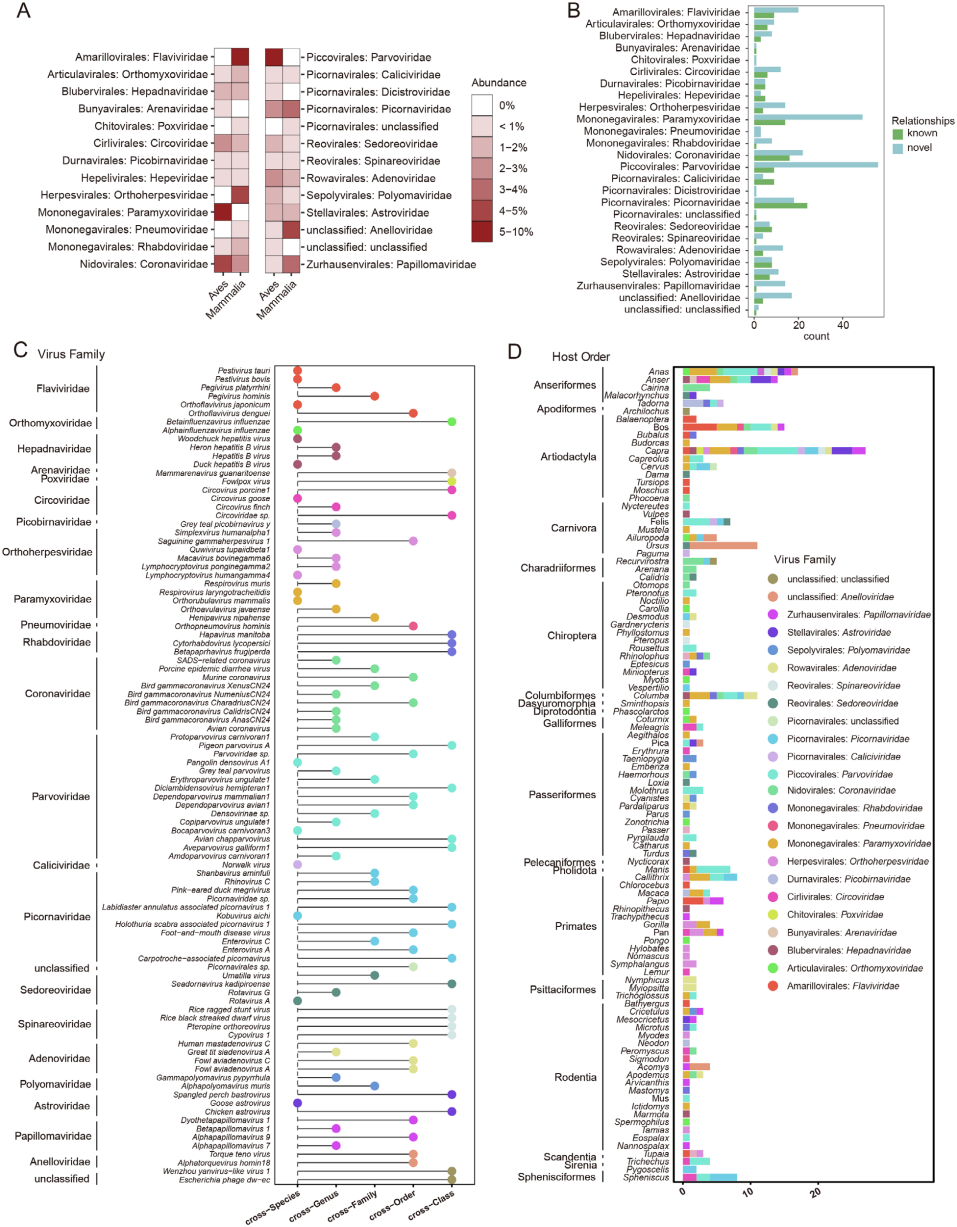
Virus–host networks. A) Quantitative compositions of newly identified virus‐host relationships across various virus families in mammals and avians. B) The quantity of virus‐host relationships across different viral orders and families. C) A comparison of the transmission dynamics of viruses with newly identified virus‐host relationships across various host species, genera, families, and orders, in relation to established virus‐host relationships. D) The number of novel virus‐host relationships categorized by viral family among different host genera.

To elucidate the viral host range and potential spillover events, the virus‐host relationships identified in this study were compared with those documented in established databases, including Kyoto Encyclopedia of Genes and Genomes (KEGG) virus‐host, CLOVER^[^ ^23^ ^]^ and manually curated records from GenBank, in order to uncover novel virus‐host associations. A total of 1592 virus‐host relationships were identified, of which 458 exhibited greater than 95% amino acid sequence similarity to known viruses and were included in subsequent analyses (Supporting Data 7). Notably, the majority (311, 67.9%) were newly identified (**Figure** **5**B). Among domestic animals, goats, yak, pigeons, and ducks exhibited a relatively high number of novel virus‐host relationships (Figure 5D). Additionally, 56 and 49 novel virus‐host relationships were identified within the Parvoviridae and Paramyxoviridae families, respectively, indicating a broader host range expansion for these viral groups than previously recognized (Figure 5B). The newly identified virus‐host relationships exhibited significant variation between avian and mammalian hosts (Chi‐squared test, *p* = 0.03), with mammals representing the predominant hosts in these newly identified associations (63.3%, 197/311) (Figure 5A). Furthermore, 26 novel viruses from the Parvoviridae and Picornaviridae families were associated with known human or domestic animal hosts, spanning multiple taxonomic levels, thereby suggesting a broader host range than previously anticipated (Figure 5C). For example, the Foot‐and‐mouth disease virus was identified for the first time in the Asian particolored bat (*Vespertilio sinensis*), while enterovirus A, which can cause asymptomatic infections in humans, was found to infect goat. The cross‐species transmitted viruses identified in this study encompassed 24 viral families, particularly those from the Parvoviridae, Picornaviridae, Coronaviridae, Flaviviridae, and Paramyxoviridae families. Notably, most cross‐species transmission events occurred at the genus or higher taxonomic level. Within the Flaviviridae family, characterized by a broad host range, all novel relationships were exclusively identified in mammals. In contrast, viruses from the Coronaviridae family exhibited a wider host spectrum, with the majority of novel virus‐host associations (14 out of 22) detected in avian species. Noteworthy findings include the identification of a SARS‐related coronavirus in the wood mouse (*Apodemus sylvaticus*) and the detection of murine coronavirus in goats.

### 2.6 Diversity and Host Distribution of Potential Zoonotic Viruses

**Figure 6:**
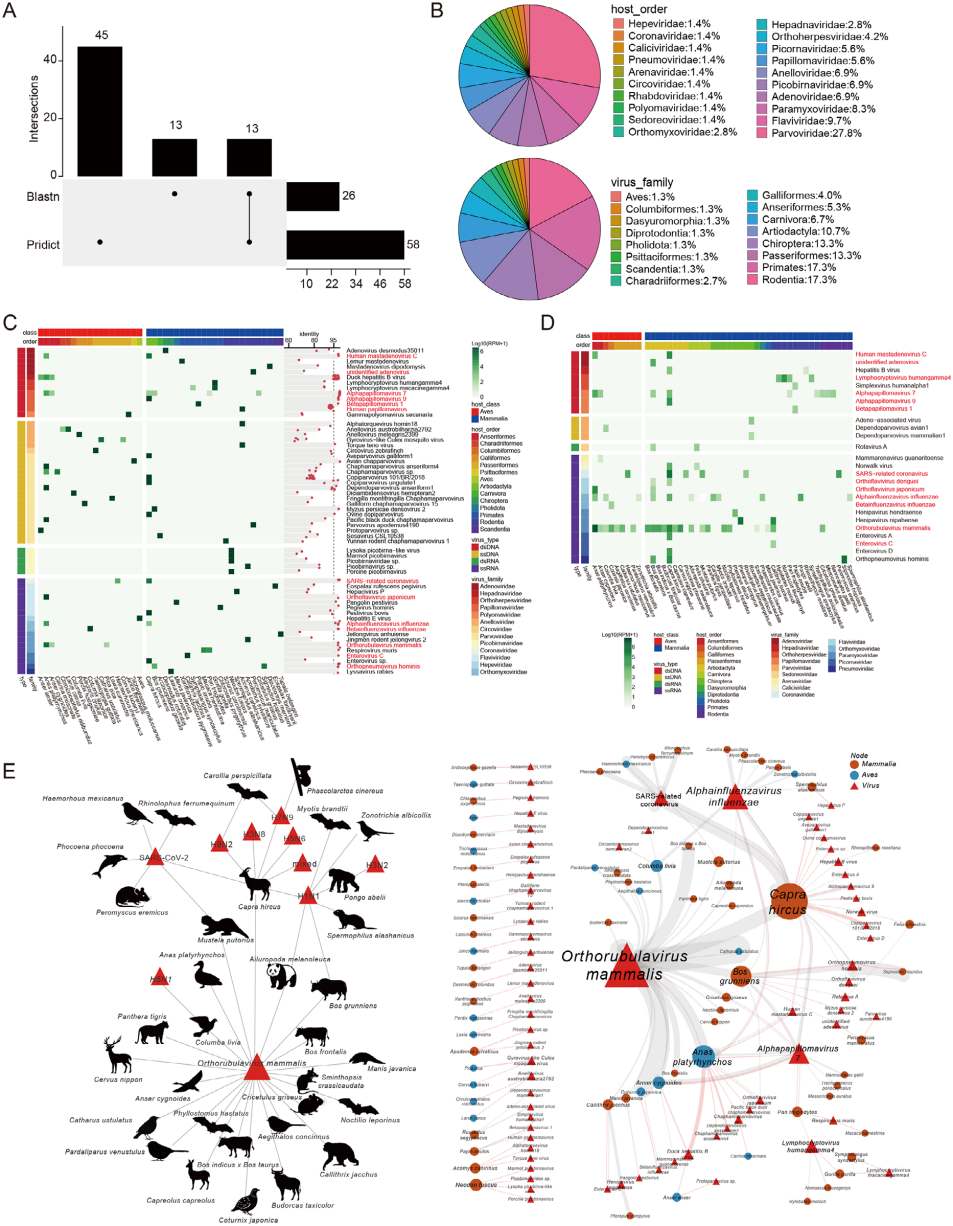
Overview of potential zoonotic viruses. A) Comparative analysis of the number of zoonotic viruses identified through BLASTn and machine learning methodologies. B) Pie chart that delineates the distribution of host orders and viral families associated with zoonotic virus‐host interactions. The heatmaps in panels (C) and (D) depict predicted zoonotic viruses identified via BLASTn and machine learning techniques, respectively. The x‐axis corresponds to host species, with color‐coded bars representing various host orders and classes, while the y‐axis indicates virus species, with color bars signifying different viral nucleic acid types and families. The values presented are log_10_(RPM+1). (E) Network of identified virus‐host interactions involving potential zoonotic viruses, where red triangles denote viral species and circles represent host species, with blue indicating Aves and brown indicating Mammalia.

In our investigation of potential zoonotic viruses, we utilized both machine learning techniques and BLASTn‐based methodologies (details in Materials and Methods), which resulted in the identification of 58 and 26 viruses, respectively, with 13 viruses being common to both approaches (**Figure** **6**A and Supporting Data 8). The viruses identified were linked to 15 distinct host orders, with Rodentia, Primates, Chiroptera, Passeriformes, and Artiodactyla representing the largest proportions, accounting for 17.3%, 17.3%, 13.3%, 13.3%, and 10.7% of the zoonotic viruses, respectively. Additionally, the Parvoviridae family constituted 27.8% of the virus‐host associations (Figure 6B). Notably, we identified the hepatitis B virus, human alphaherpesvirus 1, henipavirus, and poliovirus in non‐human primate species, including *Rhinopithecus roxellana*, *Papio anubis*, and *Callithrix jacchus* (Figure 6C,D). In bat species such as *Pteropus alecto*, *Pteropus vampyrus*, and *Lasiurus cinereus*, we detected henipavirus, hendra virus, avian influenza virus, and lyssavirus rabies (Figure 6C,D). Furthermore, mammalian orthorubulavirus 5 was observed in various avian species, including *Anas platyrhynchos*, *Anser cygnoides*, and *Catharus ustulatus*.

Parainfluenza virus 5, along with various avian influenza viruses (AIVs), as well as SARS‐CoV‐2, were identified in 137, 21, and 11 SRR datasets, respectively. They exhibited the most extensive host ranges, being identified in 23, 10, and 6 species, respectively (Figure 6E and Supporting Data 9). The H5N1 virus was detected in six SRR datasets of pigeons. The *HA* gene of H1N1/pdm09 was identified in a giant panda (*Ailuropoda melanoleuca*). The *PB1*, *PB2*, *PA*, *HA*, *NP*, and *NS* genes of H7N9 were identified in short‐tailed fruit bats (*Carollia perspicillata*). The *PB1*, *PB2*, *PA*, *NA*, *M* and *NS* genes of H7N9 were found in the SRR10530607 dataset from a goat (*Capra hircus*). The *PB1*, *PB2*, *PA*, *NP*, and *M* genes of the human H3N2 strain were identified in the SRR6026509 dataset from a Sumatran orangutan (*Pongo abelii*). Moreover, partial gene segments of AIVs were detected in several mammals, including the koala (*Phascolarctos cinereus*), Brandt's bat (*Myotis brandtii*), yak (*Bos grunniens*), and ground squirrel (*Spermophilus alashanicus*) (Figure S32A–Q, Supporting Information). Four PB2 genes derived from *Carollia perspicillata*, *Pongo abelii*, and *Capra hircus* exhibited the E627K substitution, a mutation known to be linked with adaptation to mammalian hosts (Figure S32R, Supporting Information). Furthermore, sequences of SARS‐CoV‐2 were identified in five SRRs from goats, one SRR from ferret (*Mustela putorius*), one SRR from porpoise (*Phocoena phocoena*), and one SRR from cactus mouse (*Peromyscus eremicus*). Notably, SARS‐CoV‐2 sequences were also found in two SRRs from the house finch (*Haemorhous mexicanus*) (Figure S33, Supporting Information).

Furthermore, species such as goats, yaks, and ducks were found to harbor the highest diversity of zoonotic viruses (Figure 6E). Specifically, norovirus GII, dengue virus, enterovirus A, and human respiratory syncytial virus were detected in goats, while yaks were found to carry dengue virus, Japanese encephalitis virus, and human respiratory syncytial virus.

## 3 Discussion

Wildlife is a reservoir for an extensive array of viruses, many of which are inadequately characterized, creating a considerable gap in pandemic preparedness.^[^ ^24^, ^25^, ^26^ ^]^ The increasing interaction between humans and wildlife, driven by factors such as deforestation, urbanization, wildlife farming and wildlife trade, further intensifies these risks.^[^ ^27^, ^28^, ^29^ ^]^ Consequently, systematic surveillance of viral pathogens in wildlife is crucial for the early prevention of emerging infectious diseases. The advent of HTS technologies has transformed our capacity to investigate viral diversity within wildlife populations. Nevertheless, our comprehension of the global virome remains incomplete due to limitations in species representation, sample sizes, and geographic coverage in existing research.

The extensive collection of HTS data available in public repositories, primarily generated for metagenomic investigations of antibiotic resistance genes or transcriptomic analyses of host gene expression, serves as a significant resource for the secondary identification of viral sequences. Our comprehensive data compilation includes over 57 536 SRRs derived from 2520 raw datasets (Supporting Data 1). This dataset offers an opportunity to elucidate the global viral landscape in wild mammals and birds, thereby revealing novel viral entities, patterns of viral circulation, and insights into viral phylogeography. However, it is important to note that GenBank relies heavily on direct submissions from various laboratories, which can result in the presence of erroneous sequences.^[^ ^30^, ^31^, ^32^ ^]^ The reliability of the data is paramount, as inaccuracies can lead to confusion and potentially irreproducible results. To address this issue, we developed a workflow designed to identify potentially contaminated SRRs (Figure S1, Supporting Information). Our analysis of assembled mitochondrial DNA revealed discrepancies in the deduced hosts for 169 SRRs when compared to NCBI records, indicating potential contamination (Figure 1 and Supporting Data 2). Notably, human and rodent nucleic acids constituted the primary sources of contamination, accounting for 79.9% of the identified contaminants. The manual experimental operations and the widespread use of human and rodent cells in laboratory settings may facilitate nucleic acid contamination in HTS sequencing.

After excluding the potentially contaminated data, we identified over 613.45 million contigs and detected 131 059 viral sequences, along with 30.71 million unclassified “dark matter” sequences. Our focus subsequently shifted to 25 non‐retroviral families known to infect humans, resulting in the identification of 9788 viral sequences, of which 44% are potential novel viral sequences. These findings corroborate predictions regarding the existence of numerous uncharacterized viruses within wildlife populations. The observed geographical concordance between host and viral diversity (Figure 3) supports the ecological hypothesis that “increased host diversity deepens the pathogen pool,”^[^ ^10^ ^]^ suggesting that regions with naturally high biodiversity may serve as reservoirs for emerging pathogens.^[^ ^33^ ^]^ This underscores the critical importance of viral surveillance in biodiverse regions. Furthermore, the spatial heterogeneity observed among different viral families reflects the ecological complexity of viral distribution and emphasizes the necessity for tailored, region‐specific monitoring strategies. It is important to consider that the uneven availability of data across different regions may introduce biases in the interpretation of viral diversity and distribution patterns. Environmental covariates such as climate variables, land use patterns, and habitat heterogeneity likely influence both host and viral community structures. However, the absence of precise geographical coordinates and environmental metadata in many biosample records precluded comprehensive analysis of these potentially confounding factors.

Phylogenetic analysis has elucidated the intricate evolutionary trajectories of various viruses, highlighting their expanded host ranges, instances of co‐infection, and providing novel insights into potential viral reservoirs. Notably, pestiviruses encompass significant pathogens affecting livestock, including the classical swine fever virus, atypical porcine pestivirus, bovine viral diarrhea virus, and border disease virus.^[^ ^34^, ^35^ ^]^ This study identified two novel pestiviruses in rodent populations, thereby augmenting the host range and diversity of pestiviruses (Figure S27, Supporting Information). Hepaciviruses and pegiviruses demonstrate a long‐term coevolutionary relationship with their hosts.^[^ ^36^, ^37^ ^]^ While the majority of viruses identified in this research conform to this evolutionary pattern, the hepacivirus found in the numbat (*Myrmecobius fasciatus*) represents an anomaly, as it is positioned at the base of a clade comprising birds and reptiles.^[^ ^38^ ^]^ Additionally, a hepacivirus detected in the mole (*Scalopus aquaticus*) also deviates from the norm, occupying a basal position within a rodent‐reptile clade (indicated by the yellow dashed box in Figure S27, Supporting Information). These atypical phylogenetic placements may indicate an ancient host‐switching event followed by subsequent divergence, or they may arise from limited sampling or methodological biases in phylogenetic reconstruction. Therefore, there is a pressing need for expanded sampling of underrepresented vertebrate hepaciviruses and more comprehensive phylogenomic analyses to elucidate their interrelationships. Historically, co‐infections involving hepacivirus and pegivirus have been documented solely in bats and rodents, which are posited to be natural reservoirs for these viruses.^[^ ^25^, ^39^, ^40^ ^]^ This study, however, reports the co‐infection of these viruses in tree shrews, suggesting that this species may serve as a potential reservoir for hepaciviruses and emphasizes their value as experimental models for hepatitis virus infection research.^[^ ^41^ ^]^ Furthermore, co‐infections of Cardiovirus, Hungarovirus, Parabovirus, and Kunsagivirus were identified in *Apodemus sylvaticus*, indicating that this rodent may also act as a potential reservoir for picornaviruses (marked in the green dashed box in Figure S31, Supporting Information). Although no significant recombination events were observed in these co‐infections, the presence of multiple viral infections may facilitate genetic rearrangements or recombination, thereby heightening the risk of emerging pathogens—a phenomenon that has been previously documented in rodent populations.^[^ ^40^ ^]^

In addition to the emergence of novel viruses, cross‐species viral transmissions have been observed to be widespread, with 67.9% of identified viruses detected in multiple species across various taxonomic classifications, including species, genera, family, and even class. Certain instances of viral cross‐infection may be indicative of predator‐prey dynamics. For instance, the parvovirus associated with voles and wood mice exhibited a clustering pattern with canine parvoviruses, as illustrated within the red dashed box in Figure S6, Supporting Information. Additionally, circoviruses derived from opossums, rodents and bats clustered with the felid circovirus (marked in yellow dashed box in Figure S8, Supporting Information). Notably, new virus‐host associations were predominantly identified in domesticated animals, particularly in goats and yaks (Figure 5D). Among the 30 recognized zoonotic viruses, an additional 48 potential zoonotic viruses were predicted, with goats and yaks exhibiting the highest prevalence of zoonotic viruses. A nearly complete genome of H7N9, along with partial gene segments of H1N1, H3N8, H5N6, and H9N2, has been detected in goats. This discovery highlights the imperative for additional research to elucidate the role of goats in the transmission dynamics of avian influenza viruses. Domestic animals serve as a significant conduit for the transmission of viruses from wildlife to humans.^[^ ^42^ ^]^ The intensification of domestic animal husbandry, coupled with changes in land use, has increased interactions between domestic animals and wildlife.^[^ ^24^, ^43^ ^]^ This situation underscores the necessity for improved surveillance at the interface between wildlife and domestic animals.

In addition to the identification of novel viruses and novel virus‐host interactions, we discovered 137, 21, and 11 SRR datasets that were not originally designed for viral research but contained sequences of parainfluenza virus 5, avian influenza viruses, and SARS‐CoV‐2, respectively (Figure 6E and Supporting Data 9). This observation indicates that a substantial amount of viral data, which has previously been overlooked, remains untapped within public HTS databases. Sequences of SARS‐CoV‐2 were identified in datasets of goats, ferrets, porpoises, cactus mice, and house finches (Figure S33, Supporting Information and Supporting Data 9). Although these datasets pertaining to SARS‐CoV‐2 were published after the COVID‐19 pandemic, they provide valuable insights as early indicators of viral transmission and the expansion of host range.

Viral contigs obtained from HTS frequently remain unsubmitted to the NCBI. This lack of submission necessitates the redundant reassembly of data in subsequent studies conducted by various research groups, leading to significant computational inefficiencies and severely constraining the identification of novel viruses, which relies heavily on sequence alignment with NT or NR databases of NCBI. Furthermore, the so‐called “dark matter” derived from metagenomic sequences, which do not align with any reference viral sequences, constitutes ≈40–90% of total sequences.^[^ ^44^ ^]^ These sequences may represent a multitude of novel microbes and proteins.^[^ ^45^, ^46^, ^47^ ^]^ Consequently, they hold significant potential for future studies, as they could be utilized to retrospectively investigate whether such viruses were present in earlier HTS datasets. To address this issue, we have developed a user‐friendly web server and interface, the Animal Pathogen Decoding Platform (https://apd.scau.edu.cn), which hosts viral contigs and unaligned “dark matter.” These initiatives not only facilitate the discovery of novel viruses and their interactions with hosts but also provide pre‐assembled viral contigs to streamline subsequent research, thereby enhancing data utility and minimizing computational resource expenditure.

In conclusion, the investigation of viruses in wildlife is crucial for the prediction and prevention of future zoonotic pandemics. By analyzing publicly available HTS datasets, this study uncovers an extensive range of viral diversity and previously unrecognized host associations. Wild mammals and birds harbor a significant variety of novel viruses, while domestic animals exhibiting the highest prevalence of zoonotic viruses and new virus‐host associations. These observations highlight the urgent need for surveillance at the domestic animals‐wildlife interface. Our research indicates that the extensive publicly accessible HTS datasets, which are not specifically focused on viromes, hold significant value for viral monitoring. Furthermore, the development of the Animal Pathogen Decoding Platform addresses a critical limitation in viral discovery by providing assembled contigs from HTS datasets.

## 4 Experimental Section

### Collection of HTS Data and Assembly of Contigs

Metadata files of wildlife were retrieved from the NCBI FTP site (https://ftp.ncbi.nlm.nih.gov/sra/reports/Metadata/) on March 31, 2025.^[^ ^48^ ^]^ The dataset comprised 43 678 mammals (370 genera and 633 species) and 13 858 birds (436 genera and 564 species), collected based on the following criteria: (1) RNA‐seq data were filtered utilizing a custom script that focused on the keyword “LIBRARY_STRATEGY”; (2) Host lineages were delineated using TaxonKit, based on the keyword “host_taxid,” which further refined the dataset to include only mammalian and avian hosts;^[^ ^49^ ^]^ (3) Data pertaining to domestic animals were excluded as outlined by Wells et al.,^[^ ^50^ ^]^ which included *Gallus gallus*, *Chlorocebus sabaeus*, *Macaca mulatta*, *Macaca fascicularis*, *Bos javanicus*, *Bos mutus*, *Bos taurus*, *Bubalus bubalis*, *Camelus bactrianus*, *Camelus dromedarius*, *Canis familiaris*, *Capra aegagrus*, *Cavia porcellus*, *Equus africanus*, *Equus asinus*, *Equus caballus*, *Felis catus*, *Lama guanicoe*, *Mus musculus*, *Oryctolagus cuniculus*, *Ovis aries*, *Rattus norvegicus*, *Rattus rattus*, *Sus scrofa*, *Vicugna vicugna*, *Camelus ferus* and *Canis lupus*. The metadata were subsequently downloaded and converted to FASTQ format utilizing the prefetch and fasterq‐dump tools from the NCBI SRA Toolkit. A preliminary quality control assessment of the sequencing data was conducted using fastp.^[^ ^51^ ^]^ The trimmed reads that met the quality control standards were mapped to the rRNA database and the genomes of the host or closely related species using RapMap and Minimap2,^[^ ^52^, ^53^ ^]^ while unmapped non‐host reads were extracted using SAMtools.^[^ ^54^ ^]^ The non‐host reads were subsequently assembled into contigs using MEGAHIT with the default parameters.^[^ ^55^ ^]^

### Host Contamination Identification in HTS Dataset

To identify potential contamination within HTS datasets, we developed a systematic workflow, as depicted in Figure S1, Supporting Information. Initially, we compiled all available complete mitochondrial genomes from the animal kingdom sourced from the NCBI genome database. Subsequently, for each species, a single representative mitochondrial genome was selected to serve as the reference, culminating in a dataset comprising mitochondrial genomes from 16 791 species.

The clean reads from each HTS dataset were aligned to the mitochondrial genome dataset utilizing Minimap2 with the command “minimap2 ‐x sr.”^[^ ^52^ ^]^ A similarity threshold of 0.65 and a coverage threshold of 0.1 were established to filter out low‐quality alignment results. Furthermore, a contamination threshold of 0.1 was implemented; this criterion stipulates that if the reads aligned to a suspected contaminated species, which is inconsistent with the documented species, constitute 10% or more of the total reads aligned across all mitochondrial genomes, that species will be classified as a potential contaminant and will proceed to the subsequent confirmation phase.

In instances where the suspected contaminated species belongs to the same genus as the documented species, we mapped the clean reads to the complete reference mitochondrial genomes of the suspected contaminated species to generate assembled mitochondrial sequences. Additional mitochondrial sequences from other species within the same genus, including the documented species, were collected, and their pairwise genetic distances were calculated. A threshold of 0.07 was established, indicating that species exhibiting a genetic distance to the suspected species of ≤0.07 cannot be distinctly differentiated from the suspected species due to their high genetic similarity. If the documented species identified in the SRR dataset falls within this category, it is inferred that the reads mapping to the suspected species may be attributed to genetic similarity rather than contamination.

In the course of our statistical analysis of the results, we encountered several issues pertaining to the potential contaminated SRR datasets. Specifically, some datasets were derived from mixed‐species sequencing yet were annotated with only a single species designation, or the species names provided were not in accordance with standardized Latin nomenclature. As a result, the suspected species, as referenced in the mitochondrial database that utilizes standardized Latin names, did not correspond with the documented species. To address this problem, we undertook a manual verification of all SRRs identified as possible contaminants within the aforementioned pipeline, scrutinizing the relevant literature for necessary corrections.

Following this verification process, we conducted BLASTn alignments of the assembled consensus sequences against the NT database. For each consensus sequence, if the highest similarity match in the NT database achieved a threshold of ≥95%, the species of the consensus was assigned to the corresponding matched species. We further assessed whether the alignment results included the documented species associated with the SRR and recorded the relevant similarity score. If the difference in similarity between the suspected species (the highest‐similarity match) and the documented species was ≤5%, the contamination was classified as a false positive due to inadequate taxonomic resolution. Conversely, if the difference exceeded this threshold, the consensus was confirmed to be a result of genuine contamination.

### Identification of Viral Sequences and Estimation of their Abundance

The assembled contigs were subjected to comparative analysis against a custom database utilizing DIAMOND, with an E‐value threshold set at 10^−^⁵ to facilitate a preliminary classification of the sequences by kingdom. This custom database was constructed from viral, bacterial, and parasitic proteins sourced from the nonredundant (NR) protein database.^[^ ^56^ ^]^ TaxonKit was employed to delineate the taxonomic lineage of the top hits, followed by the filtration of candidate viral contigs categorized under the kingdom “Viruses.”^[^ ^49^ ^]^ Contigs that did not yield matches in DIAMOND were further analyzed through rapid nucleotide searches using Kraken2, leveraging the prebuilt nucleotide (NT) database and applying a threshold of 0.1.^[^ ^57^ ^]^ Subsequently, KrakenTools was utilized to filter both candidate viral contigs and unclassified contigs.^[^ ^58^ ^]^ All candidate viral contigs were compared against the complete NR and NT databases using DIAMOND and BLASTn, with an E‐value cutoff of 10^−^⁵ and a minimum query coverage of 20% to eliminate non‐viral sequences.^[^ ^56^, ^59^ ^]^ Taxonomic lineage information was extracted based on the best hits using TaxonKit,^[^ ^49^ ^]^ and vertebrate virus sequences were filtered according to host information obtained from the Virus Metadata Resource (https://ictv.global/vmr).

To estimate the relative abundance of viral sequences, trimmed reads were initially mapped to the SILVA database to exclude ribosomal RNA reads. The remaining non‐rRNA reads were utilized to compute the abundance of the assembled contigs using Salmon.^[^ ^60^ ^]^ The viral RPM values were calculated using custom scripts, with a threshold of RPM > 3 applied to mitigate false positives.^[^ ^61^, ^62^ ^]^ To address potential false positives arising from index‐hopping, read counts of the same viral species across libraries within the same bioproject were compared. Viral contigs with read counts below 0.1% of the most abundant viral reads were excluded.^[^ ^61^ ^]^ Subsequently, viral infections were identified using a 20% alignment coverage threshold relative to the reference viral genome, a criterion established by Cline et al. through experimental viral infection studies.^[^ ^13^ ^]^ Data from experimentally inoculated viral infections were excluded through a manual review of Bioproject titles and descriptions. The subsequent analyses concentrated on 25 viral families known to infect humans, with the exception of Retroviridae.^[^ ^25^ ^]^ Retroviridae were excluded from the analysis due to the absence of high‐quality reference genomes for numerous wildlife species, which hinders the precise differentiation between endogenous retroviral sequences and exogenous retroviral infections. Finally, potential contaminant viral sequences from high‐throughput sequencing were eliminated based on the criteria outlined by Asplund et al.^[^ ^32^ ^]^

Viral contigs exhibiting less than 90% amino acid identity to known sequences were classified as novel viral sequences. Clean reads were aligned to viral contigs using Bowtie2,^[^ ^63^ ^]^ and the quality of the assembly was manually assessed using Geneious.^[^ ^64^ ^]^ The viral genomes were finalized through multiple iterations of manual genome sequence correction, read alignment, and visualization, particularly when the contigs could be manually extended at their termini. Complete open reading frames (ORFs) were identified utilizing the Prodigal software.^[^ ^65^ ^]^ The predicted ORFs underwent annotation through DIAMOND searches conducted against the NR protein database.^[^ ^56^ ^]^

### Analysis of Viral Phylogeny

The protein‐coding sequences utilized for the construction of the phylogenetic tree were selected in accordance with the guidelines established by ICTV (https://ictv.global/). The specific genes employed for tree construction included: Adenoviridae (DNA‐dependent DNA polymerase), Flaviviridae (*NS5B*), Picornaviridae (3D polymerase), Paramyxoviridae (L), Parvoviridae (*NS1*), Anelloviridae (torquevirus, *ORF1*; gyrovirus, *VP1*), Picobirnaviridae (*RdRp*), Hepadnaviridae (polymerase), Coronaviridae (*RdRp*), Orthomyxoviridae (*PB1*), Astroviridae (capsid), Polyomaviridae (LT‐Ag), Rhabdoviridae (*L*), Pneumoviridae (*L*), Circoviridae (*Rep*), Caliciviridae (capsid), Poxviridae (25 conserved tandem genes: A18, A2, A22, A23, A24, A28, A3, A32, A7, D1, D11, D12, D5, D6, E1, E10, E9, G5, H2, H4, H6, I8, J3, J6, L1), Peribunyaviridae (*L*), and Reovirales (including Sedoreoviridae, Spinareoviridae, and unclassified Reovirales, *RdRp*). Furthermore, due to the limited availability of sequences for the ICTV‐recommended marker genes, alternative genes were employed for the phylogenetic analysis of the following viral families: *L1* gene for Papillomaviridae, *RdRP* for Hepeviridae and Togaviridae, and the DNA polymerase catalytic subunit for Orthoherpesviridae. The amino acid sequences of the protein‐coding sequences for these genes were aligned using MAFFT and Clustalo.^[^ ^66^, ^67^ ^]^ Phylogenetic trees were generated using IQ‐TREE, which identified the optimal model of amino acid substitution through 1000 nonparametric bootstrap replicates.^[^ ^68^ ^]^ The resulting phylogenetic trees were annotated and visualized with Figtree. Silhouettes of animals were sourced from PhyloPic (http://www.phylopic.org) and Freepik (www.flaticon.com).

### Analysis of Novel Virus‐Host Relationships

In order to identify novel virus‐host relationships of the viruses, the Virus‐Host database within the KEGG, a synthesis of host‐virus datasets provided by Rory et al.^[^ ^69^ ^]^ and records from GenBank were manually curated and integrated. “Novel virus‐host relationships” are defined as those associations in which the viral sequence has not been previously documented in the aforementioned dataset. To ensure a rigorous methodology for analyzing the connections between viruses and their hosts while minimizing taxonomic inaccuracies, the NCBI taxonomic index and the aforementioned dataset were employed to standardize the classification of both viruses and their respective hosts. In certain instances, viruses may demonstrate a broad host range, resulting in one‐to‐many virus‐host associations. To assess the potential for cross‐taxon transmission, each novel host was compared to its most closely related known host based on taxonomic proximity. The analysis of novel relationships was conducted using the igraph package (version 2.0.3),^[^ ^70^ ^]^ and all visualizations were generated using the ggplot package (version 3.5.0).^[^ ^71^ ^]^

### Identification of Potential Zoonotic Viruses

Reference sequences of established zoonotic viruses were obtained from the existing literature.^[^ ^72^, ^73^ ^]^ The viral sequences identified in this study were compared to these reference sequences utilizing BLASTn, with hits exhibiting ≥95% identity and ≥20% coverage being classified as zoonotic virus sequences. In order to identify previously unrecognized potential zoonotic viruses, a machine learning model developed by Mollentze et al. was employed on our dataset.^[^ ^74^ ^]^ We utilized the highest prediction threshold level, designated as “Very high” to screen for viruses with a significant likelihood of zoonotic potential. The virus‐host network was constructed using Cytoscape.^[^ ^75^ ^]^

### Geographic Distribution of Viral Diversity

Geospatial data pertaining to wildlife, spanning the years 2001 to 2024, were obtained from the Global Biodiversity Information Facility (GBIF) (https://www.gbif.org/). Records that were deficient in critical information, including latitude, longitude, country, or species designation, as well as any duplicate entries, were systematically excluded from the analysis. The geographic origin (country) of each SRR was attained from the BioSample metadata, and manually curated in instances where the location information was either absent or unclear. The global distribution of wildlife and associated viruses was illustrated utilizing the R packages ggplot2 and rnaturalearth.^[^ ^71^ ^]^ Graphical representations of the spatial distribution of viruses were constructed utilizing RPM (Reads Per Million) values for each viral family across various countries or regions. RPM is defined as the number of viral reads normalized to the total number of sequencing reads. The aggregate of RPM values for all viral families was employed to quantify the overall viral abundance within each region. These data were subsequently utilized to create a variety of visualizations, including pie charts that depict the relative and total abundance of viral families by country or region, stacked bar charts that facilitate comparisons of viral composition across different geographic areas, and ring charts that highlight the proportion of viral families associated with potential zoonotic risks. For each viral family, the detection rate was determined by dividing the number of SRA runs containing viral sequences by the total number of SRA runs analyzed within the respective country. Subsequently, these rates were normalized on a per‐country basis. To assess the correlation between the number of viral families or species and wildlife diversity, we conducted Spearman rank correlation analyses. Viral diversity was quantified based on richness, defined as the count of viral families and species. To maintain statistical robustness, the analysis was restricted to countries with more than 10 virus‐positive SRRs. Each country was subjected to eight repeated samplings (n = 8) to assess the stability of the results. For each analysis, Spearman's correlation coefficient (ρ) and associated p‐values were calculated, and regression curves were fitted to depict the observed associations. The relationships were visualized through scatter plots incorporating regression lines and 95% confidence intervals, thereby illustrating both the strength and uncertainty of the correlations. All statistical analyses and graphical representations were performed using Python version 3.9, utilizing the scipy, sklearn, pandas, and matplotlib libraries.

## Conflict of Interest

The authors declare no conflict of interest.;

## Supporting information



Supporting Information

Supporting Information

## Data Availability

The data that support the findings of this study are available in the supplementary material of this article.;
