# Supplementary material for: Unveiling Novel Viral Diversity, Biogeography, and Host Networks in Wildlife Through High‐Throughput Sequencing Data Mining
Source: Adv Sci (Weinh). 2025 Sep 23;12(46):e11920. doi: 10.1002/advs.202511920 (PMC12697861; doi:10.1002/advs.202511920)
Supplement: Supplementary file 1 — Supporting Information [file ADVS-12-e11920-s001.pdf]

Fig S1

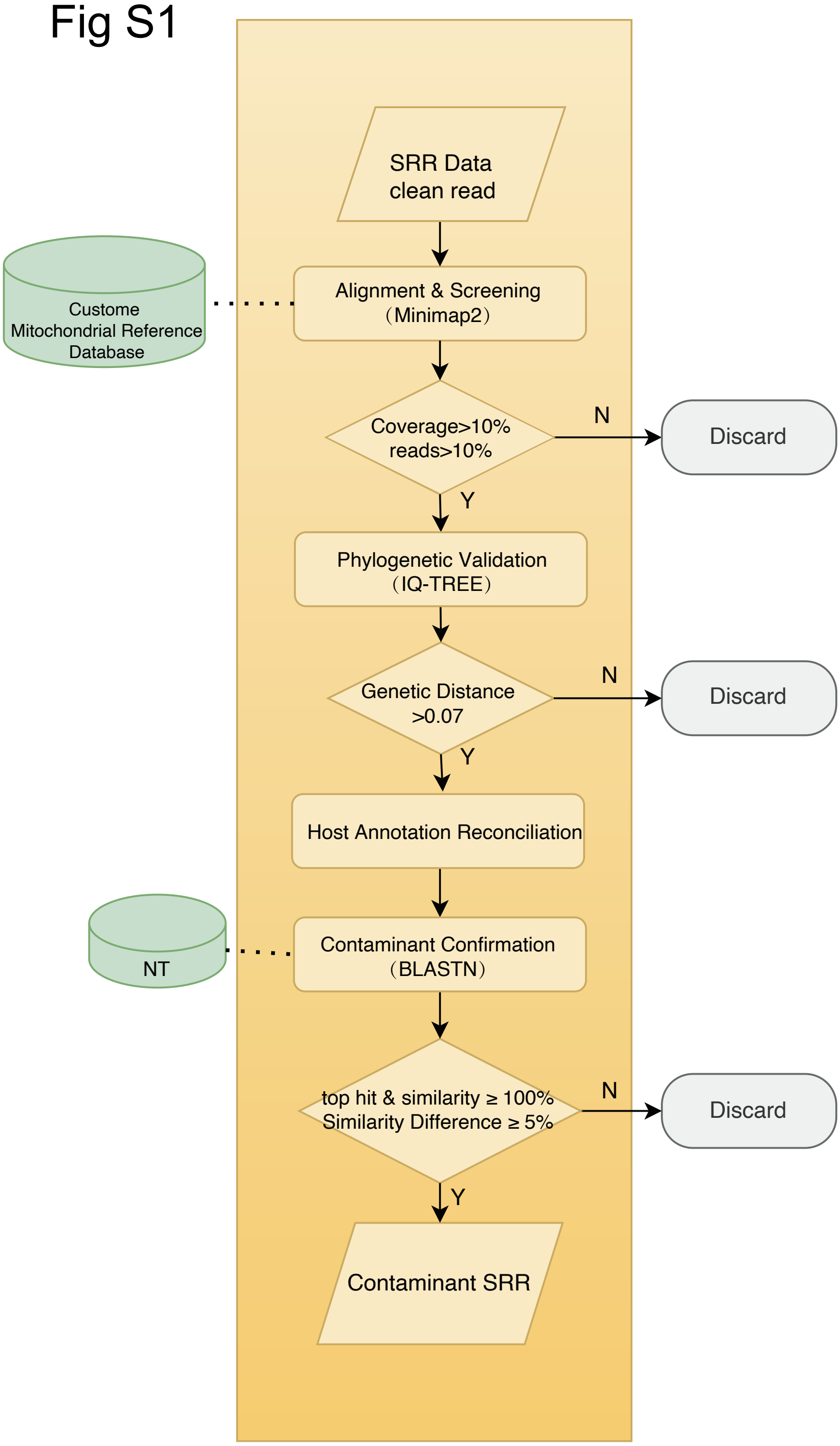
SRR Data
clean read
Custome
Mitochondrial Reference
Database
Alignment & Screening
（Minimap2）
Coverage>10%
reads>10%
N
Discard
Y
Phylogenetic Validation
（IQ-TREE）
Genetic Distance
>0.07
N
Discard
Y
Host Annotation Reconciliation
NT
Contaminant Confirmation
（BLASTN）
top hit & similarity ≥ 100%
Similarity Difference ≥ 5%
N
Discard
Y
Contaminant SRR

Fig S2

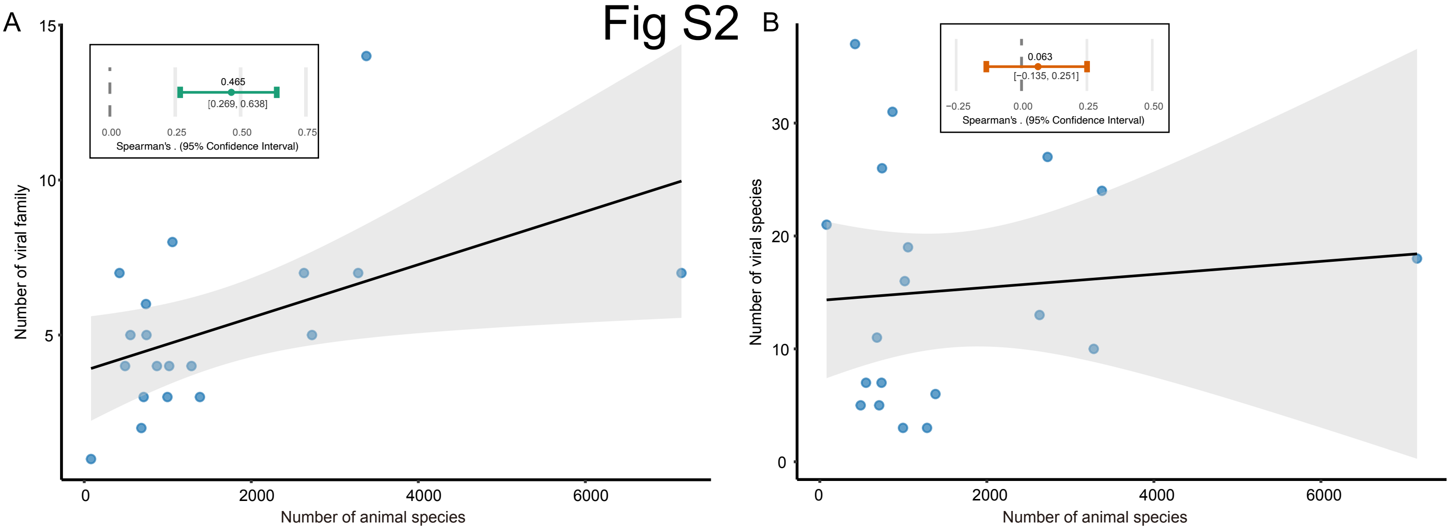

A
B
0.465
[0.269, 0.638]
0.00
0.25
0.50
0.75
Spearman's . (95% Confidence Interval)
Number of viral family
15
10
5
Number of animal species
0
2000
4000
6000
0.063
[−0.135, 0.251]
−0.25
0.00
0.25
0.50
Spearman's . (95% Confidence Interval)
Number of viral species
30
20
10
0
Number of animal species

Fig S3

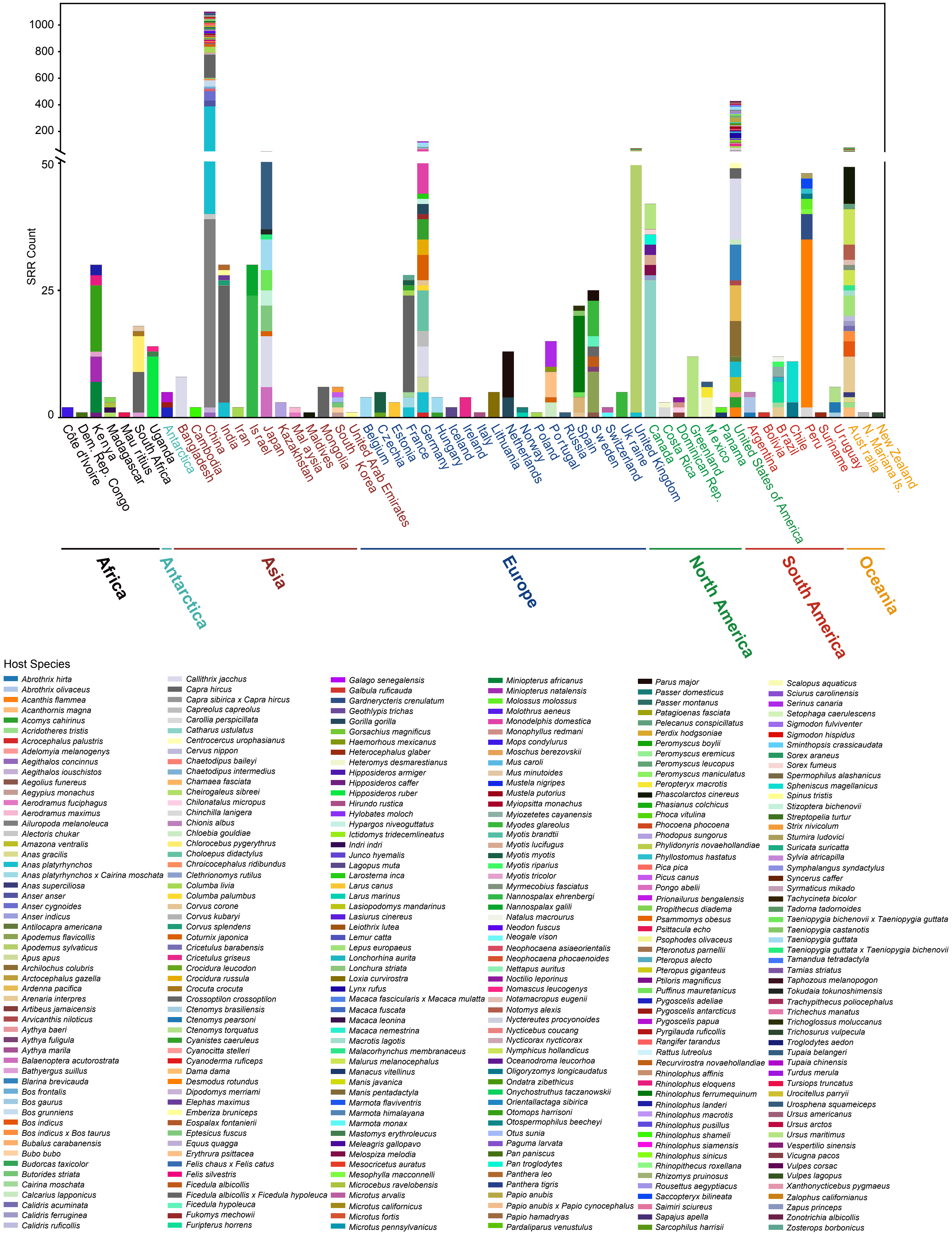

SRR Count
1000
800
600
400
200
50
25
0
Côte d'Ivoire
Dem. Rep. Congo
Kenya
Madagascar
Mauritius
South Africa
Uganda
Antarctica
Bangladesh
Cambodia
China
India
Iran
Israel
Japan
Kazakhstan
Malaysia
Maldives
Mongolia
South Korea
United Arab Emirates
Belgium
Czechia
Estonia
France
Germany
Hungary
Iceland
Ireland
Italy
Lithuania
Netherlands
Norway
Poland
Portugal
Russia
Spain
Sweden
Switzerland
Ukraine
United Kingdom
Canada
Costa Rica
Dominican Rep.
Greenland
Mexico
Panama
United States of America
Argentina
Bolivia
Brazil
Chile
Peru
Suriname
Uruguay
Australia
N. Mariana Is.
New Zealand
Africa
Antarctica
Asia
Europe
North America
South America
Oceania
Host Species
Abrothrix hirta
Abrothrix olivaceus
Acanthis flammea
Acanthornis magna
Acomys cahirinus
Acridotheres tristis
Acrocephalus palustris
Adelomyia melanogenys
Aegithalos concinnus
Aegithalos iouschistos
Aegolius funereus
Aegypius monachus
Aerodramus fuciphagus
Aerodramus maximus
Ailuropoda melanoleuca
Alectoris chukar
Amazona ventralis
Anas gracilis
Anas platyrhynchos
Anas platyrhynchos x Cairina moschata
Anas superciliosa
Anser anser
Anser cygnoides
Anser indicus
Antilocapra americana
Apodemus flavicollis
Apodemus sylvaticus
Apus apus
Archilochus colubris
Arctocephalus gazella
Ardenna pacifica
Arenaria interpres
Artibeus jamaicensis
Arvicanthis niloticus
Aythya baeri
Aythya fuligula
Aythya marila
Balaenoptera acutorostrata
Bathyergus suillus
Blarina brevicauda
Bos frontalis
Bos gaurus
Bos grunniens
Bos indicus
Bos indicus x Bos taurus
Bubalus carabanensis
Bubo bubo
Budorcas taxicolor
Butorides striata
Cairina moschata
Calcarius lapponicus
Calidris acuminata
Calidris ferruginea
Calidris ruficollis
Callithrix jacchus
Capra hircus
Capra sibirica x Capra hircus
Capreolus capreolus
Carollia perspicillata
Catharus ustulatus
Centrocercus urophasianus
Cervus nippon
Chaetodipus baileyi
Chaetodipus intermedius
Chamaea fasciata
Cheirogaleus sibreei
Chilonatalus micropus
Chinchilla lanigera
Chionis albus
Chloebia gouldiae
Chlorocebus pygerythrus
Choloepus didactylus
Chroicocephalus ridibundus
Clethrionomys rutilus
Columba livia
Columba palumbus
Corvus corone
Corvus kubaryi
Corvus splendens
Coturnix japonica
Cricetulus barabensis
Cricetulus griseus
Crocidura leucodon
Crocidura russula
Crocuta crocuta
Crossoptilon crossoptilon
Ctenomys brasiliensis
Ctenomys pearsoni
Ctenomys torquatus
Cyanistes caeruleus
Cyanocitta stelleri
Cyanoderma ruficeps
Dama dama
Desmodus rotundus
Dipodomys merriami
Elephas maximus
Emberiza bruniceps
Eospalax fontanierii
Eptesicus fuscus
Equus quagga
Erythrura psittacea
Felis chaus x Felis catus
Felis silvestris
Ficedula albicollis
Ficedula albicollis x Ficedula hypoleuca
Ficedula hypoleuca
Fukomys mechowii
Furipterus horrens
Galago senegalensis
Galbula ruficauda
Gardnerycteris crenulatum
Geothlypis trichas
Gorilla gorilla
Gorsachius magnificus
Haemorhous mexicanus
Heterocephalus glaber
Heteromys desmarestianus
Hipposideros armiger
Hipposideros caffer
Hipposideros ruber
Hirundo rustica
Hylobates moloch
Hypargos niveoguttatus
Ictidomys tridecemlineatus
Indri indri
Junco hyemalis
Lagopus muta
Larosterna inca
Larus canus
Larus marinus
Lasiopodomys mandarinus
Lasiurus cinereus
Leiothrix lutea
Lemur catta
Lepus europaeus
Lonchorhina aurita
Lonchura striata
Loxia curvirostra
Lynx rufus
Macaca fascicularis x Macaca mulatta
Macaca fuscata
Macaca leonina
Macaca nemestrina
Macrotis lagotis
Malacorhynchus membranaceus
Malurus melanocephalus
Manacus vitellinus
Manis javanica
Manis pentadactyla
Marmota flaviventris
Marmota himalayana
Marmota monax
Mastomys erythroleucus
Meleagris gallopavo
Melospiza melodia
Mesocricetus auratus
Mesophylla macconnelli
Microcebus ravelobensis
Microtus arvalis
Microtus californicus
Microtus fortis
Microtus pennsylvanicus
Miniopterus africanus
Miniopterus natalensis
Molossus molossus
Molothrus aeneus
Monodelphis domestica
Monophyllus redmani
Mops condylurus
Moschus berezovskii
Mus caroli
Mus minutoides
Mustela nigripes
Mustela putorius
Myiopsitta monachus
Myiozetetes cayanensis
Myodes glareolus
Myotis brandtii
Myotis lucifugus
Myotis myotis
Myotis riparius
Myotis tricolor
Myrmecobius fasciatus
Nannospalax ehrenbergi
Nannospalax galili
Natalus macrourus
Neodon fuscus
Neogale vison
Neophocaena asiaeorientalis
Neophocaena phocaenoides
Nettapus auritus
Noctilio leporinus
Nomascus leucogenys
Notamacropus eugenii
Notomys alexis
Nyctereutes procyonoides
Nycticebus coucang
Nycticorax nycticorax
Nymphicus hollandicus
Oceanodroma leucorhoa
Oligoryzomys longicaudatus
Ondatra zibethicus
Onychostruthus taczanowskii
Orientallactaga sibirica
Otomops harrisoni
Otospermophilus beecheyi
Otus sunia
Paguma larvata
Pan paniscus
Pan troglodytes
Panthera leo
Panthera tigris
Papio anubis
Papio anubis x Papio cynocephalus
Papio hamadryas
Pardaliparus venustulus
Parus major
Passer domesticus
Passer montanus
Patagioenas fasciata
Pelecanus conspicillatus
Perdix hodgsoniae
Peromyscus boylii
Peromyscus eremicus
Peromyscus leucopus
Peromyscus maniculatus
Peropteryx macrotis
Phascolarctos cinereus
Phasianus colchicus
Phocoena phocoena
Phoca vitulina
Phodopus sungorus
Phylidonyris novaehollandiae
Phyllostomus hastatus
Pica pica
Picus canus
Pongo abelii
Prionailurus bengalensis
Propithecus diadema
Psammomys obesus
Psittacula echo
Psophodes olivaceus
Pteronotus parnellii
Pteropus alecto
Pteropus giganteus
Ptiloris magnificus
Puffinus mauretanicus
Pygoscelis adeliae
Pygoscelis antarcticus
Pygoscelis papua
Pyrgilauda ruficollis
Rangifer tarandus
Rattus lutreolus
Recurvirostra novaehollandiae
Rhinolophus affinis
Rhinolophus eloquens
Rhinolophus ferrumequinum
Rhinolophus landeri
Rhinolophus macrotis
Rhinolophus pusillus
Rhinolophus shameli
Rhinolophus siamensis
Rhinolophus sinicus
Rhinopithecus roxellana
Rhizomys pruinosus
Rousettus aegyptiacus
Saccopteryx bilineata
Saimiri sciureus
Sapajus apella
Sarcophilus harrisii
Scalopus aquaticus
Sciurus carolinensis
Serinus canaria
Setophaga caerulescens
Sigmodon fulviventer
Sigmodon hispidus
Sminthopsis crassicaudata
Sorex araneus
Sorex fumeus
Spermophilus alashanicus
Spheniscus magellanicus
Spinus tristis
Stizoptera bichenovii
Streptopelia turtur
Strix nivicolum
Sturnira ludovici
Suricata suricatta
Sylvia atricapilla
Symphalangus syndactylus
Syncerus caffer
Syrmaticus mikado
Tachycineta bicolor
Tadorna tadornoides
Taeniopygia bichenovii x Taeniopygia guttata
Taeniopygia castanotis
Taeniopygia guttata
Taeniopygia guttata x Taeniopygia bichenovii
Tamandua tetradactyla
Tamias striatus
Taphozous melanopogon
Tokudaia tokunoshimensis
Trachypithecus poliocephalus
Trichechus manatus
Trichoglossus moluccanus
Trichosurus vulpecula
Troglodytes aedon
Tupaia belangeri
Tupaia chinensis
Turdus merula
Tursiops truncatus
Urocitellus parryii
Urosphena squameiceps
Ursus americanus
Ursus arctos
Ursus maritimus
Vespertilio sinensis
Vicugna pacos
Vulpes corsac
Vulpes lagopus
Xanthonycticebus pygmaeus
Zalophus californianus
Zapus princeps
Zonotrichia albicollis
Zosterops borbonicus

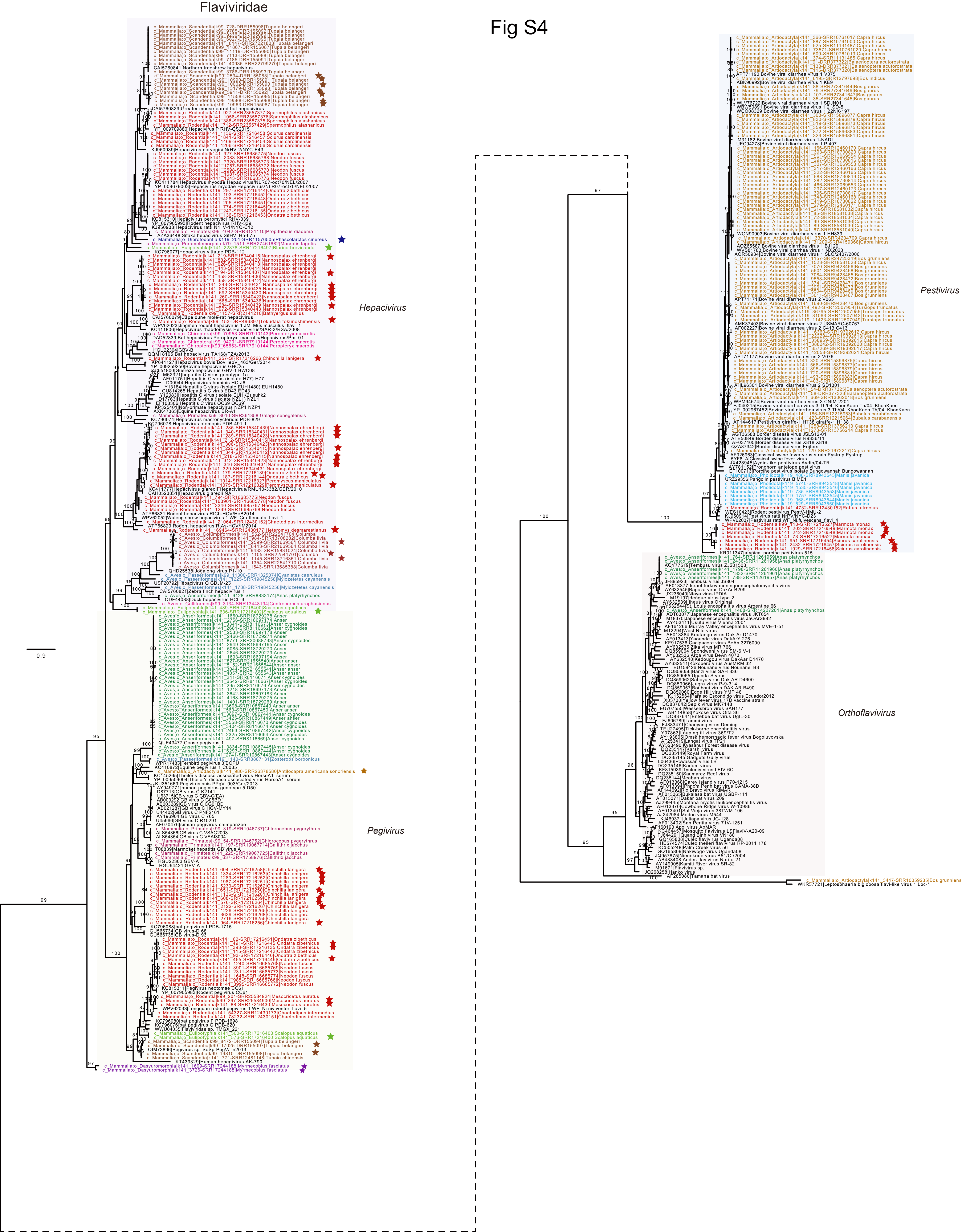
Flaviviridae
Hepacivirus
Pegivirus
Pestivirus
Orthoflavivirus
0.9

Fig S4

Fig S5

Astroviridae

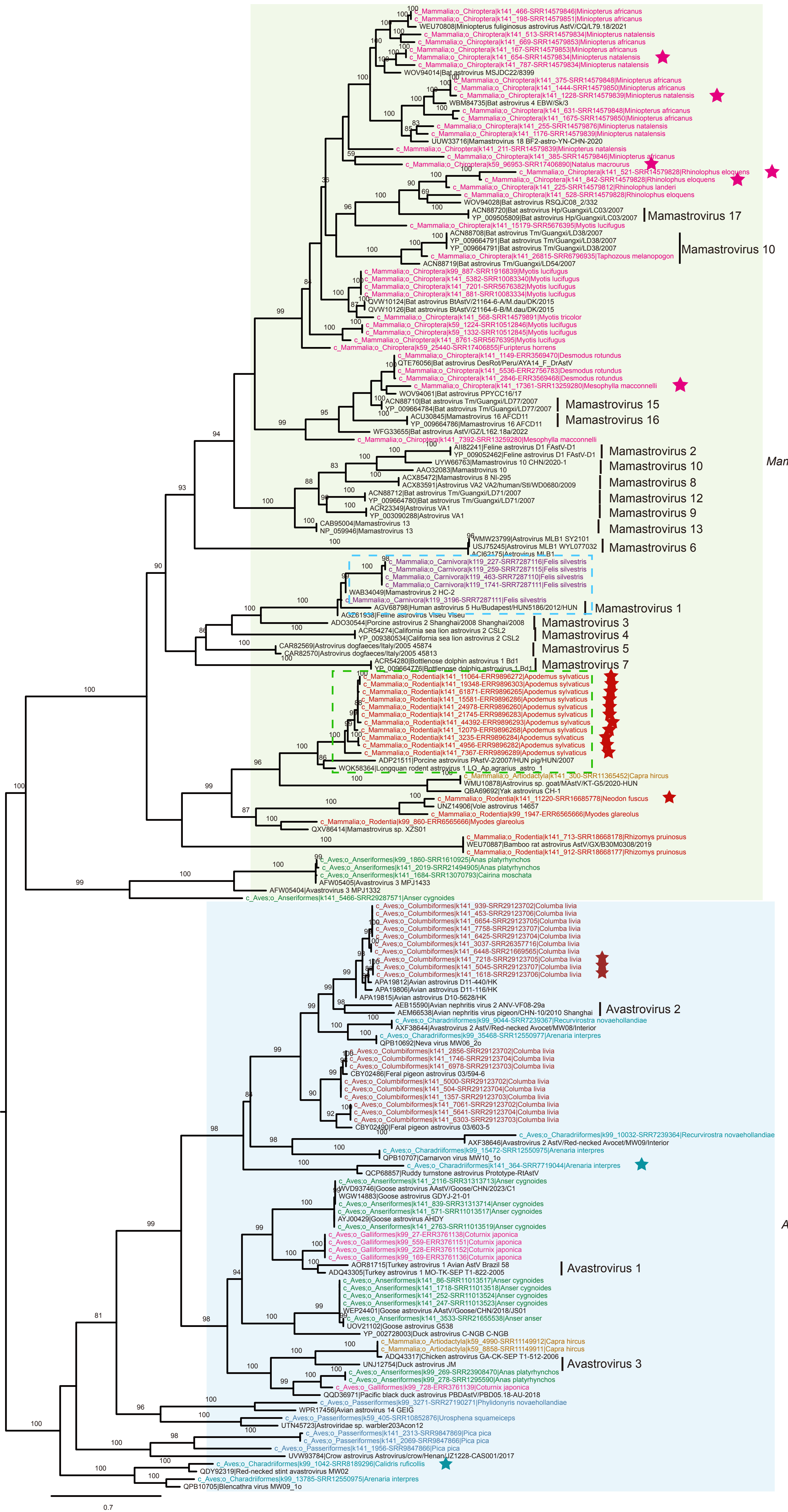

Fig S6

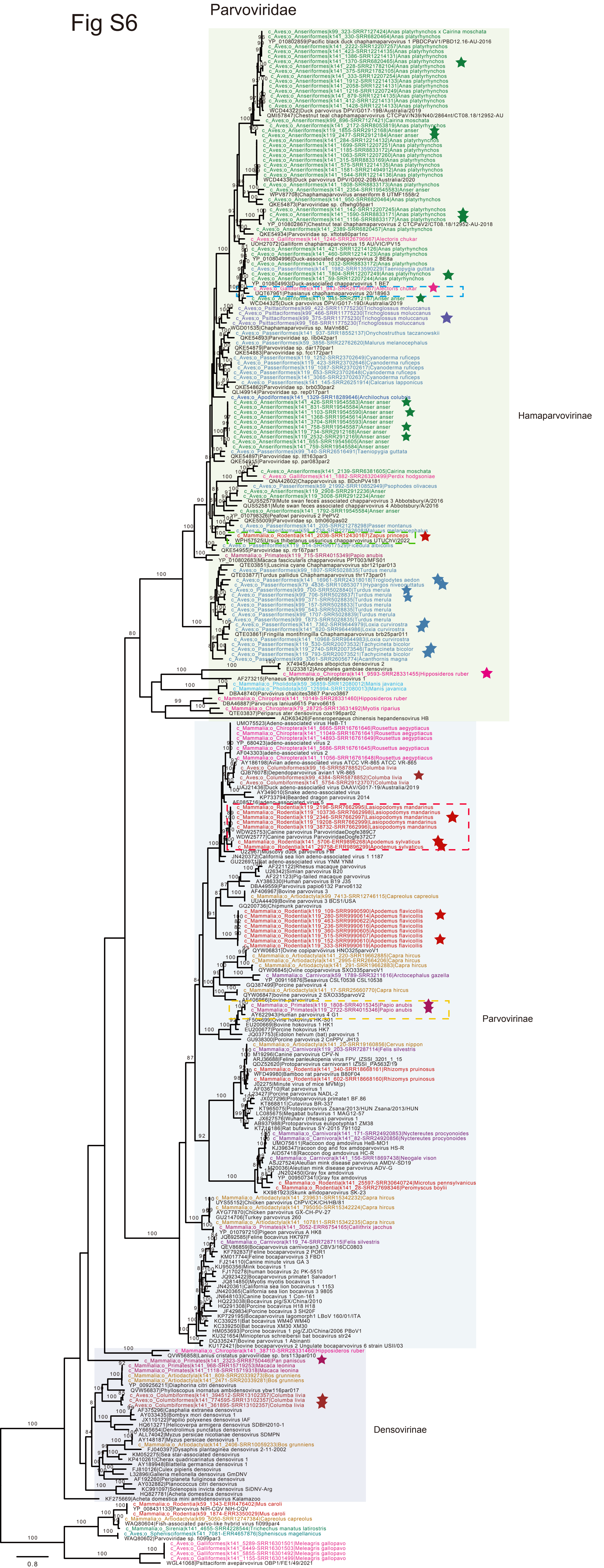
Parvoviridae
Hamaparvovirinae
Parvovirinae
Densovirinae
0.8

Fig S7

Anelloviridae

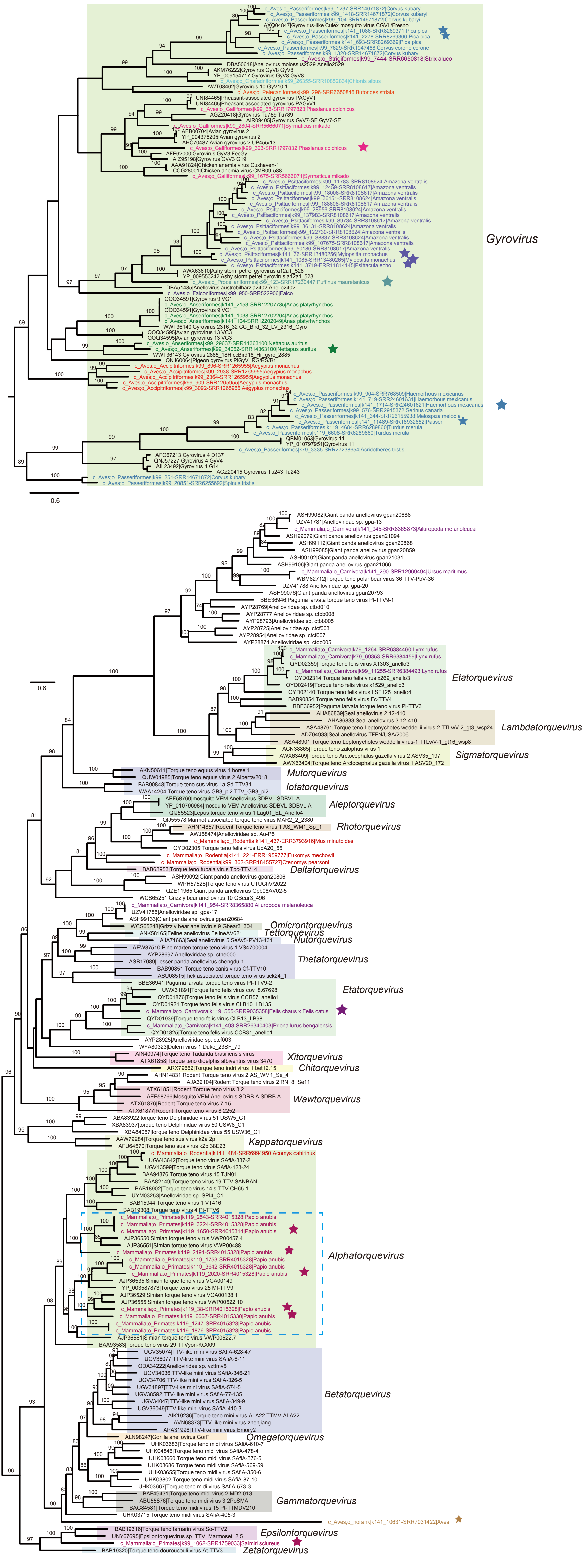

Fig S8 Circoviridae

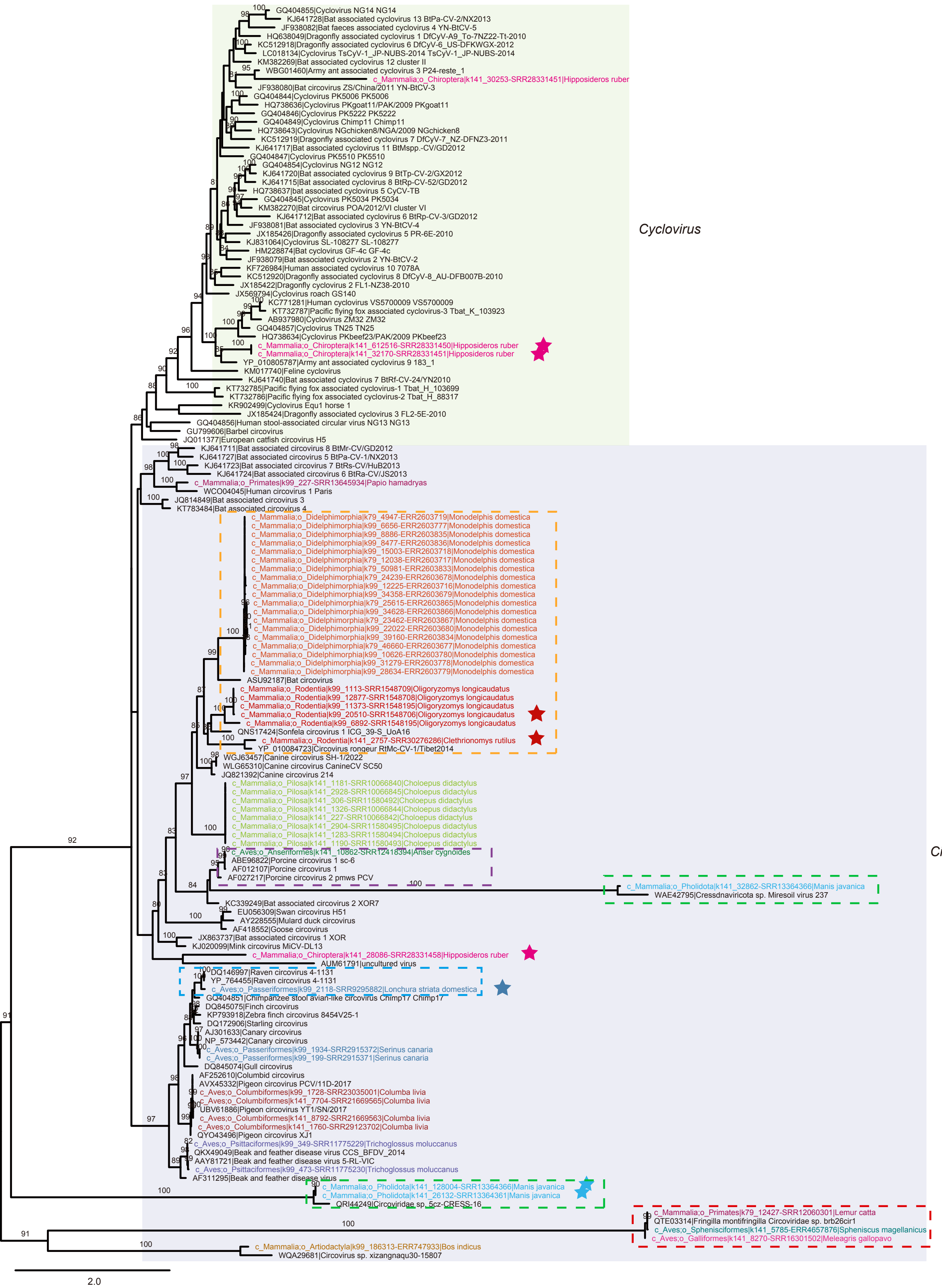

Fig S9

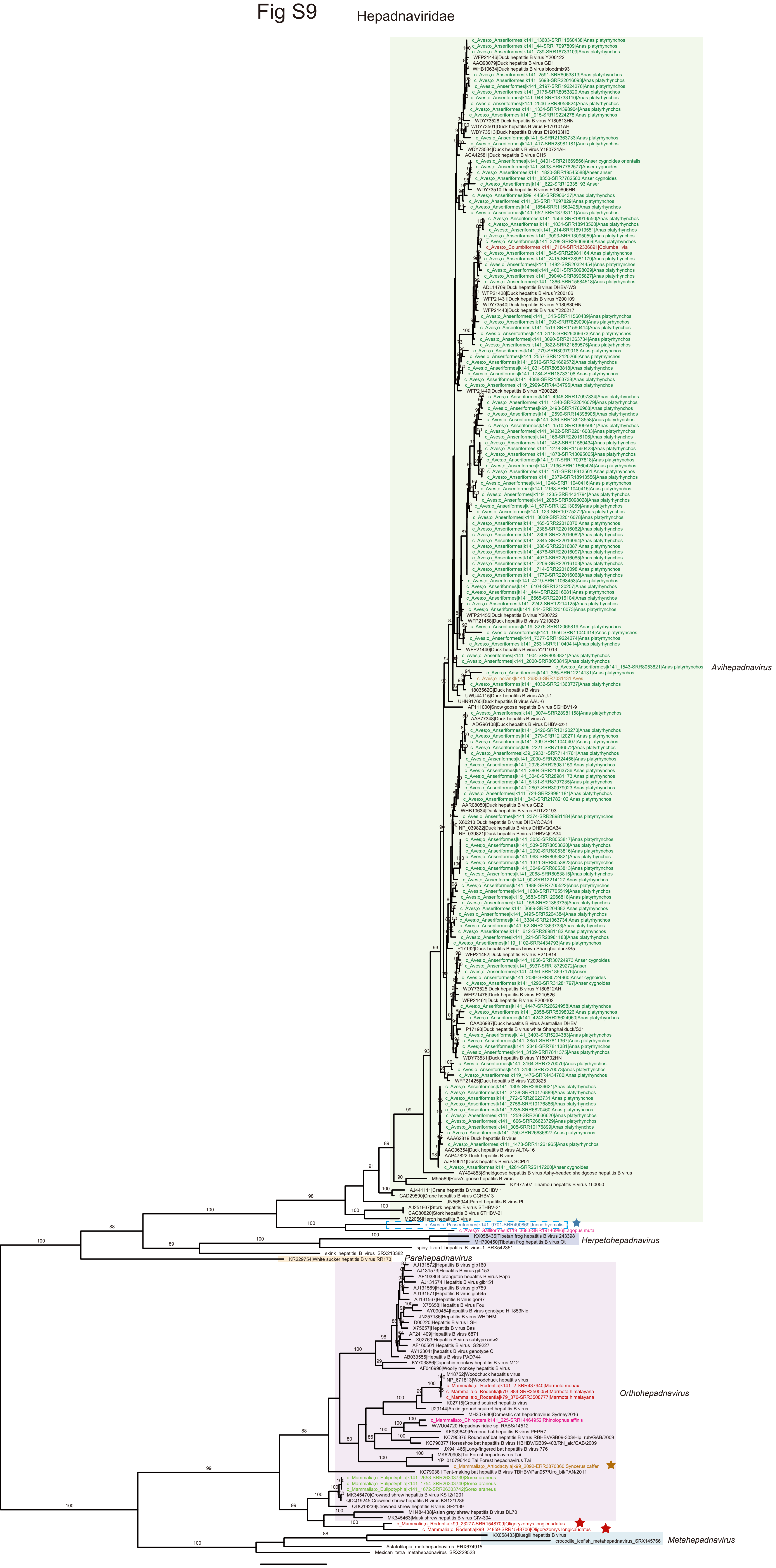

Hepadnaviridae
Avihepadnavirus
Herpetohepadnavirus
Parahepadnavirus
Orthohepadnavirus
Metahepadnavirus
0.6

Fig S10 Rhabdoviridae

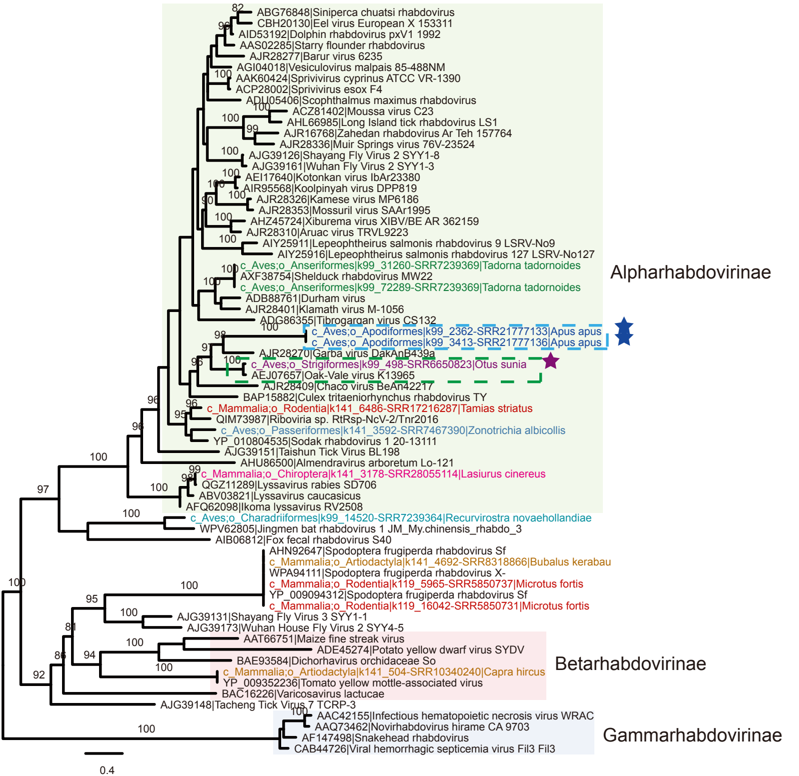

Fig S11

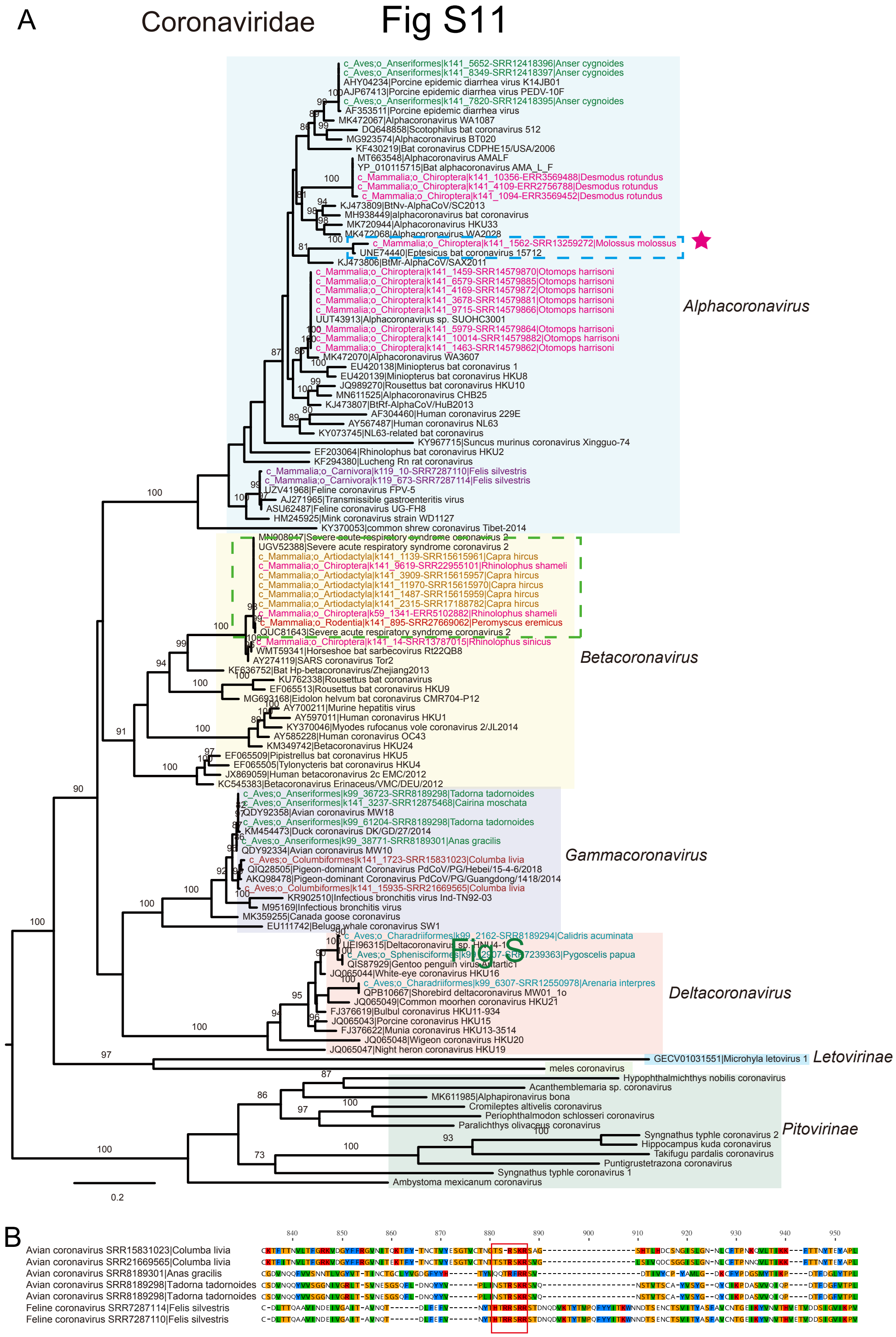

A
Coronaviridae
c_Aves;o_Anseriformes|k141_5652-SRR12418396|Anser cygnoides
c_Aves;o_Anseriformes|k141_8349-SRR12418397|Anser cygnoides
AHY04234|Porcine epidemic diarrhea virus K14JB01
AJP67413|Porcine epidemic diarrhea virus PEDV-10F
c_Aves;o_Anseriformes|k141_7820-SRR12418395|Anser cygnoides
AF353511|Porcine epidemic diarrhea virus
MK472067|Alphacoronavirus WA1087
DQ648858|Scotophilus bat coronavirus 512
MG923574|Alphacoronavirus BT020
KF430219|Bat coronavirus CDPHE15/USA/2006
MT663548|Alphacoronavirus AMALF
YP_010115715|Bat alphacoronavirus AMA_L_F
c_Mammalia;o_Chiroptera|k141_10356-ERR3569488|Desmodus rotundus
c_Mammalia;o_Chiroptera|k141_4109-ERR2756788|Desmodus rotundus
c_Mammalia;o_Chiroptera|k141_1094-ERR3569452|Desmodus rotundus
KJ473809|BtNv-AlphaCoV/SC2013
MH938449|alphacoronavirus bat coronavirus
MK720944|Alphacoronavirus HKU33
MK472068|Alphacoronavirus WA2028
c_Mammalia;o_Chiroptera|k141_1562-SRR13259272|Molossus molossus
UNE74440|Eptesicus bat coronavirus 15712
KJ473806|BtMr-AlphaCoV/SAX2011
c_Mammalia;o_Chiroptera|k141_1459-SRR14579870|Otomops harrisoni
c_Mammalia;o_Chiroptera|k141_6579-SRR14579885|Otomops harrisoni
c_Mammalia;o_Chiroptera|k141_4169-SRR14579872|Otomops harrisoni
c_Mammalia;o_Chiroptera|k141_3678-SRR14579881|Otomops harrisoni
c_Mammalia;o_Chiroptera|k141_9715-SRR14579866|Otomops harrisoni
UUT43913|Alphacoronavirus sp. SUOHC3001
c_Mammalia;o_Chiroptera|k141_5979-SRR14579864|Otomops harrisoni
c_Mammalia;o_Chiroptera|k141_10014-SRR14579882|Otomops harrisoni
c_Mammalia;o_Chiroptera|k141_1463-SRR14579862|Otomops harrisoni
MK472070|Alphacoronavirus WA3607
EU420138|Miniopterus bat coronavirus 1
EU420139|Miniopterus bat coronavirus HKU8
JQ989270|Rousettus bat coronavirus HKU10
MN611525|Alphacoronavirus CHB25
KJ473807|BtRf-AlphaCoV/HuB2013
AF304460|Human coronavirus 229E
AY567487|Human coronavirus NL63
KY073745|NL63-related bat coronavirus
KY967715|Suncus murinus coronavirus Xingguo-74
EF203064|Rhinolophus bat coronavirus HKU2
KF294380|Lucheng Rn rat coronavirus
c_Mammalia;o_Carnivora|k119_10-SRR7287110|Felis silvestris
c_Mammalia;o_Carnivora|k119_673-SRR7287114|Felis silvestris
UZV41968|Feline coronavirus FPV-5
AJ271965|Transmissible gastroenteritis virus
ASU62487|Feline coronavirus UG-FH8
HM245925|Mink coronavirus strain WD1127
KY370053|common shrew coronavirus Tibet-2014
Alphacoronavirus
MN908947|Severe acute respiratory syndrome coronavirus 2
UGV52388|Severe acute respiratory syndrome coronavirus 2
c_Mammalia;o_Artiodactyla|k141_1139-SRR15615961|Capra hircus
c_Mammalia;o_Chiroptera|k141_9619-SRR22955101|Rhinolophus shameli
c_Mammalia;o_Artiodactyla|k141_3909-SRR15615957|Capra hircus
c_Mammalia;o_Artiodactyla|k141_11970-SRR15615970|Capra hircus
c_Mammalia;o_Artiodactyla|k141_1487-SRR15615959|Capra hircus
c_Mammalia;o_Artiodactyla|k141_2315-SRR17188782|Capra hircus
c_Mammalia;o_Chiroptera|k59_1341-ERR5102882|Rhinolophus shameli
c_Mammalia;o_Rodentia|k141_895-SRR27669062|Peromyscus eremicus
QUC81643|Severe acute respiratory syndrome coronavirus 2
c_Mammalia;o_Chiroptera|k141_14-SRR13787015|Rhinolophus sinicus
WMT59341|Horseshoe bat sarbecovirus Rt22QB8
AY274119|SARS coronavirus Tor2
KF636752|Bat Hp-betacoronavirus/Zhejiang2013
KU762338|Rousettus bat coronavirus
EF065513|Rousettus bat coronavirus HKU9
MG693168|Eidolon helvum bat coronavirus CMR704-P12
AY700211|Murine hepatitis virus
AY597011|Human coronavirus HKU1
KY370046|Myodes rufocanus vole coronavirus 2/JL2014
AY585228|Human coronavirus OC43
KM349742|Betacoronavirus HKU24
EF065509|Pipistrellus bat coronavirus HKU5
EF065505|Tylonycteris bat coronavirus HKU4
JX869059|Human betacoronavirus 2c EMC/2012
KC545383|Betacoronavirus Erinaceus/VMC/DEU/2012
Betacoronavirus
c_Aves;o_Anseriformes|k99_36723-SRR8189298|Tadorna tadornoides
c_Aves;o_Anseriformes|k141_3237-SRR12875468|Cairina moschata
QDY92358|Avian coronavirus MW18
c_Aves;o_Anseriformes|k99_61204-SRR8189298|Tadorna tadornoides
KM454473|Duck coronavirus DK/GD/27/2014
c_Aves;o_Anseriformes|k99_38771-SRR8189301|Anas gracilis
QDY92334|Avian coronavirus MW10
c_Aves;o_Columbiformes|k141_1723-SRR15831023|Columba livia
QIQ28505|Pigeon-dominant Coronavirus PdCoV/PG/Hebei/15-4-6/2018
AKQ98478|Pigeon-dominant Coronavirus PdCoV/PG/Guangdong/1418/2014
c_Aves;o_Columbiformes|k141_15935-SRR21669565|Columba livia
KR902510|Infectious bronchitis virus Ind-TN92-03
M95169|Infectious bronchitis virus
MK359255|Canada goose coronavirus
EU111742|Beluga whale coronavirus SW1
Gammacoronavirus
c_Aves;o_Charadriiformes|k99_2162-SRR8189294|Calidris acuminata
UEI96315|Deltacoronavirus sp. HNU4-1
c_Aves;o_Sphenisciformes|k99_2907-SRR7239363|Pygoscelis papua
QIS87929|Gentoo penguin virus Antartic1
JQ065044|White-eye coronavirus HKU16
c_Aves;o_Charadriiformes|k99_6307-SRR12550978|Arenaria interpres
QPB10667|Shorebird deltacoronavirus MW01_1o
JQ065049|Common moorhen coronavirus HKU21
FJ376619|Bulbul coronavirus HKU11-934
JQ065043|Porcine coronavirus HKU15
FJ376622|Munia coronavirus HKU13-3514
JQ065048|Wigeon coronavirus HKU20
JQ065047|Night heron coronavirus HKU19
Deltacoronavirus
GECV01031551|Microhyla letovirus 1
Letovirinae
meles coronavirus
Hypophthalmichthys nobilis coronavirus
Acanthemblemaria sp. coronavirus
MK611985|Alphapironavirus bona
Cromileptes altivelis coronavirus
Periophthalmodon schlosseri coronavirus
Paralichthys olivaceus coronavirus
Syngnathus typhle coronavirus 2
Hippocampus kuda coronavirus
Takifugu pardalis coronavirus
Puntigrustetrazona coronavirus
Syngnathus typhle coronavirus 1
Ambystoma mexicanum coronavirus
Pitovirinae
0.2
B
840
850
860
870
880
890
900
910
920
930
940
950
Avian coronavirus SRR15831023|Columba livia
Avian coronavirus SRR21669565|Columba livia
Avian coronavirus SRR8189301|Anas gracilis
Avian coronavirus SRR8189298|Tadorna tadornoides
Avian coronavirus SRR8189298|Tadorna tadornoides
Feline coronavirus SRR7287114|Felis silvestris
Feline coronavirus SRR7287110|Felis silvestris

Fig S12

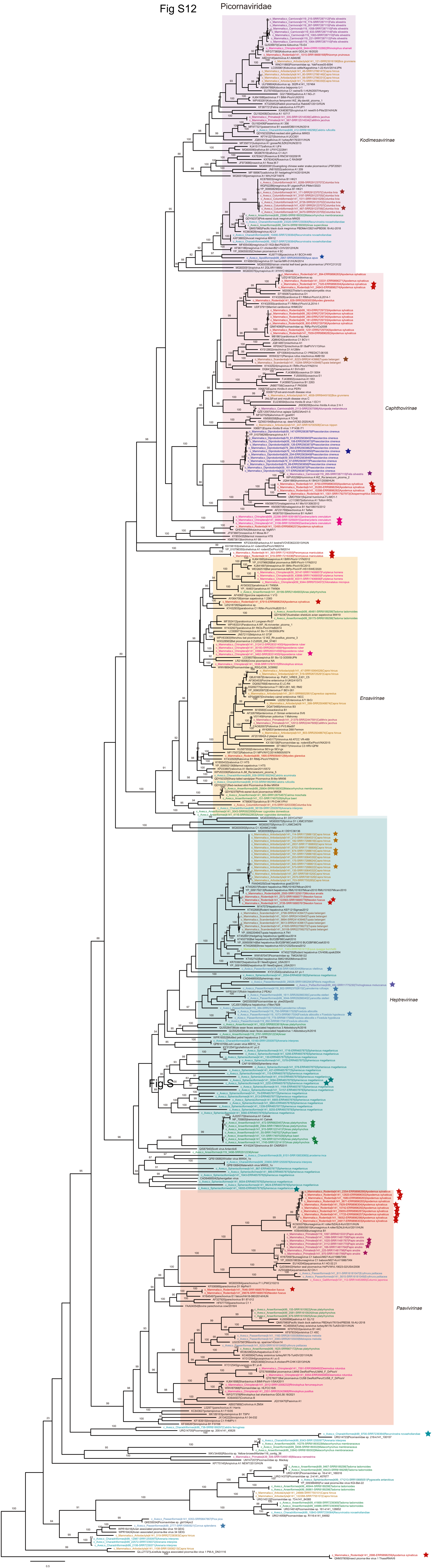

Picornaviridae
Kodimesavirinae
Caphthovirinae
Ensavirinae
Heptrevirinae
Paavivirinae
0.5

Polyomaviridae

Fig S13

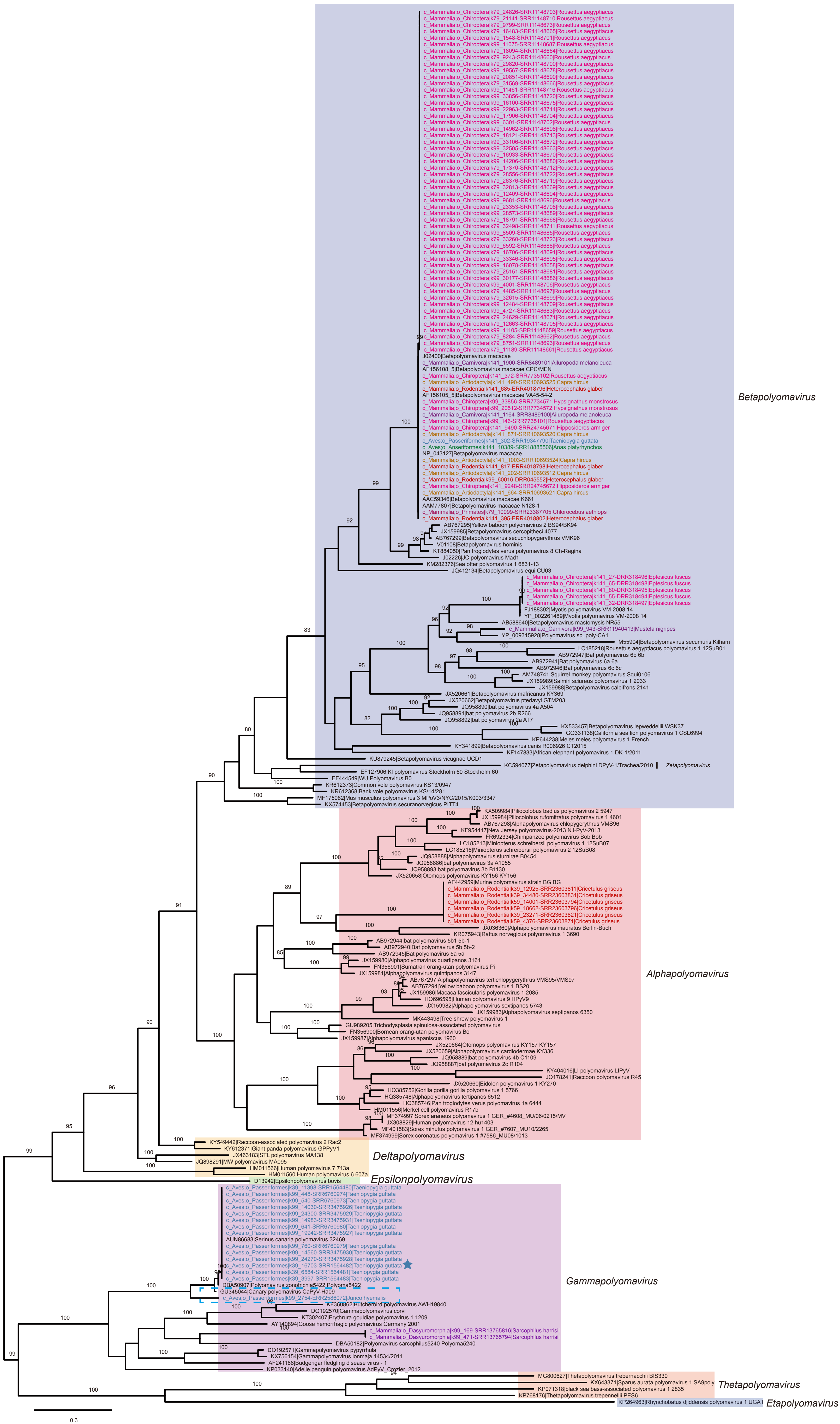

Fig S14 Peribunyaviridae

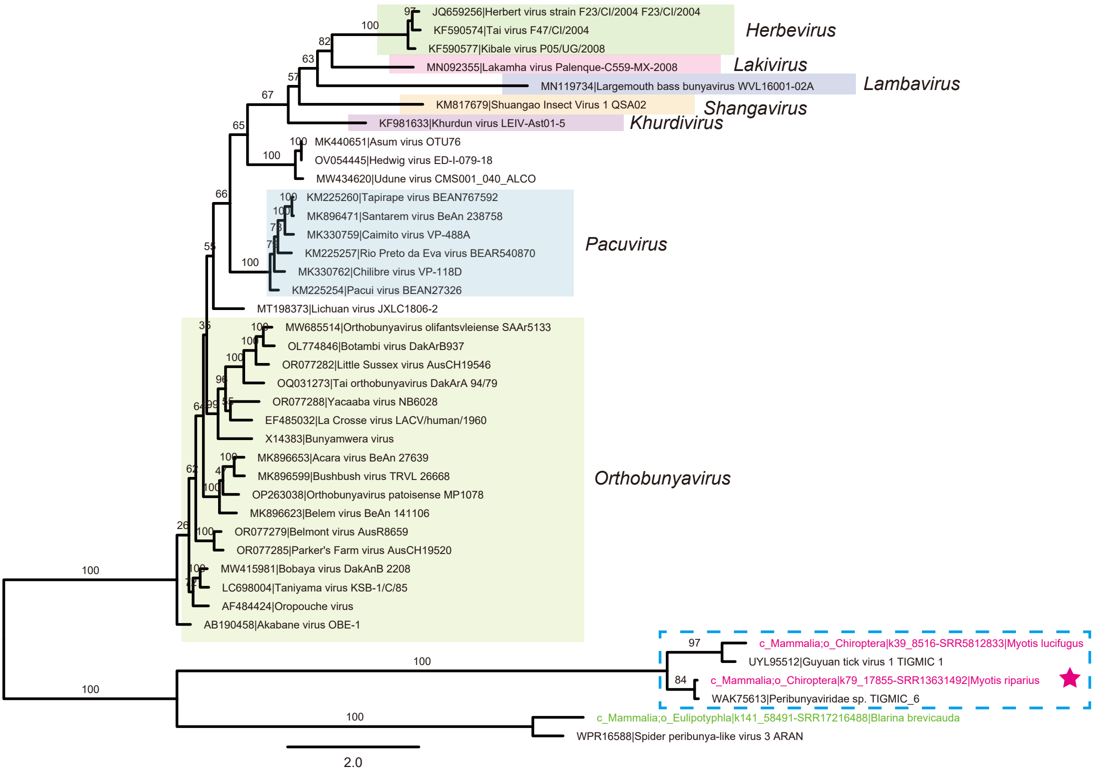

Fig S15

Caliciviridae

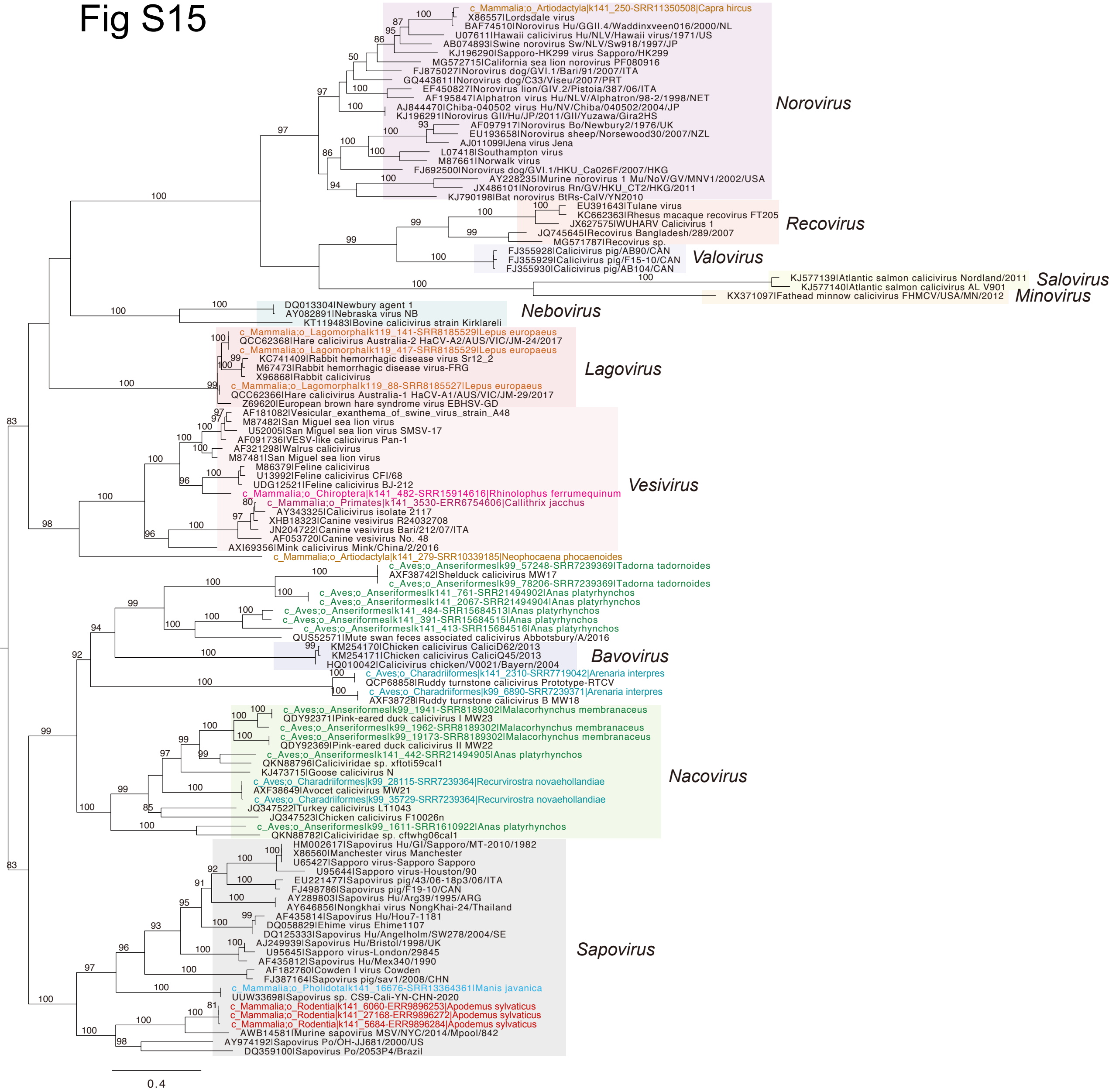

Fig S16

Hepeviridae

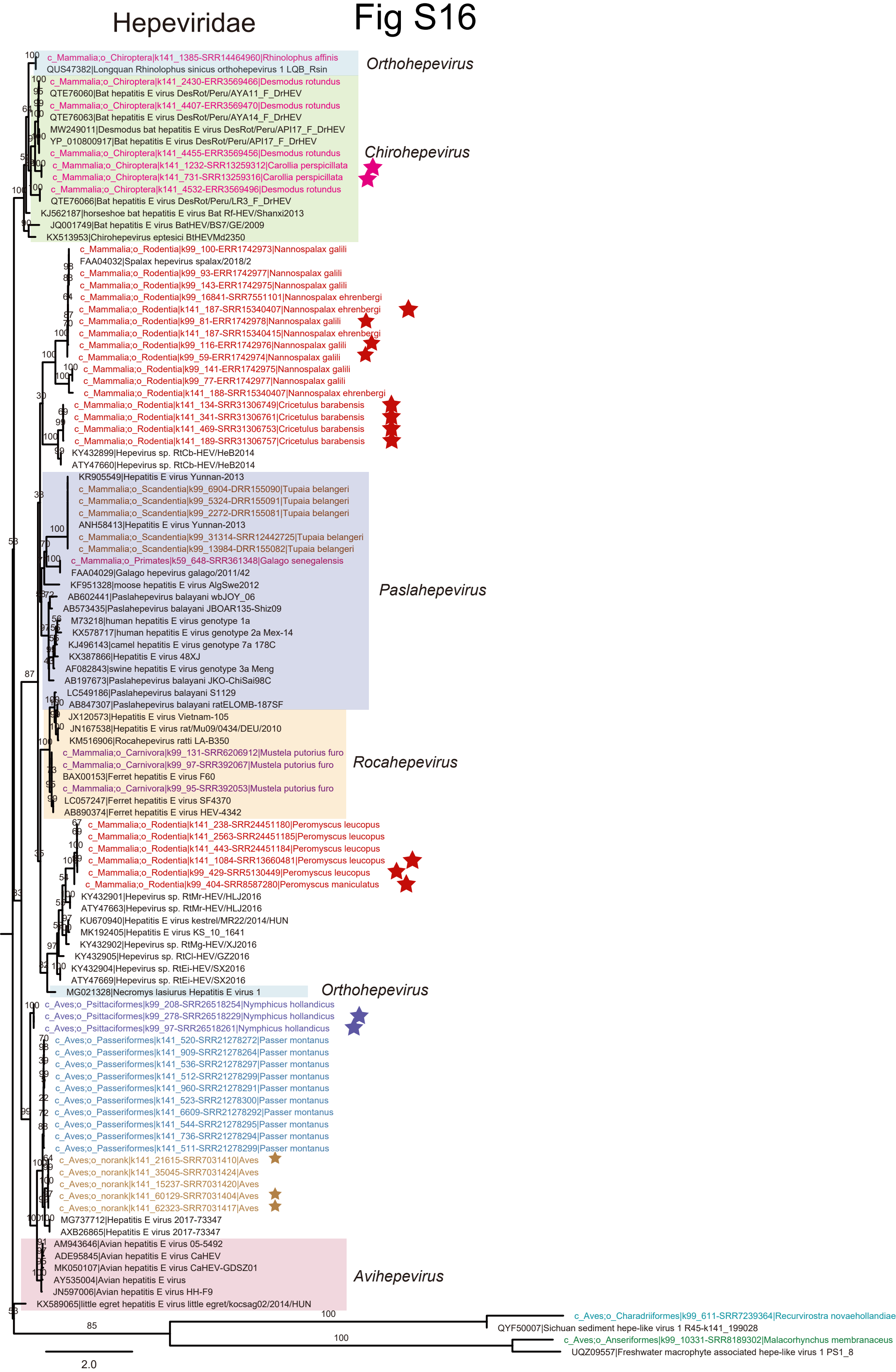

Fig S17 Orthoherpesviridae

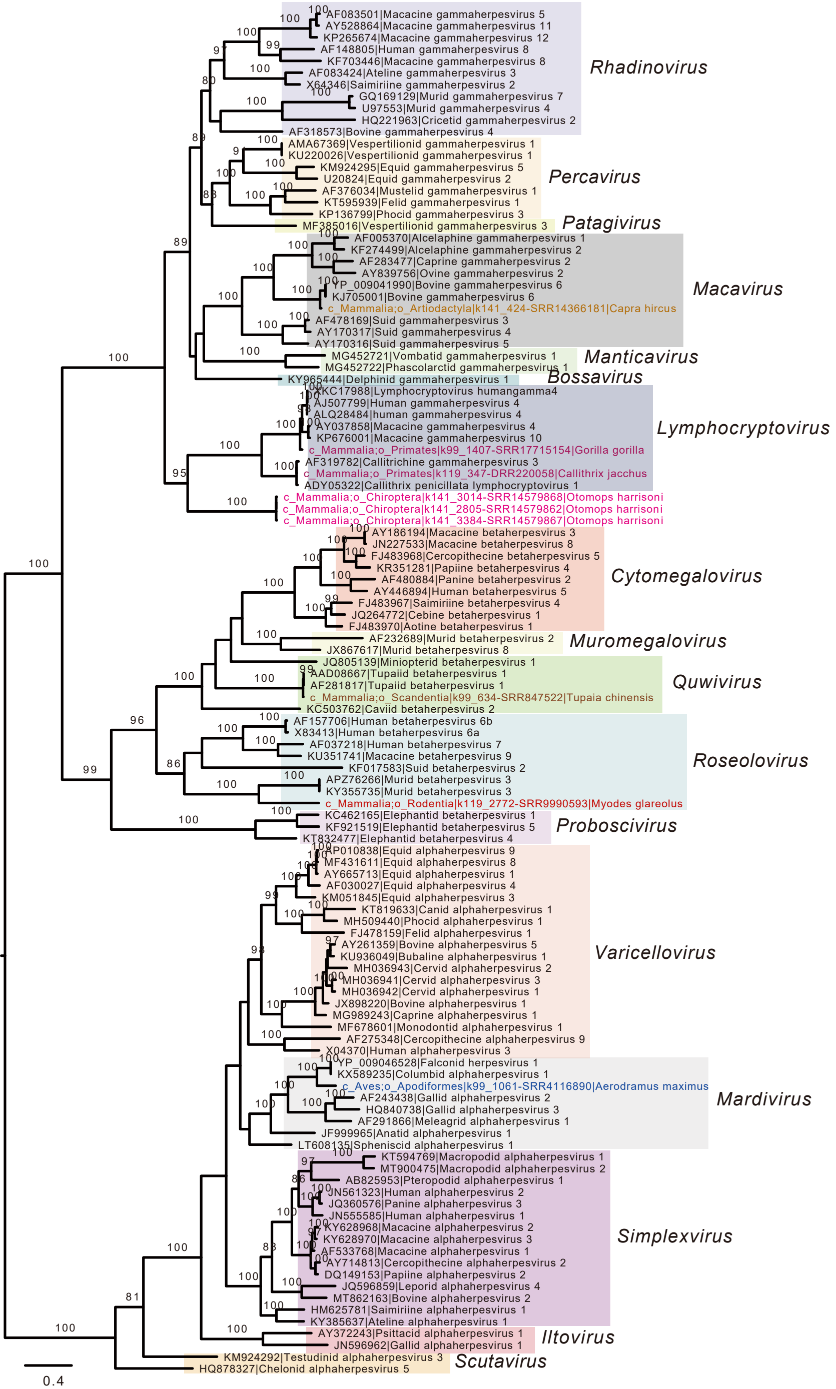

Fig S18

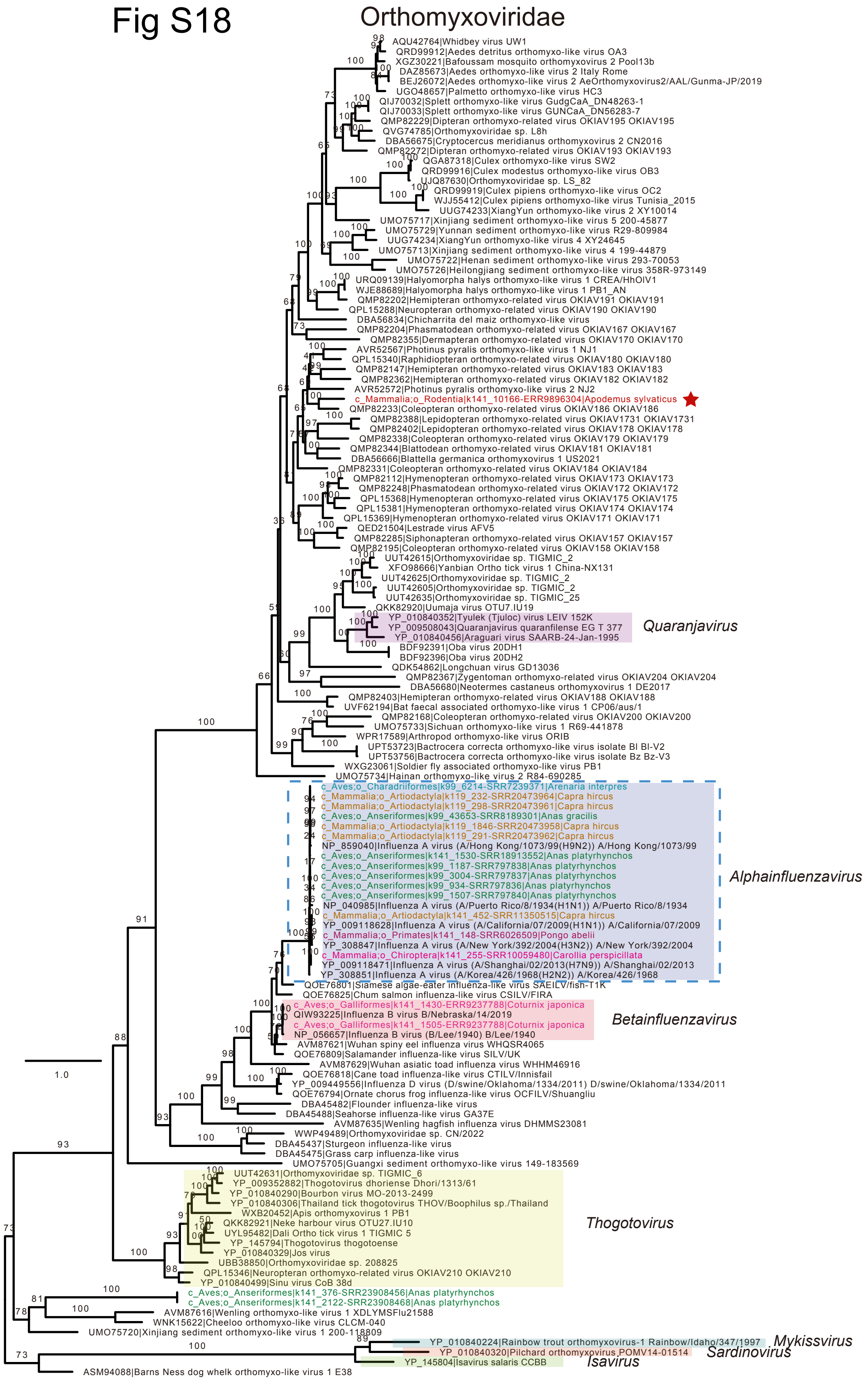
Orthomyxoviridae
AQU42764|Whidbey virus UW1
QRD99912|Aedes detritus orthomyxo-like virus OA3
XGZ30221|Bafoussam mosquito orthomyxovirus 2 Pool13b
DAZ85673|Aedes orthomyxo-like virus 2 Italy Rome
BEJ26072|Aedes orthomyxo-like virus 2 AeOrthomyxovirus2/AAL/Gunma-JP/2019
UGO48657|Palmetto orthomyxo-like virus HC3
QIJ70032|Splett orthomyxo-like virus GudgCaA_DN48263-1
QIJ70033|Splett orthomyxo-like virus GUNCaA_DN56283-7
QMP82229|Dipteran orthomyxo-related virus OKIAV195 OKIAV195
QVG74785|Orthomyxoviridae sp. L8h
DBA56675|Cryptocercus meridianus orthomyxovirus 2 CN2016
QMP82272|Dipteran orthomyxo-related virus OKIAV193 OKIAV193
QGA87318|Culex orthomyxo-like virus SW2
QRD99916|Culex modestus orthomyxo-like virus OB3
UJQ87630|Orthomyxoviridae sp. LS_82
QRD99919|Culex pipiens orthomyxo-like virus OC2
WJJ55412|Culex pipiens orthomyxo-like virus Tunisia_2015
UUG74233|XiangYun orthomyxo-like virus 2 XY10014
UMO75717|Xinjiang sediment orthomyxo-like virus 5 200-45877
UMO75729|Yunnan sediment orthomyxo-like virus R29-809984
UUG74234|XiangYun orthomyxo-like virus 4 XY24645
UMO75713|Xinjiang sediment orthomyxo-like virus 4 199-44879
UMO75722|Henan sediment orthomyxo-like virus 293-70053
UMO75726|Heilongjiang sediment orthomyxo-like virus 358R-973149
URQ09139|Halyomorpha halys orthomyxo-like virus 1 CREA/HhOIV1
WJE88689|Halyomorpha halys orthomyxo-like virus 1 PB1_AN
QMP82202|Hemipteran orthomyxo-related virus OKIAV191 OKIAV191
QPL15288|Neuropteran orthomyxo-related virus OKIAV190 OKIAV190
DBA56834|Chicharrita del maiz orthomyxo-like virus
QMP82204|Phasmatodean orthomyxo-related virus OKIAV167 OKIAV167
QMP82355|Dermapteran orthomyxo-related virus OKIAV170 OKIAV170
AVR52567|Photinus pyralis orthomyxo-like virus 1 NJ1
QPL15340|Raphidiopteran orthomyxo-related virus OKIAV180 OKIAV180
QMP82147|Hemipteran orthomyxo-related virus OKIAV183 OKIAV183
QMP82362|Hemipteran orthomyxo-related virus OKIAV182 OKIAV182
AVR52572|Photinus pyralis orthomyxo-like virus 2 NJ2
c_Mammalia;o_Rodentia|k141_10166-ERR9896304|Apodemus sylvaticus
QMP82233|Coleopteran orthomyxo-related virus OKIAV186 OKIAV186
QMP82388|Lepidopteran orthomyxo-related virus OKIAV1731 OKIAV1731
QMP82402|Lepidopteran orthomyxo-related virus OKIAV178 OKIAV178
QMP82338|Coleopteran orthomyxo-related virus OKIAV179 OKIAV179
QMP82344|Blattodean orthomyxo-related virus OKIAV181 OKIAV181
DBA56666|Blattella germanica orthomyxovirus 1 US2021
QMP82331|Coleopteran orthomyxo-related virus OKIAV184 OKIAV184
QMP82112|Hymenopteran orthomyxo-related virus OKIAV173 OKIAV173
QMP82248|Phasmatodean orthomyxo-related virus OKIAV172 OKIAV172
QPL15368|Hymenopteran orthomyxo-related virus OKIAV175 OKIAV175
QPL15381|Hymenopteran orthomyxo-related virus OKIAV174 OKIAV174
QPL15369|Hymenopteran orthomyxo-related virus OKIAV171 OKIAV171
QED21504|Lestrade virus AFV5
QMP82285|Siphonapteran orthomyxo-related virus OKIAV157 OKIAV157
QMP82195|Coleopteran orthomyxo-related virus OKIAV158 OKIAV158
UUT42615|Orthomyxoviridae sp. TIGMIC_2
XFO98666|Yanbian Ortho tick virus 1 China-NX131
UUT42625|Orthomyxoviridae sp. TIGMIC_2
UUT42605|Orthomyxoviridae sp. TIGMIC_2
UUT42635|Orthomyxoviridae sp. TIGMIC_25
QKK82920|Uumaja virus OTU7.IU19
YP_010840352|Tyulek (Tjuloc) virus LEIV 152K
YP_009508043|Quaranjavirus quaranfilense EG T 377
YP_010840456|Araguari virus SAARB-24-Jan-1995
Quaranjavirus
BDF92391|Oba virus 20DH1
BDF92396|Oba virus 20DH2
QDK54862|Longchuan virus GD13036
QMP82367|Zygentoman orthomyxo-related virus OKIAV204 OKIAV204
DBA56680|Neotermes castaneus orthomyxovirus 1 DE2017
QMP82403|Hemipteran orthomyxo-related virus OKIAV188 OKIAV188
UVF62194|Bat faecal associated orthomyxo-like virus 1 CP06/aus/1
QMP82168|Coleopteran orthomyxo-related virus OKIAV200 OKIAV200
UMO75733|Sichuan orthomyxo-like virus 1 R69-441878
WPR17589|Arthropod orthomyxo-like virus ORIB
UPT53723|Bactrocera correcta orthomyxo-like virus isolate BI BI-V2
UPT53756|Bactrocera correcta orthomyxo-like virus isolate Bz Bz-V3
WXG23061|Soldier fly associated orthomyxo-like virus PB1
UMO75734|Hainan orthomyxo-like virus 2 R84-690285
c_Aves;o_Charadriiformes|k99_6214-SRR7239371|Arenaria interpres
c_Mammalia;o_Artiodactyla|k119_232-SRR20473964|Capra hircus
c_Mammalia;o_Artiodactyla|k119_298-SRR20473961|Capra hircus
c_Aves;o_Anseriformes|k99_43653-SRR8189301|Anas gracilis
c_Mammalia;o_Artiodactyla|k119_1846-SRR20473958|Capra hircus
c_Mammalia;o_Artiodactyla|k119_291-SRR20473962|Capra hircus
NP_859040|Influenza A virus (A/Hong Kong/1073/99(H9N2)) A/Hong Kong/1073/99
c_Aves;o_Anseriformes|k141_1530-SRR18913552|Anas platyrhynchos
c_Aves;o_Anseriformes|k99_1187-SRR797838|Anas platyrhynchos
c_Aves;o_Anseriformes|k99_3004-SRR797837|Anas platyrhynchos
c_Aves;o_Anseriformes|k99_934-SRR797836|Anas platyrhynchos
c_Aves;o_Anseriformes|k99_1507-SRR797840|Anas platyrhynchos
NP_040985|Influenza A virus (A/Puerto Rico/8/1934(H1N1)) A/Puerto Rico/8/1934
c_Mammalia;o_Artiodactyla|k141_452-SRR11350515|Capra hircus
YP_009118628|Influenza A virus (A/California/07/2009(H1N1)) A/California/07/2009
c_Mammalia;o_Primates|k141_148-SRR6026509|Pongo abelii
YP_308847|Influenza A virus (A/New York/392/2004(H3N2)) A/New York/392/2004
c_Mammalia;o_Chiroptera|k141_255-SRR10059480|Carollia perspicillata
YP_009118471|Influenza A virus (A/Shanghai/02/2013(H7N9)) A/Shanghai/02/2013
YP_308851|Influenza A virus (A/Korea/426/1968(H2N2)) A/Korea/426/1968
Alphainfluenzavirus
QOE76801|Siamese algae-eater influenza-like virus SAEILV/fish-T1K
QOE76825|Chum salmon influenza-like virus CSILV/FIRA
c_Aves;o_Galliformes|k141_1430-ERR9237788|Coturnix japonica
QIW93225|Influenza B virus B/Nebraska/14/2019
c_Aves;o_Galliformes|k141_1505-ERR9237788|Coturnix japonica
NP_056657|Influenza B virus (B/Lee/1940) B/Lee/1940
Betainfluenzavirus
AVM87621|Wuhan spiny eel influenza virus WHQSR4065
QOE76809|Salamander influenza-like virus SILV/UK
AVM87629|Wuhan asiatic toad influenza virus WHHM46916
QOE76818|Cane toad influenza-like virus CTILV/Innisfail
YP_009449556|Influenza D virus (D/swine/Oklahoma/1334/2011) D/swine/Oklahoma/1334/2011
QOE76794|Ornate chorus frog influenza-like virus OCFILV/Shuangliu
DBA45482|Flounder influenza-like virus
DBA45488|Seahorse influenza-like virus GA37E
AVM87635|Wenling hagfish influenza virus DHMMS23081
WWP49489|Orthomyxoviridae sp. CN/2022
DBA45437|Sturgeon influenza-like virus
DBA45475|Grass carp influenza-like virus
UMO75705|Guangxi sediment orthomyxo-like virus 149-183569
UUT42631|Orthomyxoviridae sp. TIGMIC_6
YP_009352882|Thogotovirus dhoriense Dhori/1313/61
YP_010840290|Bourbon virus MO-2013-2499
YP_010840306|Thailand tick thogotovirus THOV/Boophilus sp./Thailand
WXB20452|Apis orthomyxovirus 1 PB1
QKK82921|Neke harbour virus OTU27.IU10
UYL95482|Dali Ortho tick virus 1 TIGMIC 5
YP_145794|Thogotovirus thogotoense
YP_010840329|Jos virus
UBB38850|Orthomyxoviridae sp. 208825
QPL15346|Neuropteran orthomyxo-related virus OKIAV210 OKIAV210
YP_010840499|Sinu virus CoB 38d
Thogotovirus
c_Aves;o_Anseriformes|k141_376-SRR23908456|Anas platyrhynchos
c_Aves;o_Anseriformes|k141_2122-SRR23908468|Anas platyrhynchos
AVM87616|Wenling orthomyxo-like virus 1 XDLYMSFlu21588
WNK15622|Cheeloo orthomyxo-like virus CLCM-040
UMO75720|Xinjiang sediment orthomyxo-like virus 1 200-118809
YP_010840224|Rainbow trout orthomyxovirus-1 Rainbow/Idaho/347/1997
Mykissvirus
YP_010840320|Pilchard orthomyxovirus POMV14-01514
Sardinovirus
YP_145804|Isavirus salaris CCBB
Isavirus
ASM94088|Barns Ness dog whelk orthomyxo-like virus 1 E38
1.0

Fig 19 Papillomaviridae

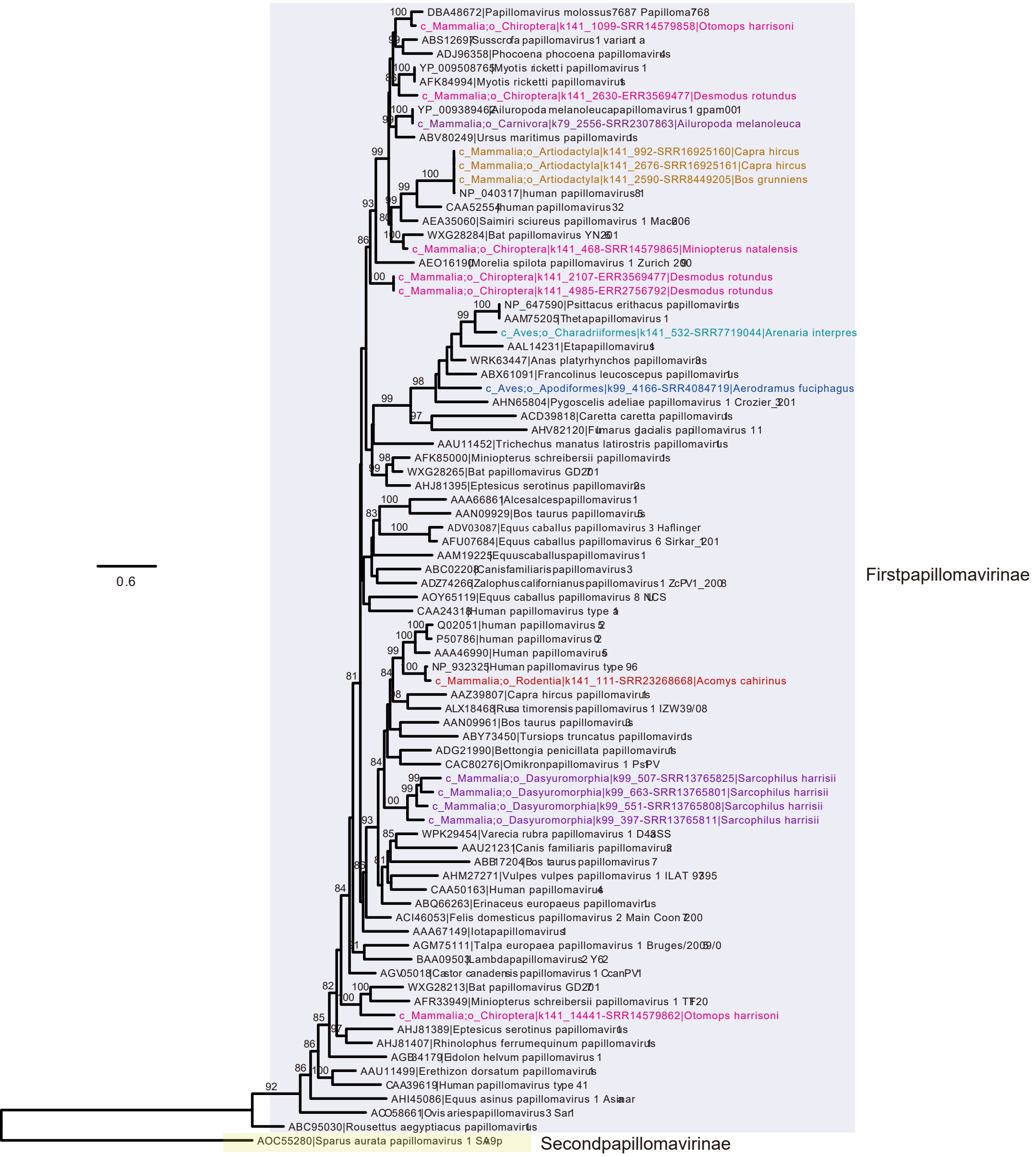

Fig S20

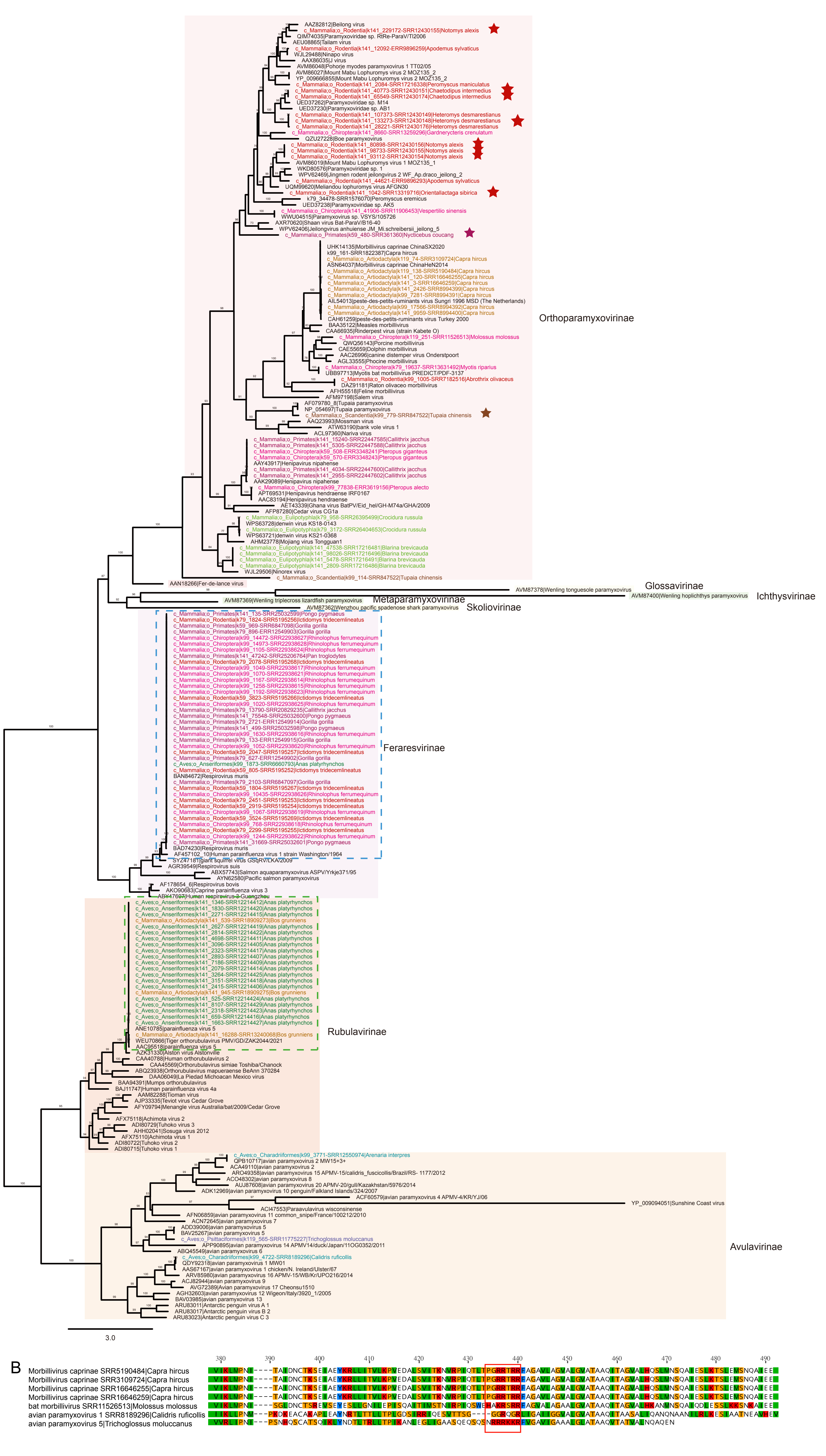
A
Paramyxoviridae
Orthoparamyxovirinae
Glossavirinae
Ichthysvirinae
Metaparamyxovirinae
Skoliovirinae
Feraresvirinae
Rubulavirinae
Avulavirinae
3.0
B
Morbillivirus caprinae SRR5190484|Capra hircus
Morbillivirus caprinae SRR3109724|Capra hircus
Morbillivirus caprinae SRR16646255|Capra hircus
Morbillivirus caprinae SRR16646259|Capra hircus
bat morbillivirus SRR11526513|Molossus molossus
avian paramyxovirus 1 SRR8189296|Calidris ruficollis
avian paramyxovirus 5|Trichoglossus moluccanus

Picobirnaviridae Fig S21

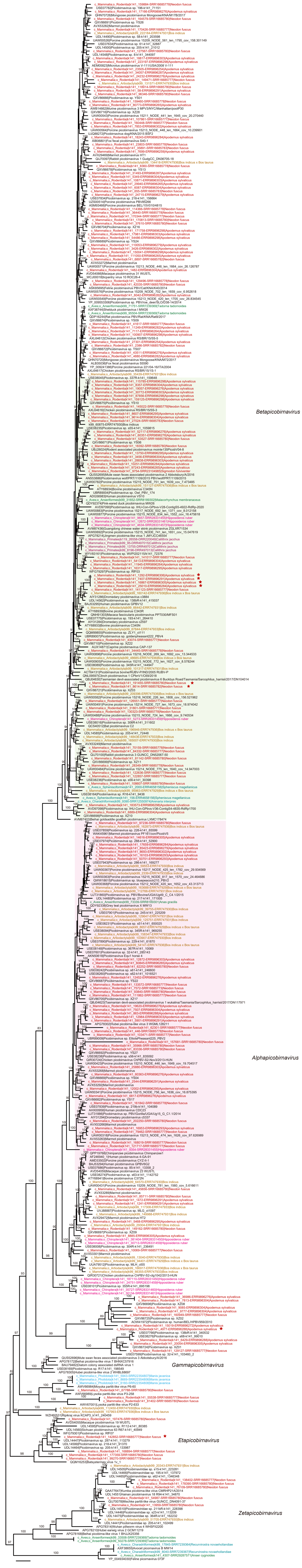

Fig S22

Adenoviridae

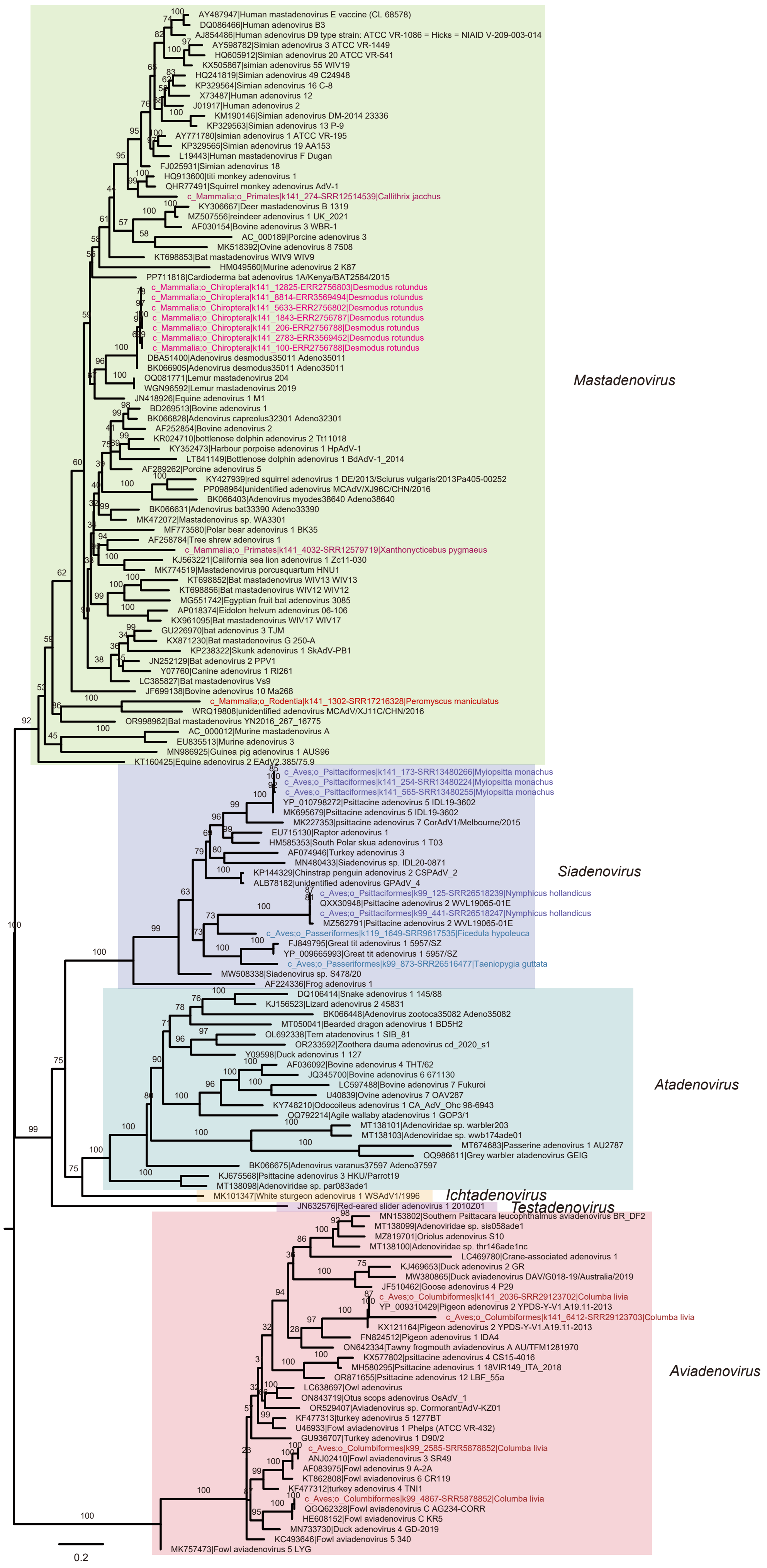

Fig S23

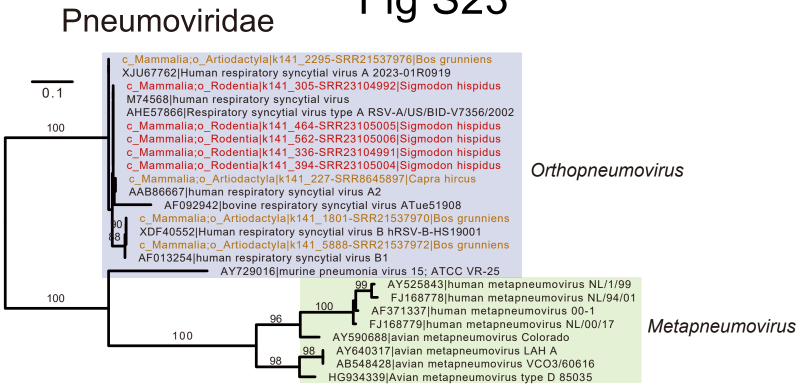

Pneumoviridae
0.1
100
100
100
96
98
100
99
98
90
88
c_Mammalia;o_Artiodactyla|k141_2295-SRR21537976|Bos grunniens
XJU67762|Human respiratory syncytial virus A 2023-01R0919
c_Mammalia;o_Rodentia|k141_305-SRR23104992|Sigmodon hispidus
M74568|human respiratory syncytial virus
AHE57866|Respiratory syncytial virus type A RSV-A/US/BID-V7356/2002
c_Mammalia;o_Rodentia|k141_464-SRR23105005|Sigmodon hispidus
c_Mammalia;o_Rodentia|k141_562-SRR23105006|Sigmodon hispidus
c_Mammalia;o_Rodentia|k141_336-SRR23104991|Sigmodon hispidus
c_Mammalia;o_Rodentia|k141_394-SRR23105004|Sigmodon hispidus
c_Mammalia;o_Artiodactyla|k141_227-SRR8645897|Capra hircus
AAB86667|human respiratory syncytial virus A2
AF092942|bovine respiratory syncytial virus ATue51908
c_Mammalia;o_Artiodactyla|k141_1801-SRR21537970|Bos grunniens
XDF40552|Human respiratory syncytial virus B hRSV-B-HS19001
c_Mammalia;o_Artiodactyla|k141_5888-SRR21537972|Bos grunniens
AF013254|human respiratory syncytial virus B1
AY729016|murine pneumonia virus 15; ATCC VR-25
Orthopneumovirus
AY525843|human metapneumovirus NL/1/99
FJ168778|human metapneumovirus NL/94/01
AF371337|human metapneumovirus 00-1
FJ168779|human metapneumovirus NL/00/17
AY590688|avian metapneumovirus Colorado
AY640317|avian metapneumovirus LAH A
AB548428|avian metapneumovirus VCO3/60616
HG934339|Avian metapneumovirus type D 85035
Metapneumovirus

Fig S24 Poxviridae

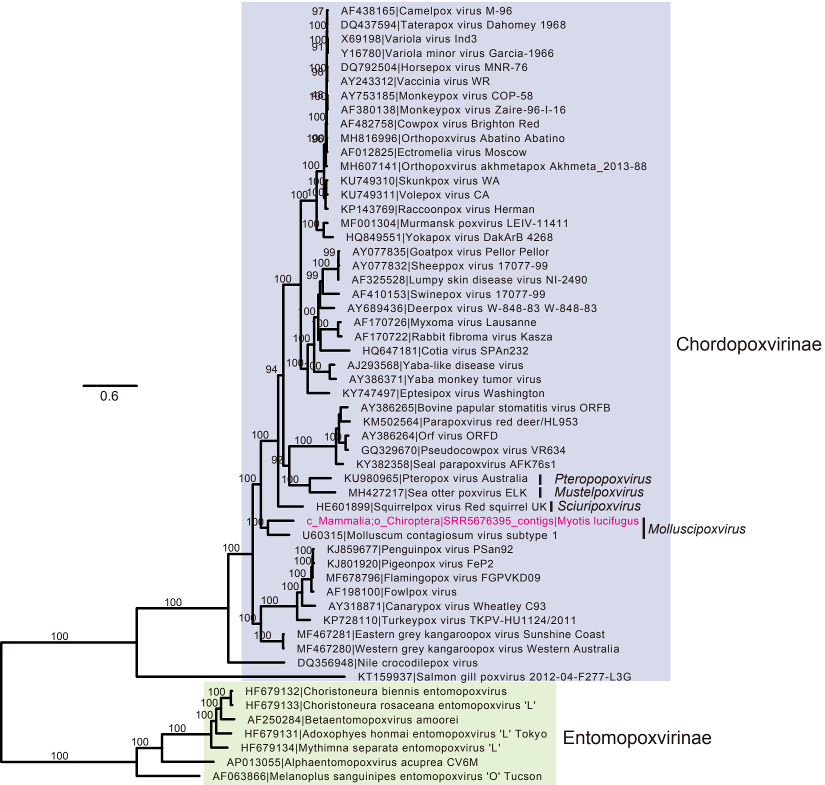

Fig S25

Reovirales

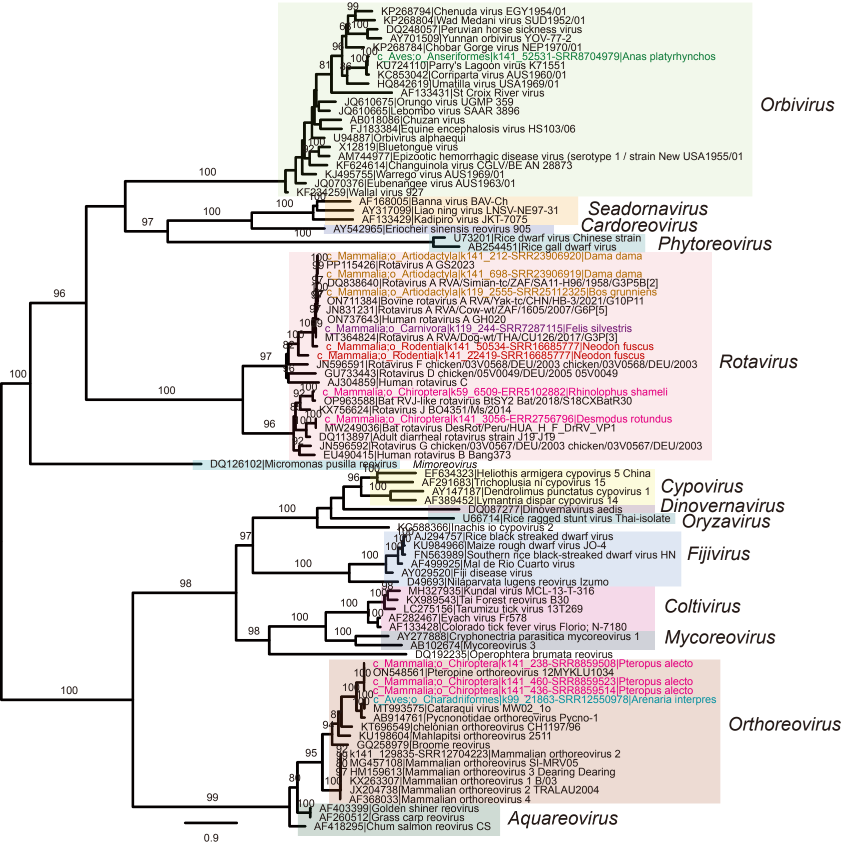

Fig S26

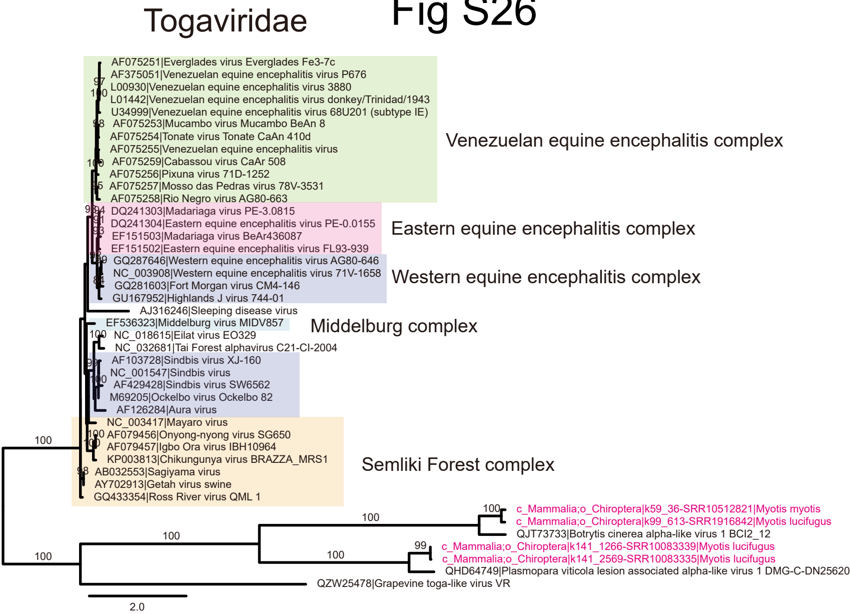
Togaviridae
AF075251|Everglades virus Everglades Fe3-7c
AF375051|Venezuelan equine encephalitis virus P676
L00930|Venezuelan equine encephalitis virus 3880
L01442|Venezuelan equine encephalitis virus donkey/Trinidad/1943
U34999|Venezuelan equine encephalitis virus 68U201 (subtype IE)
AF075253|Mucambo virus Mucambo BeAn 8
AF075254|Tonate virus Tonate CaAn 410d
AF075255|Venezuelan equine encephalitis virus
AF075259|Cabassou virus CaAr 508
AF075256|Pixuna virus 71D-1252
AF075257|Mosso das Pedras virus 78V-3531
AF075258|Rio Negro virus AG80-663
Venezuelan equine encephalitis complex
DQ241303|Madariaga virus PE-3.0815
DQ241304|Eastern equine encephalitis virus PE-0.0155
EF151503|Madariaga virus BeAr436087
EF151502|Eastern equine encephalitis virus FL93-939
Eastern equine encephalitis complex
GQ287646|Western equine encephalitis virus AG80-646
NC_003908|Western equine encephalitis virus 71V-1658
GQ281603|Fort Morgan virus CM4-146
GU167952|Highlands J virus 744-01
Western equine encephalitis complex
AJ316246|Sleeping disease virus
EF536323|Middelburg virus MIDV857
Middelburg complex
NC_018615|Eilat virus EO329
NC_032681|Tai Forest alphavirus C21-CI-2004
AF103728|Sindbis virus XJ-160
NC_001547|Sindbis virus
AF429428|Sindbis virus SW6562
M69205|Ockelbo virus Ockelbo 82
AF126284|Aura virus
NC_003417|Mayaro virus
AF079456|Onyong-nyong virus SG650
AF079457|Igbo Ora virus IBH10964
KP003813|Chikungunya virus BRAZZA_MRS1
AB032553|Sagiyama virus
AY702913|Getah virus swine
GQ433354|Ross River virus QML 1
Semliki Forest complex
c_Mammalia;o_Chiroptera|k59_36-SRR10512821|Myotis myotis
c_Mammalia;o_Chiroptera|k99_613-SRR1916842|Myotis lucifugus
QJT73733|Botrytis cinerea alpha-like virus 1 BCI2_12
c_Mammalia;o_Chiroptera|k141_1266-SRR10083339|Myotis lucifugus
c_Mammalia;o_Chiroptera|k141_2569-SRR10083335|Myotis lucifugus
QHD64749|Plasmopara viticola lesion associated alpha-like virus 1 DMG-C-DN25620
QZW25478|Grapevine toga-like virus VR
2.0

Fig S27

Flaviviridae

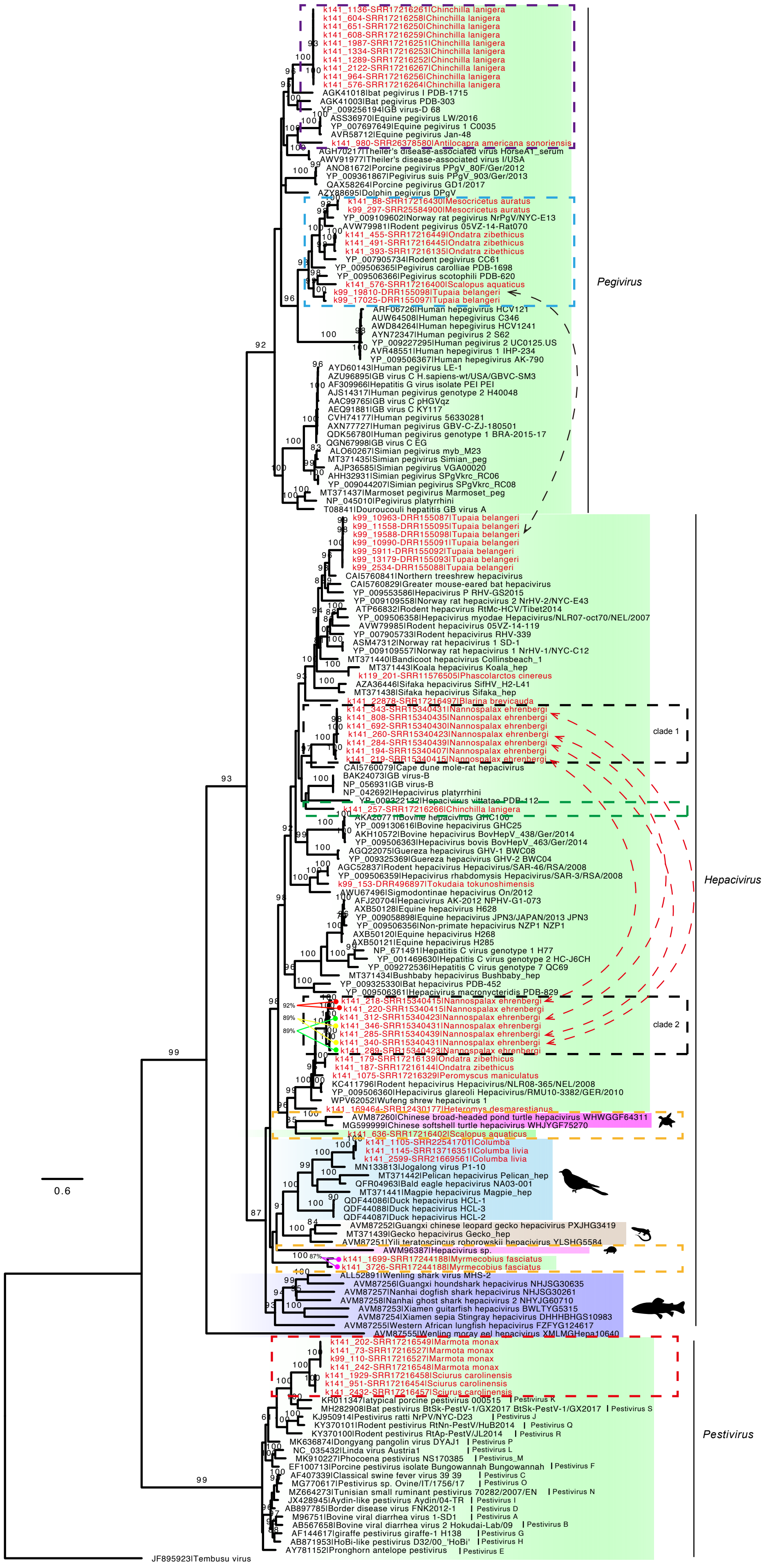

Fig S28

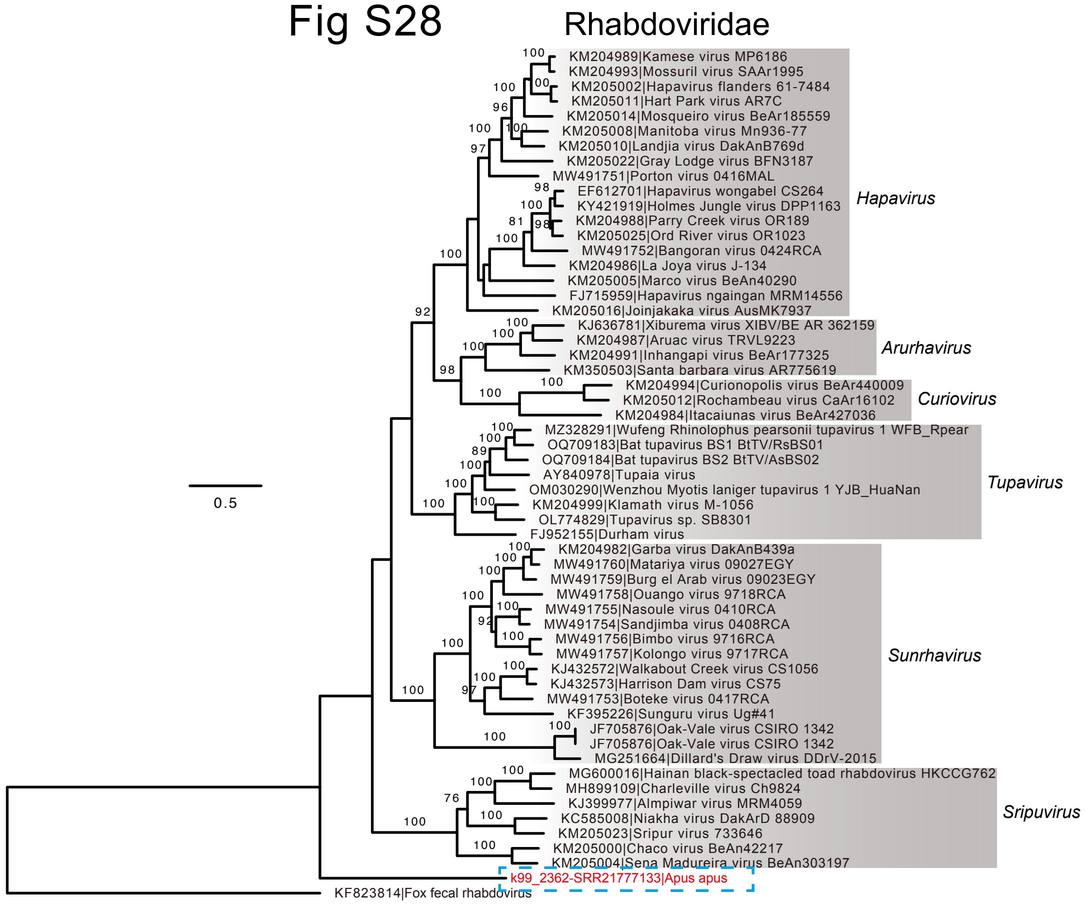
Rhabdoviridae
KM204989|Kamese virus MP6186
KM204993|Mossuril virus SAAr1995
KM205002|Hapavirus flanders 61-7484
KM205011|Hart Park virus AR7C
KM205014|Mosqueiro virus BeAr185559
KM205008|Manitoba virus Mn936-77
KM205010|Landjia virus DakAnB769d
KM205022|Gray Lodge virus BFN3187
MW491751|Porton virus 0416MAL
EF612701|Hapavirus wongabel CS264
KY421919|Holmes Jungle virus DPP1163
KM204988|Parry Creek virus OR189
KM205025|Ord River virus OR1023
MW491752|Bangoran virus 0424RCA
KM204986|La Joya virus J-134
KM205005|Marco virus BeAn40290
FJ715959|Hapavirus ngaingan MRM14556
KM205016|Joinjakaka virus AusMK7937
Hapavirus
KJ636781|Xiburema virus XIBV/BE AR 362159
KM204987|Aruac virus TRVL9223
KM204991|Inhangapi virus BeAr177325
KM350503|Santa barbara virus AR775619
Arurhavirus
KM204994|Curionopolis virus BeAr440009
KM205012|Rochambeau virus CaAr16102
KM204984|Itacaiunas virus BeAr427036
Curiovirus
MZ328291|Wufeng Rhinolophus pearsonii tupavirus 1 WFB_Rpear
OQ709183|Bat tupavirus BS1 BtTV/RsBS01
OQ709184|Bat tupavirus BS2 BtTV/AsBS02
AY840978|Tupaia virus
OM030290|Wenzhou Myotis laniger tupavirus 1 YJB_HuaNan
KM204999|Klamath virus M-1056
OL774829|Tupavirus sp. SB8301
FJ952155|Durham virus
Tupavirus
KM204982|Garba virus DakAnB439a
MW491760|Matariya virus 09027EGY
MW491759|Burg el Arab virus 09023EGY
MW491758|Ouango virus 9718RCA
MW491755|Nasoule virus 0410RCA
MW491754|Sandjimba virus 0408RCA
MW491756|Bimbo virus 9716RCA
MW491757|Kolongo virus 9717RCA
KJ432572|Walkabout Creek virus CS1056
KJ432573|Harrison Dam virus CS75
MW491753|Boteke virus 0417RCA
KF395226|Sunguru virus Ug#41
JF705876|Oak-Vale virus CSIRO 1342
JF705876|Oak-Vale virus CSIRO 1342
MG251664|Dillard's Draw virus DDrV-2015
Sunrhavirus
MG600016|Hainan black-spectacled toad rhabdovirus HKCCG762
MH899109|Charleville virus Ch9824
KJ399977|Almpiwar virus MRM4059
KC585008|Niakha virus DakArD 88909
KM205023|Sripur virus 733646
KM205000|Chaco virus BeAn42217
KM205004|Sena Madureira virus BeAn303197
Sripuvirus
k99_2362-SRR21777133|Apus apus
KF823814|Fox fecal rhabdovirus
0.5

Fig S29

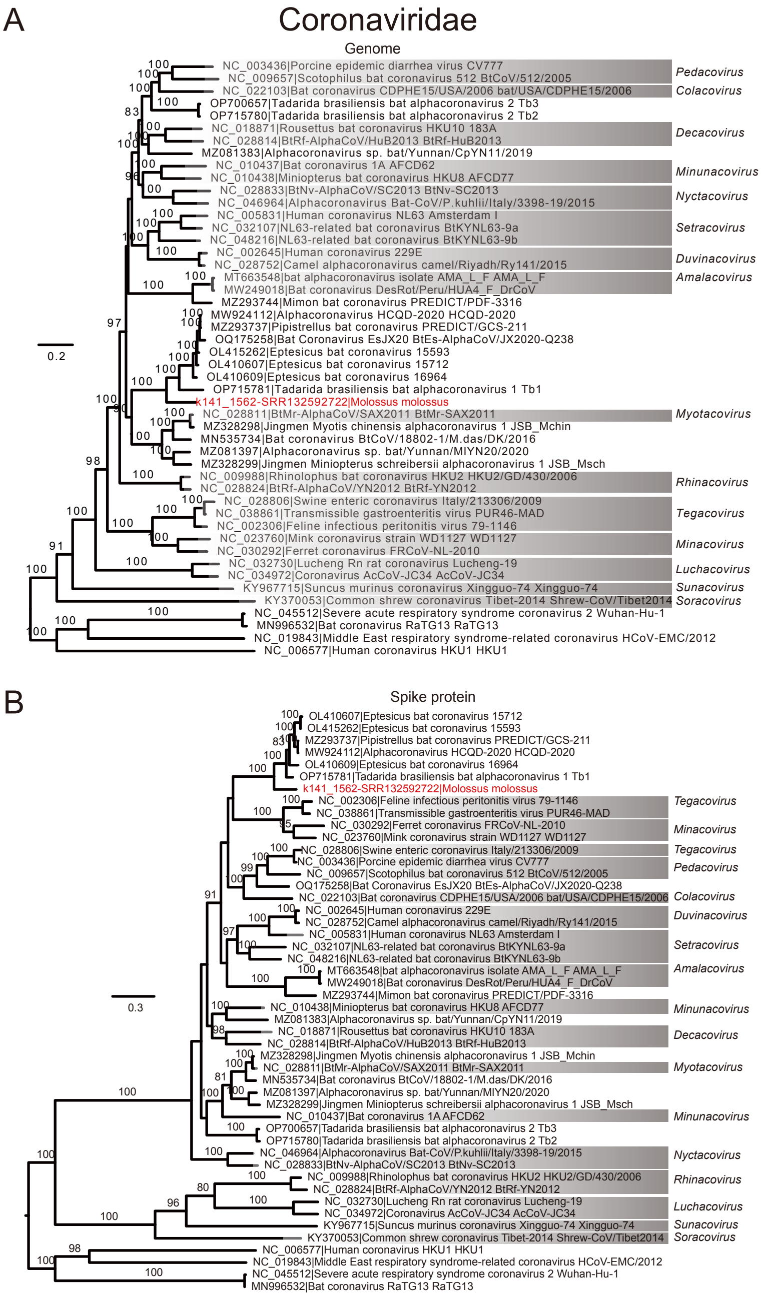
A
Coronaviridae
Genome
NC_003436|Porcine epidemic diarrhea virus CV777
NC_009657|Scotophilus bat coronavirus 512 BtCoV/512/2005
NC_022103|Bat coronavirus CDPHE15/USA/2006 bat/USA/CDPHE15/2006
OP700657|Tadarida brasiliensis bat alphacoronavirus 2 Tb3
OP715780|Tadarida brasiliensis bat alphacoronavirus 2 Tb2
NC_018871|Rousettus bat coronavirus HKU10 183A
NC_028814|BtRf-AlphaCoV/HuB2013 BtRf-HuB2013
MZ081383|Alphacoronavirus sp. bat/Yunnan/CpYN11/2019
NC_010437|Bat coronavirus 1A AFCD62
NC_010438|Miniopterus bat coronavirus HKU8 AFCD77
NC_028833|BtNv-AlphaCoV/SC2013 BtNv-SC2013
NC_046964|Alphacoronavirus Bat-CoV/P.kuhlii/Italy/3398-19/2015
NC_005831|Human coronavirus NL63 Amsterdam I
NC_032107|NL63-related bat coronavirus BtKYNL63-9a
NC_048216|NL63-related bat coronavirus BtKYNL63-9b
NC_002645|Human coronavirus 229E
NC_028752|Camel alphacoronavirus camel/Riyadh/Ry141/2015
MT663548|bat alphacoronavirus isolate AMA_L_F AMA_L_F
MW249018|Bat coronavirus DesRot/Peru/HUA4_F_DrCoV
MZ293744|Mimon bat coronavirus PREDICT/PDF-3316
MW924112|Alphacoronavirus HCQD-2020 HCQD-2020
MZ293737|Pipistrellus bat coronavirus PREDICT/GCS-211
OQ175258|Bat Coronavirus EsJX20 BtEs-AlphaCoV/JX2020-Q238
OL415262|Eptesicus bat coronavirus 15593
OL410607|Eptesicus bat coronavirus 15712
OL410609|Eptesicus bat coronavirus 16964
OP715781|Tadarida brasiliensis bat alphacoronavirus 1 Tb1
k141_1562-SRR132592722|Molossus molossus
NC_028811|BtMr-AlphaCoV/SAX2011 BtMr-SAX2011
MZ328298|Jingmen Myotis chinensis alphacoronavirus 1 JSB_Mchin
MN535734|Bat coronavirus BtCoV/18802-1/M.das/DK/2016
MZ081397|Alphacoronavirus sp. bat/Yunnan/MIYN20/2020
MZ328299|Jingmen Miniopterus schreibersii alphacoronavirus 1 JSB_Msch
NC_009988|Rhinolophus bat coronavirus HKU2 HKU2/GD/430/2006
NC_028824|BtRf-AlphaCoV/YN2012 BtRf-YN2012
NC_028806|Swine enteric coronavirus Italy/213306/2009
NC_038861|Transmissible gastroenteritis virus PUR46-MAD
NC_002306|Feline infectious peritonitis virus 79-1146
NC_023760|Mink coronavirus strain WD1127 WD1127
NC_030292|Ferret coronavirus FRCoV-NL-2010
NC_032730|Lucheng Rn rat coronavirus Lucheng-19
NC_034972|Coronavirus AcCoV-JC34 AcCoV-JC34
KY967715|Suncus murinus coronavirus Xingguo-74 Xingguo-74
KY370053|Common shrew coronavirus Tibet-2014 Shrew-CoV/Tibet2014
NC_045512|Severe acute respiratory syndrome coronavirus 2 Wuhan-Hu-1
MN996532|Bat coronavirus RaTG13 RaTG13
NC_019843|Middle East respiratory syndrome-related coronavirus HCoV-EMC/2012
NC_006577|Human coronavirus HKU1 HKU1
Pedacovirus
Colacovirus
Decacovirus
Minunacovirus
Nyctacovirus
Setracovirus
Duvinacovirus
Amalacovirus
Myotacovirus
Rhinacovirus
Tegacovirus
Minacovirus
Luchacovirus
Sunacovirus
Soracovirus
0.2
B
Spike protein
OL410607|Eptesicus bat coronavirus 15712
OL415262|Eptesicus bat coronavirus 15593
MZ293737|Pipistrellus bat coronavirus PREDICT/GCS-211
MW924112|Alphacoronavirus HCQD-2020 HCQD-2020
OL410609|Eptesicus bat coronavirus 16964
OP715781|Tadarida brasiliensis bat alphacoronavirus 1 Tb1
k141_1562-SRR132592722|Molossus molossus
NC_002306|Feline infectious peritonitis virus 79-1146
NC_038861|Transmissible gastroenteritis virus PUR46-MAD
NC_030292|Ferret coronavirus FRCoV-NL-2010
NC_023760|Mink coronavirus strain WD1127 WD1127
NC_028806|Swine enteric coronavirus Italy/213306/2009
NC_003436|Porcine epidemic diarrhea virus CV777
NC_009657|Scotophilus bat coronavirus 512 BtCoV/512/2005
OQ175258|Bat Coronavirus EsJX20 BtEs-AlphaCoV/JX2020-Q238
NC_022103|Bat coronavirus CDPHE15/USA/2006 bat/USA/CDPHE15/2006
NC_002645|Human coronavirus 229E
NC_028752|Camel alphacoronavirus camel/Riyadh/Ry141/2015
NC_005831|Human coronavirus NL63 Amsterdam I
NC_032107|NL63-related bat coronavirus BtKYNL63-9a
NC_048216|NL63-related bat coronavirus BtKYNL63-9b
MT663548|bat alphacoronavirus isolate AMA_L_F AMA_L_F
MW249018|Bat coronavirus DesRot/Peru/HUA4_F_DrCoV
MZ293744|Mimon bat coronavirus PREDICT/PDF-3316
NC_010438|Miniopterus bat coronavirus HKU8 AFCD77
MZ081383|Alphacoronavirus sp. bat/Yunnan/CpYN11/2019
NC_018871|Rousettus bat coronavirus HKU10 183A
NC_028814|BtRf-AlphaCoV/HuB2013 BtRf-HuB2013
MZ328298|Jingmen Myotis chinensis alphacoronavirus 1 JSB_Mchin
NC_028811|BtMr-AlphaCoV/SAX2011 BtMr-SAX2011
MN535734|Bat coronavirus BtCoV/18802-1/M.das/DK/2016
MZ081397|Alphacoronavirus sp. bat/Yunnan/MIYN20/2020
MZ328299|Jingmen Miniopterus schreibersii alphacoronavirus 1 JSB_Msch
NC_010437|Bat coronavirus 1A AFCD62
OP700657|Tadarida brasiliensis bat alphacoronavirus 2 Tb3
OP715780|Tadarida brasiliensis bat alphacoronavirus 2 Tb2
NC_046964|Alphacoronavirus Bat-CoV/P.kuhlii/Italy/3398-19/2015
NC_028833|BtNv-AlphaCoV/SC2013 BtNv-SC2013
NC_009988|Rhinolophus bat coronavirus HKU2 HKU2/GD/430/2006
NC_028824|BtRf-AlphaCoV/YN2012 BtRf-YN2012
NC_032730|Lucheng Rn rat coronavirus Lucheng-19
NC_034972|Coronavirus AcCoV-JC34 AcCoV-JC34
KY967715|Suncus murinus coronavirus Xingguo-74 Xingguo-74
KY370053|Common shrew coronavirus Tibet-2014 Shrew-CoV/Tibet2014
NC_006577|Human coronavirus HKU1 HKU1
NC_019843|Middle East respiratory syndrome-related coronavirus HCoV-EMC/2012
NC_045512|Severe acute respiratory syndrome coronavirus 2 Wuhan-Hu-1
MN996532|Bat coronavirus RaTG13 RaTG13
Tegacovirus
Minacovirus
Tegacovirus
Pedacovirus
Colacovirus
Duvinacovirus
Setracovirus
Amalacovirus
Minunacovirus
Decacovirus
Myotacovirus
Minunacovirus
Nyctacovirus
Rhinacovirus
Luchacovirus
Sunacovirus
Soracovirus
0.3

Fig S30

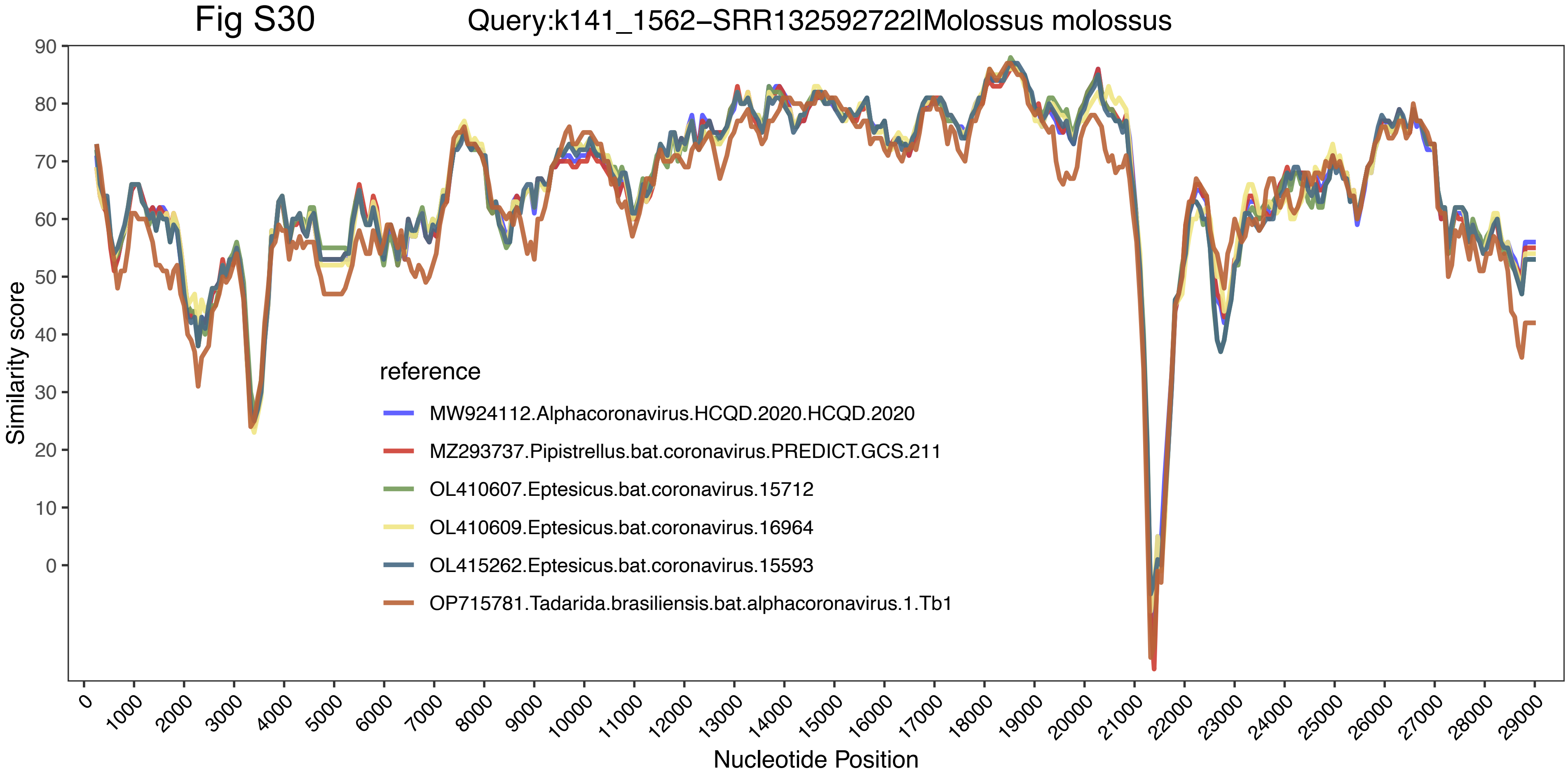
Query:k141_1562–SRR13259272|Molossus molossus
Similarity score
Nucleotide Position
reference
MW924112.Alphacoronavirus.HCQD.2020.HCQD.2020
MZ293737.Pipistrellus.bat.coronavirus.PREDICT.GCS.211
OL410607.Eptesicus.bat.coronavirus.15712
OL410609.Eptesicus.bat.coronavirus.16964
OL415262.Eptesicus.bat.coronavirus.15593
OP715781.Tadarida.brasiliensis.bat.alphacoronavirus.1.Tb1
0
10
20
30
40
50
60
70
80
90
0
1000
2000
3000
4000
5000
6000
7000
8000
9000
10000
11000
12000
13000
14000
15000
16000
17000
18000
19000
20000
21000
22000
23000
24000
25000
26000
27000
28000
29000

Fig S31

Picornaviridae

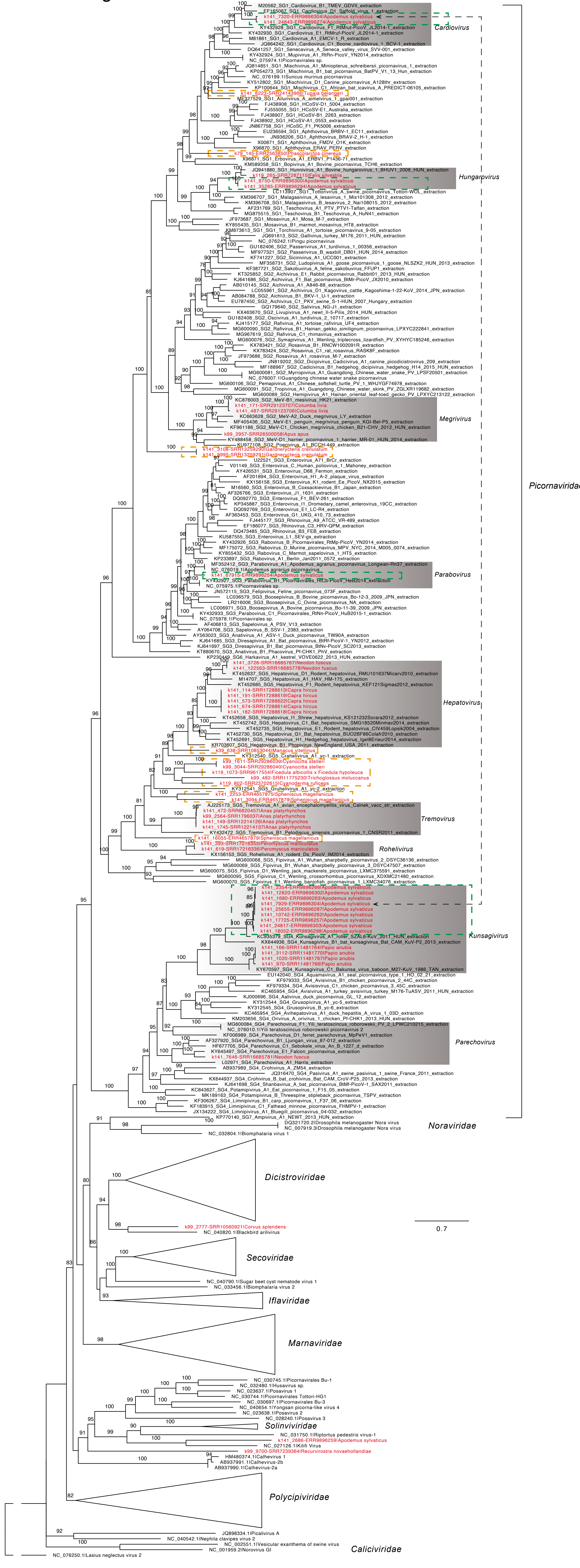

Fig S32

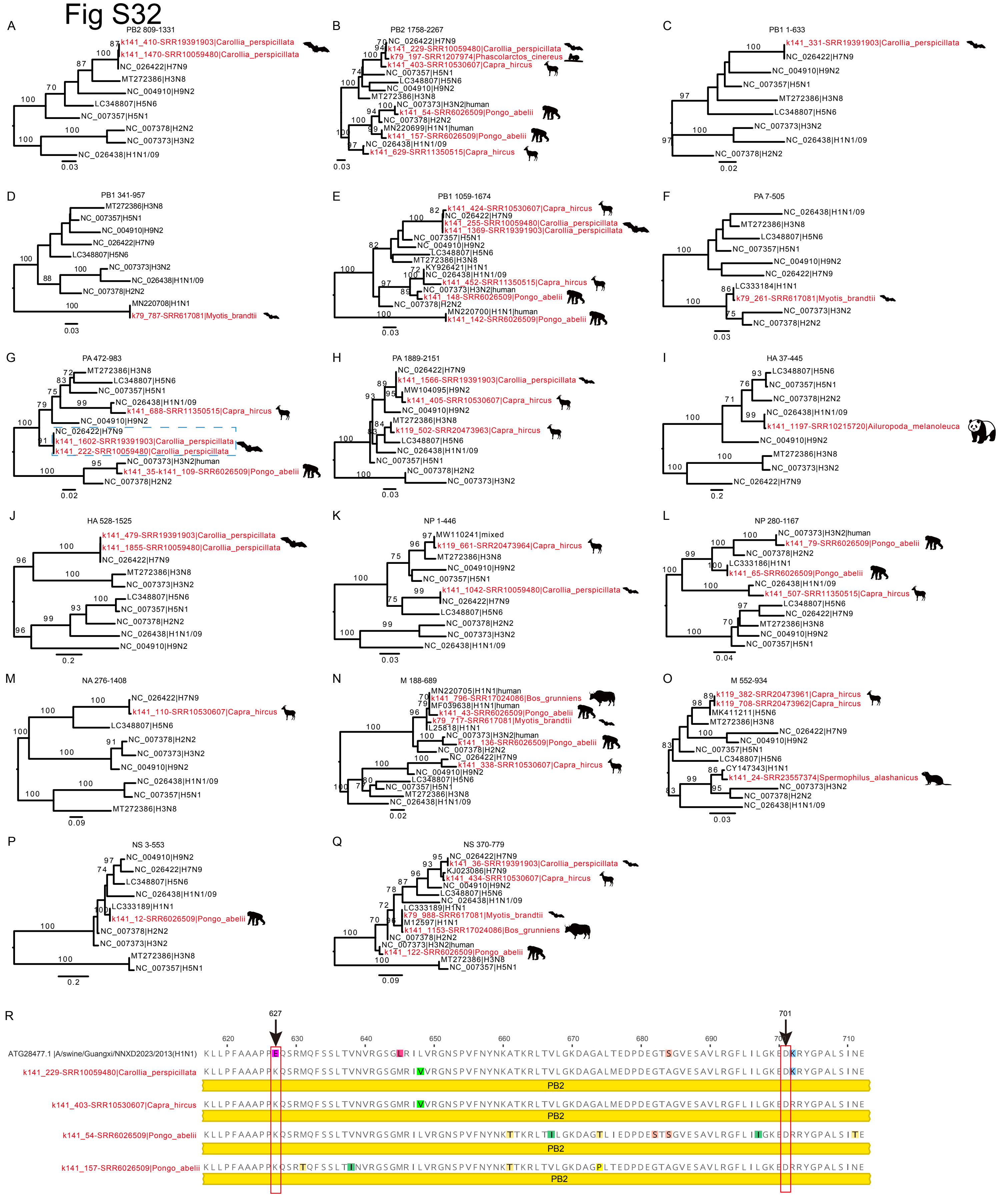
A
PB2 809-1331
B
PB2 1758-2267
C
PB1 1-633
D
PB1 341-957
E
PB1 1059-1674
F
PA 7-505
G
PA 472-983
H
PA 1889-2151
I
HA 37-445
J
HA 528-1525
K
NP 1-446
L
NP 280-1167
M
NA 276-1408
N
M 188-689
O
M 552-934
P
NS 3-553
Q
NS 370-779
R
ATG28477.1 |A/swine/Guangxi/NNXD2023/2013(H1N1)
k141_229-SRR10059480|Carollia_perspicillata
k141_403-SRR10530607|Capra_hircus
k141_54-SRR6026509|Pongo_abelii
k141_157-SRR6026509|Pongo_abelii
PB2
627
701

Fig S33

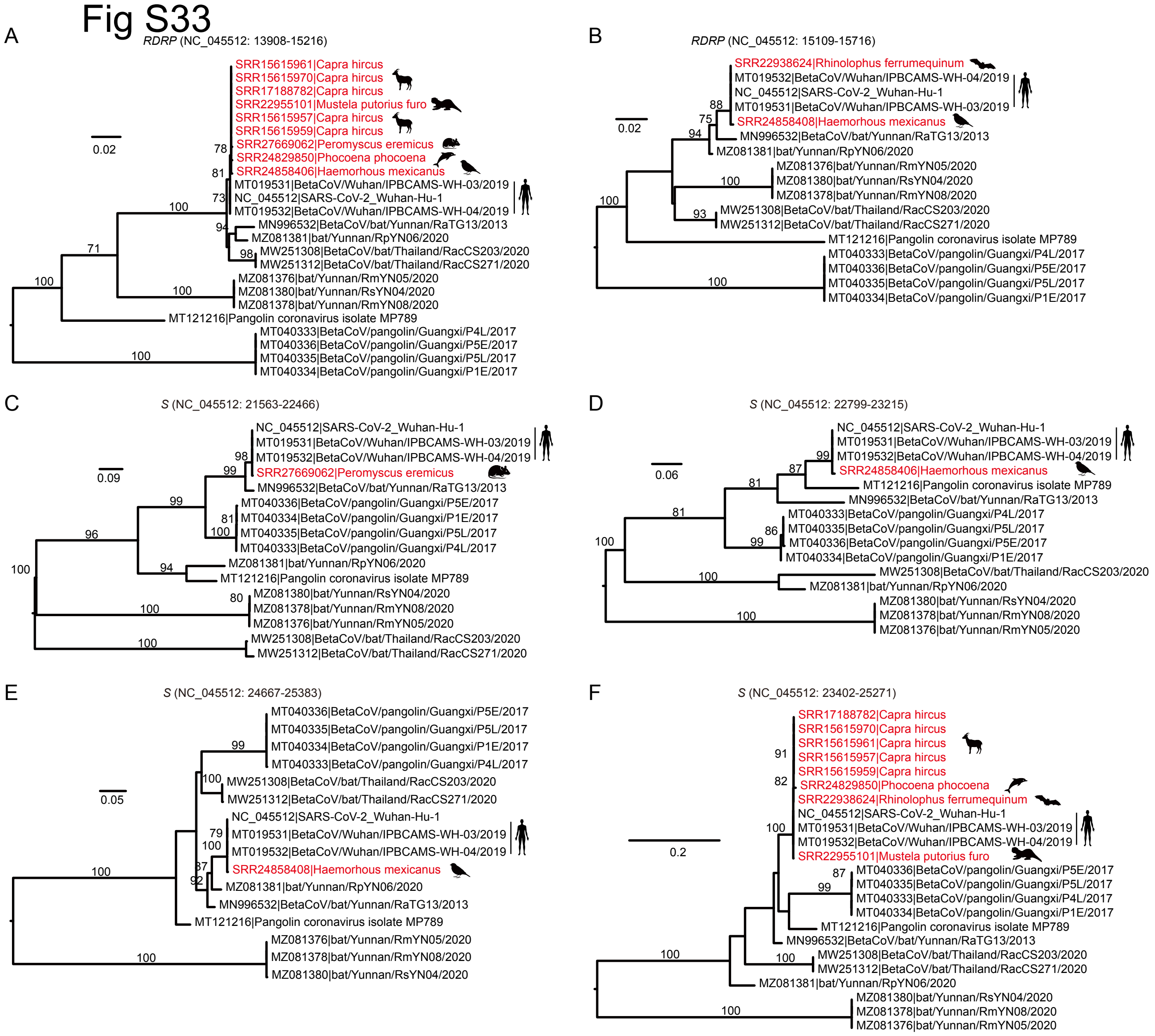
A
RDRP (NC_045512: 13908-15216)
SRR15615961|Capra hircus
SRR15615970|Capra hircus
SRR17188782|Capra hircus
SRR22955101|Mustela putorius furo
SRR15615957|Capra hircus
SRR15615959|Capra hircus
SRR27669062|Peromyscus eremicus
SRR24829850|Phocoena phocoena
SRR24858406|Haemorhous mexicanus
MT019531|BetaCoV/Wuhan/IPBCAMS-WH-03/2019
NC_045512|SARS-CoV-2_Wuhan-Hu-1
MT019532|BetaCoV/Wuhan/IPBCAMS-WH-04/2019
MN996532|BetaCoV/bat/Yunnan/RaTG13/2013
MZ081381|bat/Yunnan/RpYN06/2020
MW251308|BetaCoV/bat/Thailand/RacCS203/2020
MW251312|BetaCoV/bat/Thailand/RacCS271/2020
MZ081376|bat/Yunnan/RmYN05/2020
MZ081380|bat/Yunnan/RsYN04/2020
MZ081378|bat/Yunnan/RmYN08/2020
MT121216|Pangolin coronavirus isolate MP789
MT040333|BetaCoV/pangolin/Guangxi/P4L/2017
MT040336|BetaCoV/pangolin/Guangxi/P5E/2017
MT040335|BetaCoV/pangolin/Guangxi/P5L/2017
MT040334|BetaCoV/pangolin/Guangxi/P1E/2017
0.02
B
RDRP (NC_045512: 15109-15716)
SRR22938624|Rhinolophus ferrumequinum
MT019532|BetaCoV/Wuhan/IPBCAMS-WH-04/2019
NC_045512|SARS-CoV-2_Wuhan-Hu-1
MT019531|BetaCoV/Wuhan/IPBCAMS-WH-03/2019
SRR24858408|Haemorhous mexicanus
MN996532|BetaCoV/bat/Yunnan/RaTG13/2013
MZ081381|bat/Yunnan/RpYN06/2020
MZ081376|bat/Yunnan/RmYN05/2020
MZ081380|bat/Yunnan/RsYN04/2020
MZ081378|bat/Yunnan/RmYN08/2020
MW251308|BetaCoV/bat/Thailand/RacCS203/2020
MW251312|BetaCoV/bat/Thailand/RacCS271/2020
MT121216|Pangolin coronavirus isolate MP789
MT040333|BetaCoV/pangolin/Guangxi/P4L/2017
MT040336|BetaCoV/pangolin/Guangxi/P5E/2017
MT040335|BetaCoV/pangolin/Guangxi/P5L/2017
MT040334|BetaCoV/pangolin/Guangxi/P1E/2017
0.02
C
S (NC_045512: 21563-22466)
NC_045512|SARS-CoV-2_Wuhan-Hu-1
MT019531|BetaCoV/Wuhan/IPBCAMS-WH-03/2019
MT019532|BetaCoV/Wuhan/IPBCAMS-WH-04/2019
SRR27669062|Peromyscus eremicus
MN996532|BetaCoV/bat/Yunnan/RaTG13/2013
MT040336|BetaCoV/pangolin/Guangxi/P5E/2017
MT040334|BetaCoV/pangolin/Guangxi/P1E/2017
MT040335|BetaCoV/pangolin/Guangxi/P5L/2017
MT040333|BetaCoV/pangolin/Guangxi/P4L/2017
MZ081381|bat/Yunnan/RpYN06/2020
MT121216|Pangolin coronavirus isolate MP789
MZ081380|bat/Yunnan/RsYN04/2020
MZ081378|bat/Yunnan/RmYN08/2020
MZ081376|bat/Yunnan/RmYN05/2020
MW251308|BetaCoV/bat/Thailand/RacCS203/2020
MW251312|BetaCoV/bat/Thailand/RacCS271/2020
0.09
D
S (NC_045512: 22799-23215)
NC_045512|SARS-CoV-2_Wuhan-Hu-1
MT019531|BetaCoV/Wuhan/IPBCAMS-WH-03/2019
MT019532|BetaCoV/Wuhan/IPBCAMS-WH-04/2019
SRR24858406|Haemorhous mexicanus
MT121216|Pangolin coronavirus isolate MP789
MN996532|BetaCoV/bat/Yunnan/RaTG13/2013
MT040333|BetaCoV/pangolin/Guangxi/P4L/2017
MT040335|BetaCoV/pangolin/Guangxi/P5L/2017
MT040336|BetaCoV/pangolin/Guangxi/P5E/2017
MT040334|BetaCoV/pangolin/Guangxi/P1E/2017
MW251308|BetaCoV/bat/Thailand/RacCS203/2020
MZ081381|bat/Yunnan/RpYN06/2020
MZ081380|bat/Yunnan/RsYN04/2020
MZ081378|bat/Yunnan/RmYN08/2020
MZ081376|bat/Yunnan/RmYN05/2020
0.06
E
S (NC_045512: 24667-25383)
MT040336|BetaCoV/pangolin/Guangxi/P5E/2017
MT040335|BetaCoV/pangolin/Guangxi/P5L/2017
MT040334|BetaCoV/pangolin/Guangxi/P1E/2017
MT040333|BetaCoV/pangolin/Guangxi/P4L/2017
MW251308|BetaCoV/bat/Thailand/RacCS203/2020
MW251312|BetaCoV/bat/Thailand/RacCS271/2020
NC_045512|SARS-CoV-2_Wuhan-Hu-1
MT019531|BetaCoV/Wuhan/IPBCAMS-WH-03/2019
MT019532|BetaCoV/Wuhan/IPBCAMS-WH-04/2019
SRR24858408|Haemorhous mexicanus
MZ081381|bat/Yunnan/RpYN06/2020
MN996532|BetaCoV/bat/Yunnan/RaTG13/2013
MT121216|Pangolin coronavirus isolate MP789
MZ081376|bat/Yunnan/RmYN05/2020
MZ081378|bat/Yunnan/RmYN08/2020
MZ081380|bat/Yunnan/RsYN04/2020
0.05
F
S (NC_045512: 23402-25271)
SRR17188782|Capra hircus
SRR15615970|Capra hircus
SRR15615961|Capra hircus
SRR15615957|Capra hircus
SRR15615959|Capra hircus
SRR24829850|Phocoena phocoena
SRR22938624|Rhinolophus ferrumequinum
NC_045512|SARS-CoV-2_Wuhan-Hu-1
MT019531|BetaCoV/Wuhan/IPBCAMS-WH-03/2019
MT019532|BetaCoV/Wuhan/IPBCAMS-WH-04/2019
SRR22955101|Mustela putorius furo
MT040336|BetaCoV/pangolin/Guangxi/P5E/2017
MT040335|BetaCoV/pangolin/Guangxi/P5L/2017
MT040333|BetaCoV/pangolin/Guangxi/P4L/2017
MT040334|BetaCoV/pangolin/Guangxi/P1E/2017
MT121216|Pangolin coronavirus isolate MP789
MN996532|BetaCoV/bat/Yunnan/RaTG13/2013
MW251308|BetaCoV/bat/Thailand/RacCS203/2020
MW251312|BetaCoV/bat/Thailand/RacCS271/2020
MZ081381|bat/Yunnan/RpYN06/2020
MZ081380|bat/Yunnan/RsYN04/2020
MZ081378|bat/Yunnan/RmYN08/2020
MZ081376|bat/Yunnan/RmYN05/2020
0.2
